# Untargeted stable isotope-resolved metabolomics to assess the effect of PI3Kβ inhibition on metabolic pathway activities in a PTEN null breast cancer cell line

**DOI:** 10.3389/fmolb.2022.1004602

**Published:** 2022-10-14

**Authors:** Marcel Lackner, Sylvia K. Neef, Stefan Winter, Sandra Beer-Hammer, Bernd Nürnberg, Matthias Schwab, Ute Hofmann, Mathias Haag

**Affiliations:** ^1^ Dr. Margarete Fischer-Bosch-Institute of Clinical Pharmacology, Stuttgart, Germany; ^2^ University of Tübingen, Tübingen, Germany; ^3^ Department of Pharmacology, Experimental Therapy and Toxicology, Institute for Experimental and Clinical Pharmacology and Pharmacogenomics, Interfaculty Center for Pharmacogenomics and Drug Research (ICePhA), University of Tübingen, Tübingen, Germany; ^4^ Cluster of Excellence iFIT (EXC 2180), Image-Guided and Functionally Instructed Tumor Therapies, University of Tübingen, Tübingen, Germany; ^5^ Departments of Clinical Pharmacology and of Pharmacy and Biochemistry, University of Tübingen, Tübingen, Germany

**Keywords:** non-targeted analysis, 13C labeling, LC-QTOF-MS, glycolysis, TCA cycle, PI3K, cancer metabolism, stable isotope-resolved metabolomics (SIRM)

## Abstract

The combination of high-resolution LC-MS untargeted metabolomics with stable isotope-resolved tracing is a promising approach for the global exploration of metabolic pathway activities. In our established workflow we combine targeted isotopologue feature extraction with the non-targeted X^13^CMS routine. Metabolites, detected by X^13^CMS as differentially labeled between two biological conditions are subsequently integrated into the original targeted library. This strategy enables monitoring of changes in known pathways as well as the discovery of hitherto unknown metabolic alterations. Here, we demonstrate this workflow in a PTEN (phosphatase and tensin homolog) null breast cancer cell line (MDA-MB-468) exploring metabolic pathway activities in the absence and presence of the selective PI3Kβ inhibitor AZD8186. Cells were fed with [U-^13^C] glucose and treated for 1, 3, 6, and 24 h with 0.5 µM AZD8186 or vehicle, extracted by an optimized sample preparation protocol and analyzed by LC-QTOF-MS. Untargeted differential tracing of labels revealed 286 isotope-enriched features that were significantly altered between control and treatment conditions, of which 19 features could be attributed to known compounds from targeted pathways. Other 11 features were unambiguously identified based on data-dependent MS/MS spectra and reference substances. Notably, only a minority of the significantly altered features (11 and 16, respectively) were identified when preprocessing of the same data set (treatment *vs.* control in 24 h unlabeled samples) was performed with tools commonly used for label-free (i.e. w/o isotopic tracer) non-targeted metabolomics experiments (Profinder´s batch recursive feature extraction and XCMS). The structurally identified metabolites were integrated into the existing targeted isotopologue feature extraction workflow to enable natural abundance correction, evaluation of assay performance and assessment of drug-induced changes in pathway activities. Label incorporation was highly reproducible for the majority of isotopologues in technical replicates with a RSD below 10%. Furthermore, inter-day repeatability of a second label experiment showed strong correlation (Pearson *R* ^2^ > 0.99) between tracer incorporation on different days. Finally, we could identify prominent pathway activity alterations upon PI3Kβ inhibition. Besides pathways in central metabolism, known to be changed our workflow revealed additional pathways, like pyrimidine metabolism or hexosamine pathway. All pathways identified represent key metabolic processes associated with cancer metabolism and therapy.

## 1 Introduction

Reprogramming of energy metabolism is regarded as one of the hallmarks of cancer contributing to tumorigenesis and tumor progression. Metabolic alterations itself are involved in the regulation of gene or protein expression, thus metabolomics-based investigations are increasingly used for the assessment of cancer phenotypes or the characterization of drug response effects at a molecular level. Conventional metabolomics approaches however provide a rather static picture on metabolism, thus making the translation into physiologically meaningful mechanisms challenging. In this regard, the combination of metabolomics and stable isotope tracing (Jang et al., 2018) is a promising, complementary approach to explore cancer metabolism (Bruntz et al., 2017) and pathway activity changes in the absence or presence of pharmacological intervention (Fan et al., 2012; Fan et al., 2018; Lorkiewicz et al., 2019). Albeit experimentally more elaborated and technically demanding compared to conventional, label-free metabolomics, applying isotopic tracers bears some advantages including assisting in metabolite elemental composition assignment (Hegeman et al., 2007), differentiation of biological signals from background contaminants (Bueschl et al., 2013) and enables relative quantification *via* internal normalization (i.e. fractional abundance of each isotopologue normalized to the sum of all possible isotopologues). Altogether, these advantages can be beneficial in particular for non-targeted metabolomics experiments, that have to deal with a large number of metabolic features and require within-batch and between-batch correction (Brunius et al., 2016; Wehrens et al., 2016).

Metabolomics and isotopic tracing often relies on the targeted quantification of a subset of known compounds (Shi et al., 2020) which requires knowledge on the involved metabolic pathways beforehand. This, however leaves a large fraction of data unexplored which limits interpretation of metabolic effects including the generation of pathway models desired for more advanced metabolic flux analysis. In this regard, tracer fate detection in a non-targeted manner (Hiller et al., 2010; Weindl et al., 2015) has emerged as powerful tool to investigate large amounts of spectral data and to reveal pathway activity changes in regions of the metabolic network which were not expected (Creek et al., 2012). Various software tools have been developed to enable detection and quantification of isotopologues in an untargeted fashion in stable isotope labeled datasets derived from high-resolution LC/MS experiments. Despite their differences, a shared, important feature of all tools is the selection of potential candidate structures that, once identified, can be readily implemented into targeted data analysis workflows. This in turn improves alignment of isotopologue peaks through visual inspection, manual curation of peak areas as well as correction for isotope impurities and natural abundance. The latter aspect is particularly important (Midani et al., 2017) in that, if omitted or performed incorrectly it can result in distorted data (Heinrich et al., 2018). Such kind of data curation is expected to improve reproducibility of tracer experiments, an aspect that is equally important to well-established procedures for validation of spectral accuracy and precision of isotopic distribution (Heuillet et al., 2018; Schwaiger-Haber et al., 2019).

Here, we provide results of the development, assay performance evaluation and proof of concept application of a LC/MS based stable isotope-resolved metabolomics (SIRM) platform for the examination of isotope enrichment patterns in metabolites extracted from mammalian cell culture. Besides making use of a targeted library (i.e. on pre-defined pathways), the analysis was complemented with a subset of identified metabolites that could be revealed after comparing isotopologue distributions in labeled samples between drug treated and control conditions *via* the X^13^CMS tool (Huang et al., 2014; Llufrio et al., 2019).

We have chosen the PTEN-null triple negative breast cancer (TNBC) cell line MDA-MB-468 as a test system and investigated selective inhibition of PI3Kβ with AZD8186 (IC_50_ 4 nM) using [U-^13^C] glucose as a tracer. The PI3K/AKT pathway is one of the most important cellular signaling pathways and can be considered as a master regulator for cancer. Phosphatidylinositol 3-kinases (PI3K) are lipid kinases involved in many intracellular processes including cell metabolism, proliferation and survival. Once activated, they facilitate the transformation of phosphatidylinositol 4, 5-bisphosphate (PIP_2_) to the second messenger phosphatidylinositol 3, 4, 5-trisphosphate (PIP_3_) at the plasma membrane. PI3K signaling is closely linked to the tumor suppressor PTEN (phosphatase and tensin homolog deleted on chromosome 10) which dephosphorylates PIP_3_ back to PIP_2_ thus reverting the function of the PI3 kinases (Nürnberg and Beer-Hammer, 2021; White-Gloria et al., 2021). PTEN loss leads to upregulation of PI3K/Akt/mTOR signaling with a preferential activation of the PI3Kβ isoform in many tumor types (Zhang et al., 2017). Hence, PI3Kβ increases PIP_3_ formation resulting in enhanced cell proliferation and tumor progression (Bresnick and Backer, 2019; Nürnberg and Beer-Hammer, 2019). Consequently, several isoform-selective inhibitors have been developed (Ni et al., 2012) and are currently under investigation for the treatment of solid PTEN-null tumors (Hancox et al., 2015; Lynch et al., 2017; Sarker et al., 2021), partly with limited efficacy. For AZD8186 a recent phase I study demonstrated acceptable safety and tolerability as well as preliminary evidence of antitumor activity in monotherapy or combination therapy (Choudhury et al., 2022).

Until now, only limited data are available on the metabolic alterations caused by AZD8186 treatment in breast cancer. However, this may be desirable for evaluating resistance mechanisms and searching for metabolic vulnerabilities as potential targets for combination therapies. Previous experiments investigated the metabolic consequences of PI3Kβ inhibition with AZD8186 in HCC70 and LNCAP cells *in vitro* or in tumor xenografts (Lynch et al., 2017). Besides a downregulation of cholesterol biosynthesis genes and proteins the authors found that AZD8186 exposure upregulated PDHK4 (pyruvate dehydrogenase kinase) and increased PDH phosphorylation hence suggesting reduced carbon flux into the TCA cycle. This was supported by label-free non-targeted metabolomics revealing a reduction in nucleotide pools and changes in amino acids finally leading to an increase of cell stress. Similar effects of PI3K inhibition on carbon metabolism have been demonstrated by treatment of HCC1937 breast cancer cells with the pan-class I inhibitor BKM120 (also known as Buparlisib). BKM120 inhibits all four isoforms, i.e., PI3Kα,β,γ,δ, resulting in induction of DNA damage by impairing the production of ribose phosphate and amino acids needed for deoxynucleotide synthesis (Juvekar et al., 2016). Carbon flux studies further revealed that treatment affects the nonoxidative pentose phosphate pathway that delivers ribose-5-phosphate required for base ribosylation. Nevertheless, whether such pathways are similarly affected upon specific PI3Kβ inhibition with AZD8186 has not yet been investigated.

## 2 Materials and methods

### 2.1 Chemicals and Reagents

Ultra LC-MS grade acetonitrile (ACN) and methanol (MeOH) were purchased from Carl Roth GmbH & Co KG (Germany). Ammonium acetate (AmAc) was purchased from Sigma-Aldrich (Taufkirchen, Germany). Pure water was obtained from a Milli-Q system (Millipore, USA) and used for the preparation of aqueous solvents. Reference compounds were purchased from Sigma Aldrich (Taufkirchen, Germany) unless otherwise indicated (Supplementary Table S1).

### 2.2 Cell line cultivation

The PTEN-null TNBC cell line MDA-MB-468 (HTB-132, ATCC; USA) was cultivated in RPMI-1640 (Lonza, Switzerland) supplemented with 10% FBS (PAN Biotech, Germany) and 2 mM l-glutamine (Lonza, Switzerland) at 37 °C and 5% CO_2_ atmosphere. Passaging was done according to manufacturer’s specifications in a subcultivation ratio of 1:3 to 1:4, two times per week. The cell line was authenticated using STR profiling with PowerPlex^®^ 21 System (Promega, USA). Cells were used within 26 passages and were cultivated for less than 3 months.

### 2.3 Inhibitor

AZD8186 (Cayman Chemical, USA) was prepared as 2.000 µM stocks in sterile filtered DMSO (Merck, Germany) and stored under nitrogen (-192°C).

### 2.4 Stable isotopic labeling

Cells were seeded at a concentration of 7.5*10^5^ cells/well in 6-well plates (Greiner Bio-One, Germany) and left to adhere overnight in cultivation medium. After 16 h, the medium was replaced with fresh RPMI-1640 without FBS (Gibco, Germany). After 2 h of serum starvation, medium was exchanged to 11.1 mM [U-^13^C] glucose tracer (Cambridge Isotope Laboratories, USA) enriched RPMI-1640, supplemented with 0.5 µM AZD8186 or, respectively, vehicle. Samples were collected after 1, 3, 6, and 24 h. Parallel cultures, that were treated identically except that the medium contained naturally labeled glucose (Carl Roth, Germany), were used as unlabeled reference and were worked up after 24 h. For the evaluation of inter-day assay performance, the 1 and 3 h sampling time points (referenced as “Day 1”) were compared against corresponding sampling times of an independent labeling experiment that was performed on another day (referenced as “Day 2”) using the same cultivation and treatment conditions.

### 2.5 Sample processing - quench and extraction

Sampling of cultivated cells was done according to an optimized protocol for simultaneous quenching and extraction. Protocol optimization was carried out with respect to metabolism quenching *via* assessment of the adenylated energy charge (Atkinson and Walton, 1967), that was determined to be >0.85 for the quenching and metabolite extraction procedure. In short, medium was removed (i.e., by manual aspiration with pipette tip) and cells were quickly washed with prechilled (6°C) 1x PBS (phosphate buffered saline, Lonza, Switzerland). Plates were transferred onto dry ice and quenched with 600 μl, ultra-cool (- 80°C) MeOH/water (8:2, v/v). After that, cells were scraped off and left to extract on dry ice for 15 min. Cell lysis was achieved by repeated aspiration and dispensing in the well (2–3 times using 1.000 µl pipette tip), followed by transfer to polypropylene tubes (Eppendorf, Germany), and three freeze-thaw cycles on dry ice and iced-water. Cell suspensions were centrifuged at 4°C for 5 min at 15.000 rpm (Centrifuge type: 5424R, Eppendorf, Germany). The supernatant was transferred to new tubes and stored at - 80°C until analysis.

### 2.6 LC/MS analysis

For LC-MS analysis, 90 µl of cell extract was transferred into 96-well plates (Corning, USA), evaporated under N_2_ flow at 40°C, and reconstituted in 45 µl ACN/water (8:2, v/v). Samples were analyzed on an Agilent Infinity 1290 UHPLC coupled to a 6550 iFunnel quadrupole time-of-flight mass spectrometer (LC-QTOF-MS) from Agilent Technologies equipped with a Dual Agilent Jet Stream electrospray source. The system was operated by the Mass Hunter Data Acquisition Software (version B.08.00, Agilent Technologies, Germany). Chromatographic separation of cell extracts was performed using the zwitterionic InfinityLab Poroshell 120 HILIC-Z column (100 mm × 2.1 mm, 2.7 µm particle size, peek lined, Agilent Technologies, Germany) with an UHPLC Guard (HILIC-Z, 5 mm × 2.1 mm, 2.7 µM particle size, Agilent Technologies, Germany). The autosampler was operated at 6°C, the column oven at 35°C, and the injection volume was 2 µl. The column was operated at a flowrate of 0.25 ml/min using mobile phase A (10 mM AmAc in water, pH 7 with 5 µM InfinityLab Deactivator Additive (Agilent Technologies, Germany)) and mobile phase B (10 mM AmAc in water/ACN 1:9 (v/v), pH 7 with 5 μM InfinityLab Deactivator Additive). Deactivator Additive (medronic acid) was added to the mobile phase to improve peak shape and detection sensitivity of analytes that interact with stainless steel (e.g., phosphorylated nucleotides, organic acids) (Hsiao et al., 2018). After sample injection, the column was kept for 2 min at 90% B followed by gradient elution from 2–12 min (90–60% B). Subsequently, the column was maintained at 60% B for 3 min. The column returned to the initial condition from 15–16 min and re-equilibrated for 8 min at 90% B before injection of the next sample. Needle wash with 80% ACN in water (8:2, v/v) was applied between injections. The total run time was 24 min per sample. Fragment spectra were acquired from the unlabeled, pooled cell extracts by auto MS/MS as described earlier (Leuthold et al., 2017).

### 2.7 Targeted stable isotope-resolved metabolomics data analysis

Establishment of the targeted SIRM data preprocessing workflow was based on the VistaFlux Software package (Agilent Technologies, Germany), comprised of MassHunter PCDL Manager (version B.07.00, build 7024.35, service pack 2), MassHunter Pathway to PCDL (version B.07.00, build 19), MassHunter Profinder (version 10.0, build 10.0.10062.0), and Omix Premium (version 1.9.30). In a first step, a reference library (.cdb) of metabolites from central carbon and amino acid metabolism, as well as intermediates from *de novo* ribonucleotide biosynthesis was generated using MassHunter Pathway to PCDL. Corresponding standards of these metabolites were purchased and prepared as stocks. These compounds were diluted 1:1000 with ACN/water (8:2, v/v) for LC-MS analysis and investigated for their specific retention times (RTs) after chromatographic separation. Resulting retention times were added to the reference library using MassHunter PCDL Manager.

Acquired data from the tracer experiments were preprocessed with MassHunter Profinder using the “Batch Isotopologue Extraction” workflow and the established reference library. The default negative ion species was set to -H, charge state to 1, and the isotope purity corresponded to 99%. In addition, the extracted ion chromatogram (EIC) was smoothed prior integration using Gaussian smoothing with a function width of nine points and Gaussian width of 5.000 points. The peak core area was selected as ion abundance criterion and set to 20% of peak height. The mass window corrresponded to 15 ppm +2 mDa, while the retention time window was 0.2 min. For isotopologue ion thresholds the anchor ion height was set to ≥250 counts and the sum of ion heigths to ≥1,000 counts. The correlation coefficient of the coelution threshold corresponded to ≥0.50. After curation (i.e. manual reintegration of falsely integrated peaks) of EICs, results were exported as “Detailed csv” and subsequently imported into R studio (R version 4.0.3) for visualization and statistical analysis. The imported data was processed using the tidyverse package (Wickham et al., 2019), while diagrams and other graphics were generated from the ggplot2 and eulerr package, respectively (Wickham, 2016; Larsson, 2021). For statistical analysis, the incorporation of tracer into isotopologues between control and treatment was tested with a Welch’s *t*-test followed by correction for multiple testing *via* the Benjamini–Hochberg procedure (Benjamini and Hochberg, 1995). An adjusted *p*-value <0.05 was considered significant.

### 2.8 Untargeted stable isotope-resolved metabolomics data analysis

Untargeted analysis of ^13^C labeled features was done using the X^13^CMS approach as previously described (Huang et al., 2014; Llufrio et al., 2019) with method details summarized in Table 1. In short, raw data files (.d) were firstly transformed into .mzXML files by msConvert selecting vendor specific peak picking (Chambers et al., 2012; Adusumilli and Mallick, 2017). Converted files from unlabeled and labeled samples were subsequently processed by R Studio based XCMS (XCMS version 3.12.0, (Smith et al., 2006; Tautenhahn et al., 2008; Benton et al., 2010)). Here peaks were selected using the “centWave” method and corrected for their retention times employing the “obiwarp”-method and saved as the “xcmsSet” object. This object contained grouped and retention-time aligned features of both, unlabeled and labeled samples (Huang et al., 2014). The unlabeled reference samples were required to identify the base peak (lowest *m/z* of a pair of isotopologues) of labeled features. From the xcmsSet object, X^13^CMS extracted significantly labeled features (Welch’s *t*-test comparing relative intensities of unlabeled *vs.* labeled samples for each isotopologue) in control and treatment (getIsolabelReport), where the tolerance parameters, i.e. mass error (ppm), noise cutoff, and retention time window, had to be fulfilled (see Table 1 for further details). Labeled features were further investigated for significant changes in the labeling patterns between control and AZD8186 treatment by the filterIsoDiffReport function (Table 1).

**TABLE 1:** Parameter non-targeted SIRM.

XCMS Functions		
centWave^a^	ppm	10
	Peakwidth	c (10,60)
OBIwarp^b^	Profstep	0.9
	bw	5
	mzwid	0.015
Minidiffreport^c^	varEq	FALSE
	intChoice	intb

X^13^CMS functions		
getIsolabelReport^d^	RTwindow	10
	ppm	15
	noiseCutoff	100.000
	intChoice	intb
	alpha	0.05
getIsoDiffReport^e^	varEq =	FALSE
	singleSample	FALSE
filterIsoDiffReport^f^	alpha	0.05

^a^ Feature detection method (Tautenhahn et al., 2008).

^b^ Retention-time correction method (Prince and Marcotte, 2006).

^c^ Generates output table comparing individual features in unlabeled samples of vehicle and AZD8186 treated cells.

^d^ Identifies features that are significantly labeled in comparison to unlabeled samples for the corresponding condition (i.e. control and perturbation).

^e^ Creates a differential labeling report to compare labeling differences between experimental conditions (i.e. vehicle and AZD8186 treated cells).

^f^ Reports isotopologue groups from isoDiff report that are significantly different between experimental condition.

### 2.9 Metabolite identification from unlabeled samples

For metabolite identification, the final list of filtered features was investigated for corresponding fragment spectra from the auto MS/MS analysis of unlabeled samples with Mass Hunter Qualitative Analysis (Version B.07.00, Agilent Technologies). Spectral information (accurate mass and fragment ions) of these unlabeled MS/MS data was compared against spectral information from the METLIN Metabolite PCDL (Version B.07.00, Agilent Technologies). Identified compounds from database search were then confirmed after spectral comparison of respective pure substances in order to achieve MSI level 1 annotation (Sumner et al., 2007; Salek et al., 2013). Newly identified and confirmed metabolites were then integrated into the established targeted SIRM workflow by adding sum formula and retention time information to the reference library.

### 2.10 Untargeted data analysis of unlabeled samples with XCMS functions integrated within X^13^CMS

Unlabeled features were extracted from the “xcmsSet” object using the “Minidiffreport ()” command. Here, the baseline corrected integrated peak intensities were exported and further processed in R Studio. Zero values were replaced by 1/4 of the minimum positive value in the original data set. Features were then filtered according to a CV cutoff of 20% between technical replicates (n = 3) and subsequently sum normalized (peak area of each feature divided by the sum of peak areas of all features in one sample). Data was then log2-transformed and treatment effects between vehicle and AZD8186 treated samples were statistically evaluated using a Welch’s *t*-test with correction for multiple testing (Benjamini and Hochberg, 1995). Finally, only features with an adjusted *p*-value of <0.05 and a FC < 0.8 or FC > 1.2 of the mean area between treatment and vehicle were retained for comparison of the different untargeted workflows.

### 2.11 Untargeted data analysis of unlabeled samples with MassHunter Profinder

The unlabeled raw data files (.d), used as reference for the X^13^CMS workflow, were preprocessed with MassHunter Profinder Software (version B.08.00, Agilent Technologies) as previously described (Neef et al., 2020). In brief, features were obtained using “Batch recursive feature extraction” with an intensity threshold of 1000 counts and deprotonated molecular ions (H^−^) as adducts. In further detail, RT tolerance was set to ± 0.3 min, and a mass tolerance of ± 15 ppm + 2 mDa for the binning and alignment of features. The EIC range corresponded to ± 35 ppm and peak integration was performed with the Agile two algorithm. Peak spectra with a saturation above 30% were excluded from analysis. After curation of EICs, integrated peak areas were exported as comma separated values (.csv) and further processed using R Studio. Features were firstly filtered with a CV value <20% between the technical triplicates and subsequent sum normalized and log2-transformed. Statistical testing was done with a Welch’s *t*-test comparing vehicle and AZD8186 treated cells with subsequent correction for multiple testing by the Benjamini–Hochberg procedure. At last, features were filtered according to an adjusted *p*-value of <0.05 and FC < 0.8 or FC > 1.2 of the mean area between treatment and vehicle.

### 2.12 Comparison label-free untargeted metabolomics with untargeted stable isotope labeling

Features obtained after filtering from untargeted analyses of unlabeled samples with XCMS and Profinder and those obtained from the X^13^CMS workflow were imported and processed with R Studio. Overlaps were determined by aligning respective features by mass and retention time with a mass window of m/z ± 0.1 and retention time window ± 5 s. Principal component analysis (PCA) was performed with R function prcomp using default settings after sum normalization and log2 transformation based on feature lists from X^13^CMS (“IsoLabelReport”) and those obtained from the “Minidiffreport ()” and after untargeted data pre-processing with Profinder.

## 3 Results

### 3.1 Established SIRM workflow

**FIGURE 1:**
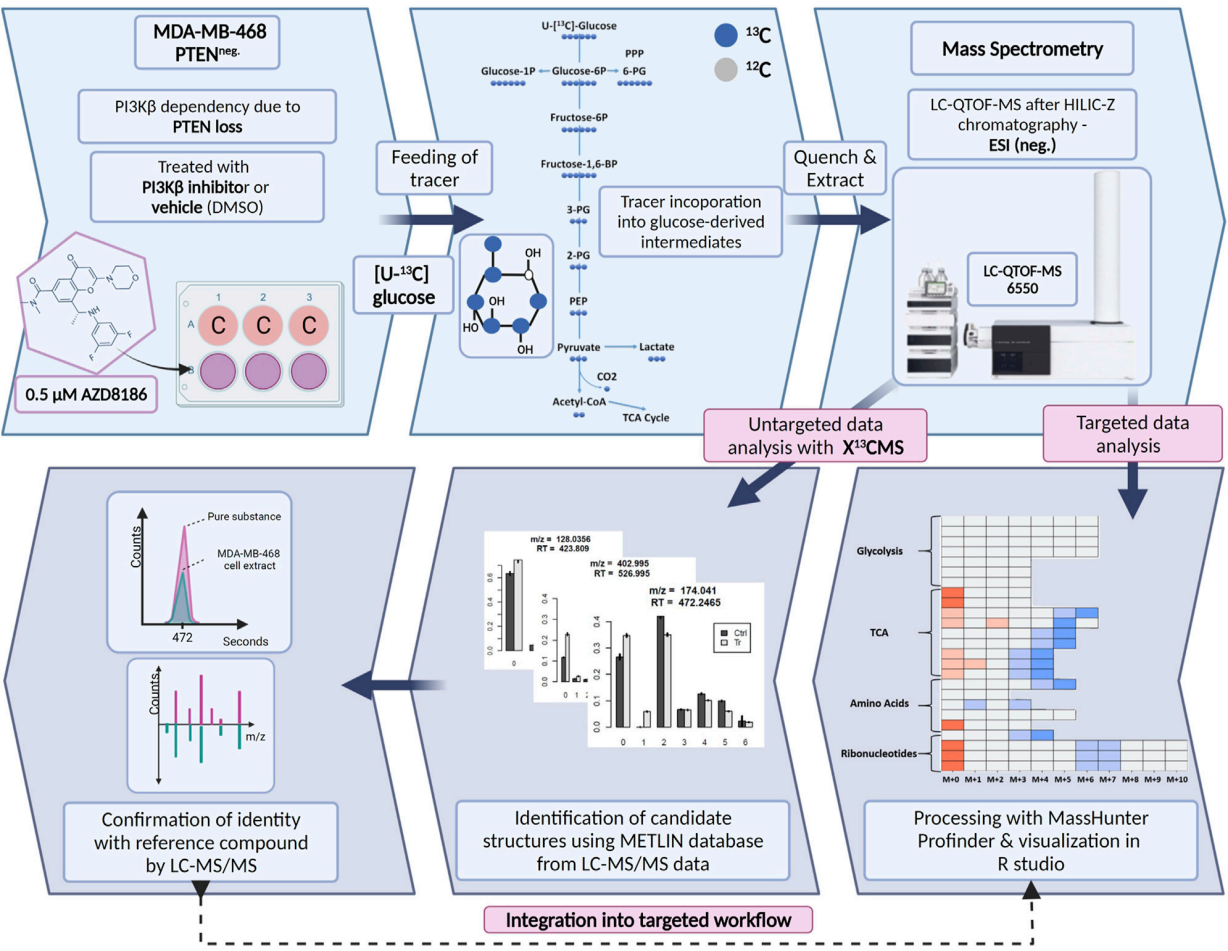
Schematic of SIRM workflow for tracer experiments. Cells were cultivated in [U- ^13^C] glucose tracer containing RPMI medium supplemented with 0.5 µM AZD8186 or vehicle. After 1, 3, 6, and 24 h cells were harvested according to an optimized quench and extraction protocol. Cell extracts were measured with LC-QTOF-MS and the obtained data analyzed in a targeted as well as untargeted manner. Targeted data analysis was performed with the MassHunter Profinder software environment and harnessed a metabolite library with corresponding sum formulas, masses, and retention times. Untargeted analysis with X^13^CMS (Huang et al., 2014) produced a list of differentially labeled metabolites between drug treated and control conditions. Fragment spectra from unlabeled cell extracts allowed the assignment of potential candidate structures from the X^13^CMS feature list. Metabolite identity was confirmed by the corresponding pure substance, its retention time, and fragment spectra. Finally, the identified metabolites were integrated into the targeted SIRM workflow.

We established a stable isotope-resolved metabolomics (SIRM) workflow from cell culture to data analysis and visualization in R enabling the estimation of metabolic alterations between two different biological conditions. To assess drug-induced metabolic changes upon PI3Kβ inhibition the workflow was applied to MDA-MB-468 cells treated with the selective inhibitor AZD8186 or vehicle using [U-^13^C] glucose as tracer (Figure 1). Cell extracts from time series experiments were measured by untargeted high-resolution LC-mass spectrometry and acquired data were processed using two different approaches. The first approach was based on an established targeted library of preselected intermediates from the central carbon metabolism, amino acid metabolism and ribonucleotide biosynthesis while the second approach harnessed the untargeted data analysis capabilities of the R based package X^13^CMS (Huang et al., 2014). Resulting candidate structures from untargeted analysis were confirmed by matching retention time and fragment spectra of corresponding reference compounds. These newly identified metabolites complemented the existing targeted library and were then integrated into the targeted data analysis for a detailed assessment of drug-induced alterations in pathway activities.

### 3.2 Untargeted data analysis with X^13^CMS

**FIGURE 2:**
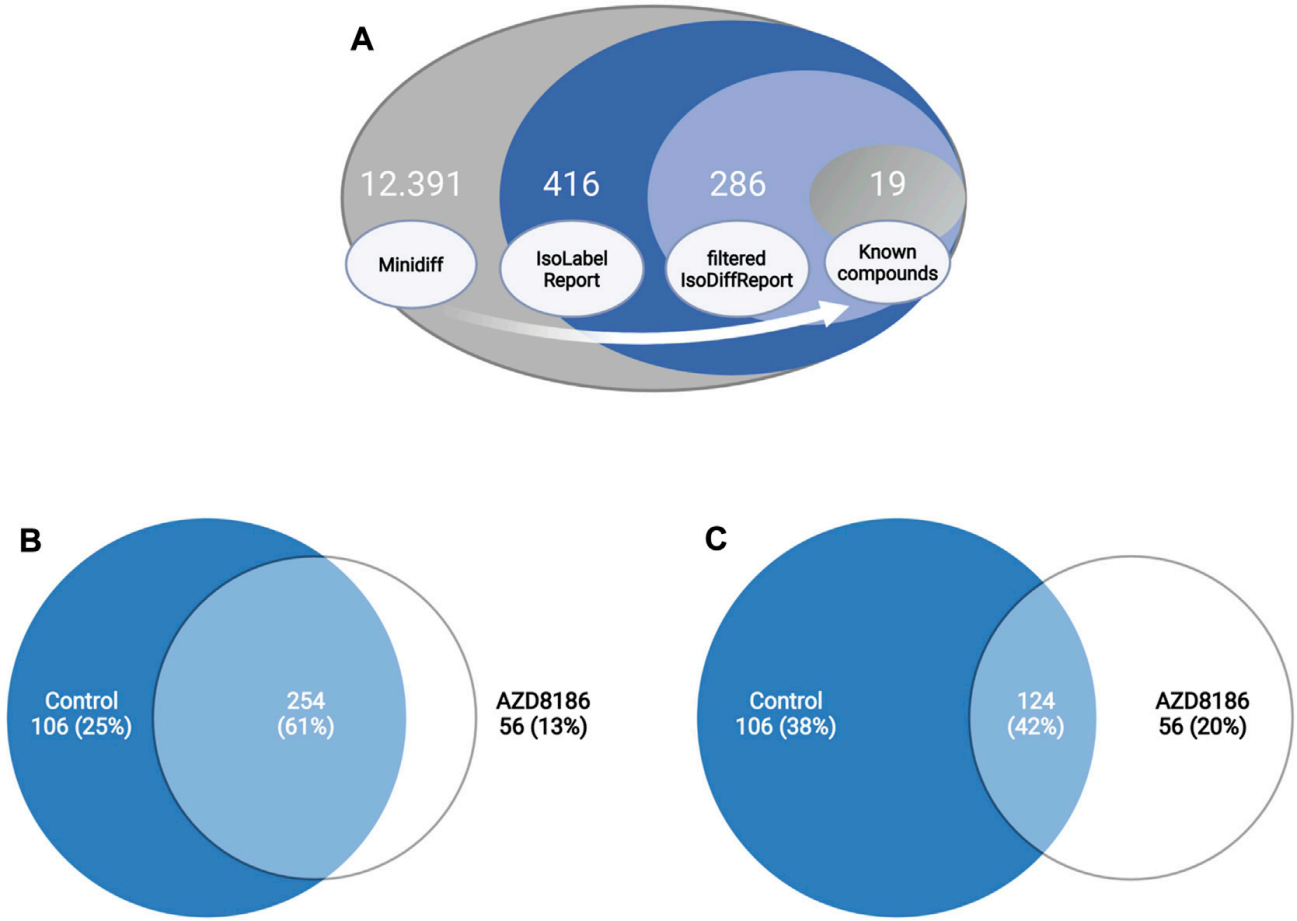
Differential labeling analysis of MDA-MB-468 cells treated for 24 h with vehicle or 0.5 µM AZD8186. Schematic display of the feature filtering procedure with the number of features obtained in the different processing steps **(A)**. Euler diagram of total labeled isotopologue groups in vehicle and AZD8186 treated cells (IsoLabelReport; Supplementary Table S4) **(B)**. Euler diagram of differentially labeled isotopologue groups between vehicle and AZD8186 treated cells (filterIsoDiffReport; Supplementary Table S5) **(C)**.

Features with differential labeling patterns between control and AZD8186 treated cells were detected with X^13^CMS using a multi-step process (Figure 2A). In the first step, a base peak list used for the identification of labeled features is extracted from the unlabeled reference samples with the miniDiffReport () function within the X^13^CMS package. Here, 12.391 unique features with corresponding *m/z* values and retention times (Supplementary Table S3) were identified. From that, the IsoLabelReport function yielded 416 unique ^13^C-enriched isotopologue groups reported as 360 and 310 compounds identified in control and AZD8186 treated cells, respectively (Supplementary Table S4). Of these, the majority (254) was found in both conditions, while 106 (25%) features were solely detectable in the control cells and 56 (13%) in the AZD8186 treated cells (Figure 2B). After comparison between the two conditions with IsoDiffReport, 286 features displayed significant changes in their labeling pattern upon PI3Kβ inhibition compared to control (Supplementary Table S5). Of these, 106 and 56 features were solely found in the control and treatment condition, respectively, while the common pool of features was reduced to 124 features (Figure 2C).

### 3.3 Metabolite identification

From the significantly altered features determined by the untargeted data analysis filtering procedure (Figure 2A), 19 could be attributed to known compounds from the established targeted library (i.e. TCA cycle, glycolysis, and ribonucleotide biosynthesis). Further 11 metabolites were identified from their respective fragment spectra with corresponding pure reference compounds. These identified metabolites were associated with the ribonucleotide biosynthesis (UDP, UTP), the mevalonate pathway (3H3MG), amino acid (NAA, pyroglutamic acid) and glutathione (GSH, GSSG) metabolism, as well as the hexosamine pathway (UDP-GlcNAc, UDP-Glucose, CMP-NANA, NANA) and were added to the preexisting library (Supplementary Table S1). Thus, metabolites identified by the untargeted X^13^CMS analysis allowed the extension to assess AZD8186 treatment effects within pyrimidine biosynthesis, mevalonate and hexosamine pathways.

### 3.4 Comparison of label-free untargeted metabolomics with untargeted stable isotope labeling

Comparison of results between the two workflows was done based on 1) the discriminatory characteristics of features derived from the different data pre-processing routines and 2) with respect to the overlap of significantly (i.e. adj. *p*-value <0.05) and relevantly (FC < 0.8 or, FC > 1.2) altered features between treatment and control groups. As depicted in the PCA (Supplementary Figures S1A–C), clear differentiation of control and AZD8186 treated cells was evident for all three data preprocessing workflows. Notably, despite the different number of features, group discrimination occurred in a comparable fashion for data preprocessing with X^13^CMS, XCMS and Profinder (Supplementary Figures S1A–C, respectively) that all displayed 24–26% of variability explained in the second component (i.e., PC2). Regarding differentially altered features between the two groups we pursued a similar approach as done by Huang *et al.* (Huang et al., 2014) who assessed outputs related to biological perturbation from unlabeled and labeled samples that were prepared in parallel (i.e. 24 h samples in our experiment) and acquired in the same LC/MS profiling run. To this end we compared the 286 features obtained from untargeted isotope labeling with unlabeled features from the miniDiffReport () and after MassHunter Profinder preprocessing of unlabeled samples. The miniDiffReport () function within the X^13^CMS package extracted 12.391 features from the unlabeled samples (Supplementary Table S3). After CV filtering (CV < 20% of three technical replicates), as well as *p*-value and fold change filtering (adjusted *p*-value <0.05, FC < 0.8 or, FC > 1.2), 322 features were significantly altered by the inhibitor (Supplementary Table S6). Preprocessing of unlabeled samples with MassHunter Profinder resulted in 490 features. After CV and *p*-value/fold change filtering similar to above (CV < 20%, adjusted *p*-value < 0.05, FC < 0.8 or, FC > 1.2), 20 features remained (Supplementary Table S7). Comparison of the feature recovery by the respective workflows displayed an overlap of 10 features for all three approaches (Supplementary Figures S1D). The processed miniDiffreport shared 16 features with labeled X^13^CMS workflow, whereof six features were exclusively recovered by both workflows, while the Profinder workflow shared 11 features with the X^13^CMS workflow with only one feature solely recovered by both workflows. Interestingly, label-free analysis with Profinder workflow only recovered features that were also covered by the XCMS workflow and/or the X^13^CMS workflow.

### 3.5 Robustness evaluation of SIRM workflow

**FIGURE 3:**
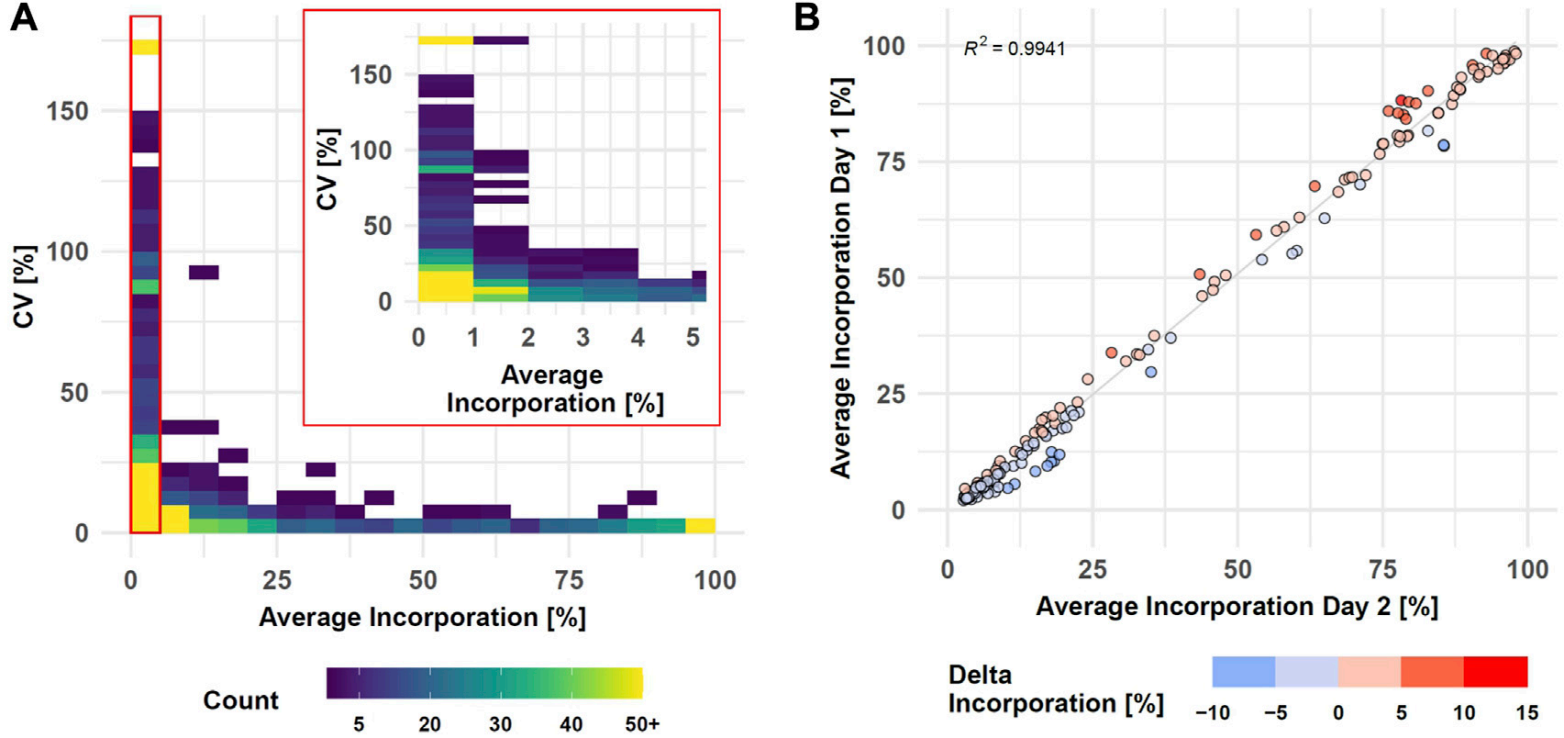
SIRM robustness evaluation after data preprocessing with targeted library (Supplementary Table S1). Intra-day reproducibility assessed by CVs of technical replicates (n = 3) compared to average incorporation as density plot (see Supplementary Table S8, Day 1 data set, samplings 1, 3, 6, and 24 h, 1585 isotopologues, binwidth = 5%) **(A)**. Inter-day reproducibility of SIRM experiments indicated as average tracer incorporation into 174 isotopologues on day 1 vs. day 2 (see Supplementary Table S9, tracer incorporation >2% and <100%) at 3 h sampling time point **(B)**.

The performance of the entire SIRM workflow was evaluated by comparison of the isotopologue distributions of 35 metabolites from the targeted library (Supplementary Table S1) utilizing the processing pipeline from MassHunter Profinder. Intra-day reproducibility was evaluated by technical replicates based on all 1585 isotopologues of the 35 metabolites comprising both, untreated and treated conditions as well as all sampling times (Figure 3A, Supplementary Table S8).

Tracer incorporation was highly reproducible for technical replicates with an average incorporation between 40 and 100% and a CV value below 5%. Only eight isotopologues out of 269 in this range had a CV value above 10%. The CV value increased with lower label incorporation. From 220 isotopologues in the range from 10 to 35% label incorporation, only three isotopologues displayed CV values above 25% while 26 isotopologues had a CV value between 10 and 25%. The remaining 191 isotopologues had a CV below 10% indicated by the high isotopologue count in this range. Only with tracer incorporations of less than 10% the data became more variable, and especially incorporations of less than 2% became highly imprecise. Here, 228 out of 762 isotopologues had a CV between 25 and 150% where further 81 isotopologues displayed a CV value of more than 150%. Although the variability with low levels of label incorporation (0–2%) increased, 453 from the 762 isotopologues still displayed high reproducibility with a CV value below 25% from which 223 even had a CV value below 10%.

Inter-day repeatability of the SIRM workflow was evaluated with an independent tracer experiment performed on a different day and the tracer incorporation dynamic into 33 out of 35 metabolites from the targeted library (Supplementary Table S1) of both experiments was compared for 3 and 1 h time points. As described above, low levels of tracer incorporation (<2%) can lead to higher variations of the respective data, while the presence of an m+0 isotopologue at a fractional labeling of 100% indicates an unlabeled molecule. Therefore, isotopologues with less than 2% or 100% incorporation were excluded from the correlation analysis. Overall, only six metabolites differed by more than 6% of the average incorporation between day one and day two at an incubation time of 3h, yielding a strong correlation (*R* ^2^ > 0.99) of the tracer incorporation on different days (Figure 3B, Supplementary Table S9). This positive association was confirmed by correlation analysis of isotopologues from the 1 h time point resulting in a *R* ^2^ > 0.99 (Supplementary Figures S2).

### 3.6 Integrated targeted SIRM workflow upon PI3Kβ inhibition

**FIGURE 4:**
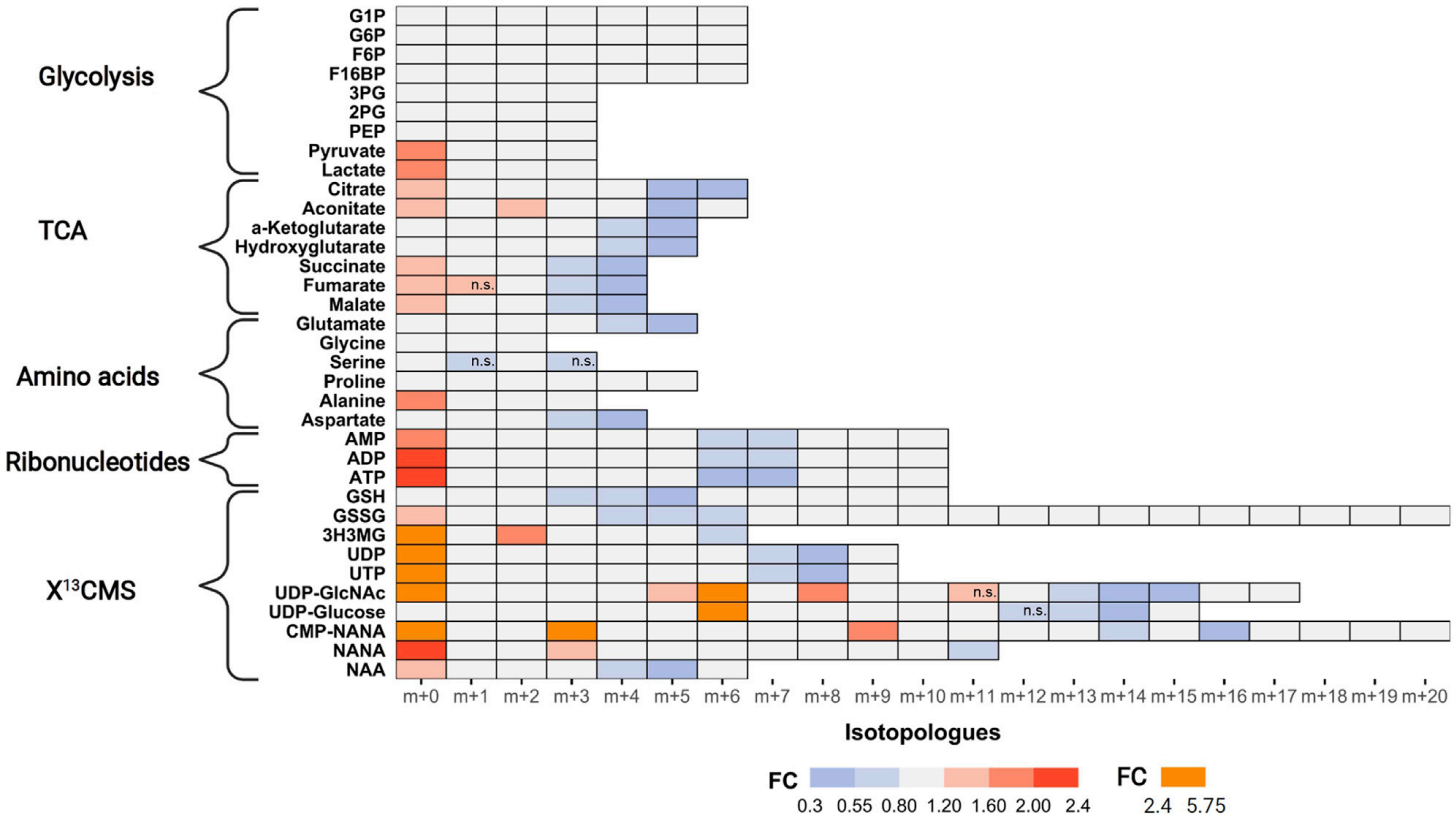
Impact of AZD8186 treatment on ^13^C tracer incorporation into different metabolic pathways. MDA-MB-468 cells were cultivated in RPMI with [U-^13^C] glucose and treated with 0.5 µM AZD8186 or vehicle for 24 h. Fold changes (AZD8186 vs. vehicle) of the individual isotopologues were calculated from the average labeling fraction of three technical replicates per condition and are represented by the indicated colour code. Only effects are displayed in the heatmap that exhibit a FC < 0.8 and >1.2 and tracer incorporation >3% (average of labeling fraction in control and treatment group). Information on alterations of isotopologues that do not fulfill these FC limits as well as information on results from statistical testing (adjusted *p*-value; Welch’s *t* test) can be obtained from Supporting PDF. Isotopologues that are not significantly altered are indicated (n.s.). Fractional abundance information is provided in Supplementary Table S2. G1P, Glucose-1 phosphate; G6P, Glucose-6 phosphate; F6B, Fructose-6,- phosphate; F16BP, Fructose-1,6,-bisphosphate; 3 PG, 3-Phosphoglycerate; 2 PG, 2-Phosphoglycerate; PEP, Phosphoenolpyruvate; 3H3MG, 3-Hydroxy-3-methylglutarate; AMP/ADP/ATP, Adenosine mono/di/triphosphate; GSH, glutathione; GSSG, oxidized glutathione; UDP/UTP, Uridine di/triphosphate; UDP-GlcNAc, Uridine diphosphate N-acetylglucosamine; NANA, N-acetylneuraminate; CMP-NANA, cytidine monophosphate N-acetylneuraminate; NAA, N-Acetylaspartate.

Next, we examined tracer incorporation in treated and untreated MDA-MB-468 cells for the 35 metabolites to investigate the impact of AZD8186 on specific metabolic pathways (Figure 4, Supplementary Table S2 and Supporting PDF). Incorporation of ^13^C tracer from glucose into glycolytic intermediates occurs within minutes. Consequently, the respective metabolites from G6P to PEP reach isotopic steady state with near complete labeling already at the first time point (1 h) making it impossible to assess drug induced changes of the glycolytic flux with these metabolites. Nevertheless, the labeling pattern of pyruvate with diminished fractional enrichment of the m+3 isotopologue (Supporting PDF) and corresponding increased proportion of the unlabeled m+0 isotopologue points to reduced glycolytic ^13^C-flux upon treatment. Lactate, as further end product of glycolysis, displayed a similar isotope pattern, thereby indicating a potential beneficial effect of PI3Kβ inhibition on aerobic glycolysis. Irrespective of pharmacological effects, the occurrence of only m+6 labeling in the hexose-6-phospate (i.e., F6B and F16BP) pool (Supporting PDF) indicates low basal level of fructose bisphosphatase activity (Lorkiewicz et al., 2019) in MDA-MB-468 cells. This is further supported by only trace quantities of m+3 labeling detected in metabolites along upper glycolysis (e.g. G6P). Interestingly, G1P, formed from G6P by phosphoglucomutase shows the most pronounced effect at 1 h (Supporting PDF). A strong decrease of the m+6 isotopologue accompanied by elevated m+0 indicates reduced substrate availability for glycogen or other downstream biosynthetic reactions (e.g. UDP-Glucose production).

TCA cycle metabolites display diminished enrichment (FC < 0.8) of isotopologues generated after multiple cycle rounds (>m+2) and elevated presence of the unlabeled m+0 isotopologues (FC > 1.2), reflecting reduced carbon flux into pyruvate upon treatment. Citrate and aconitate show high relative proportion (>45%) of the m+2 isotopologues at early labeling times (1 h), both in controls and treated cells (Supporting PDF), suggesting conversion to acetyl-CoA *via* pyruvate dehydrogenase (PDH) as the predominant route of carbon feeding into the cycle. Entry of ^13^C_3_-pyruvate as oxaloacetate *via* pyruvate carboxylase plays only a minor role as evidenced by a low proportion of the m+3 isotopologue in citrate and other TCA cycle intermediates. The compromised fractional enrichment of these isotopologues upon treatment at later times (i.e., 3 h, 6 h) points to reduced carbon feeding *via* PDH (Supporting PDF). In line with our finding, Lynch *et al.* (Lynch et al., 2017) demonstrated upregulation of the pyruvate dehydrogenase kinase (PDHK4) and increased PDH phosphorylation in HCC70 cells upon PI3Kβ inhibition, indicative of reduced carbon flux.

**FIGURE 5:**
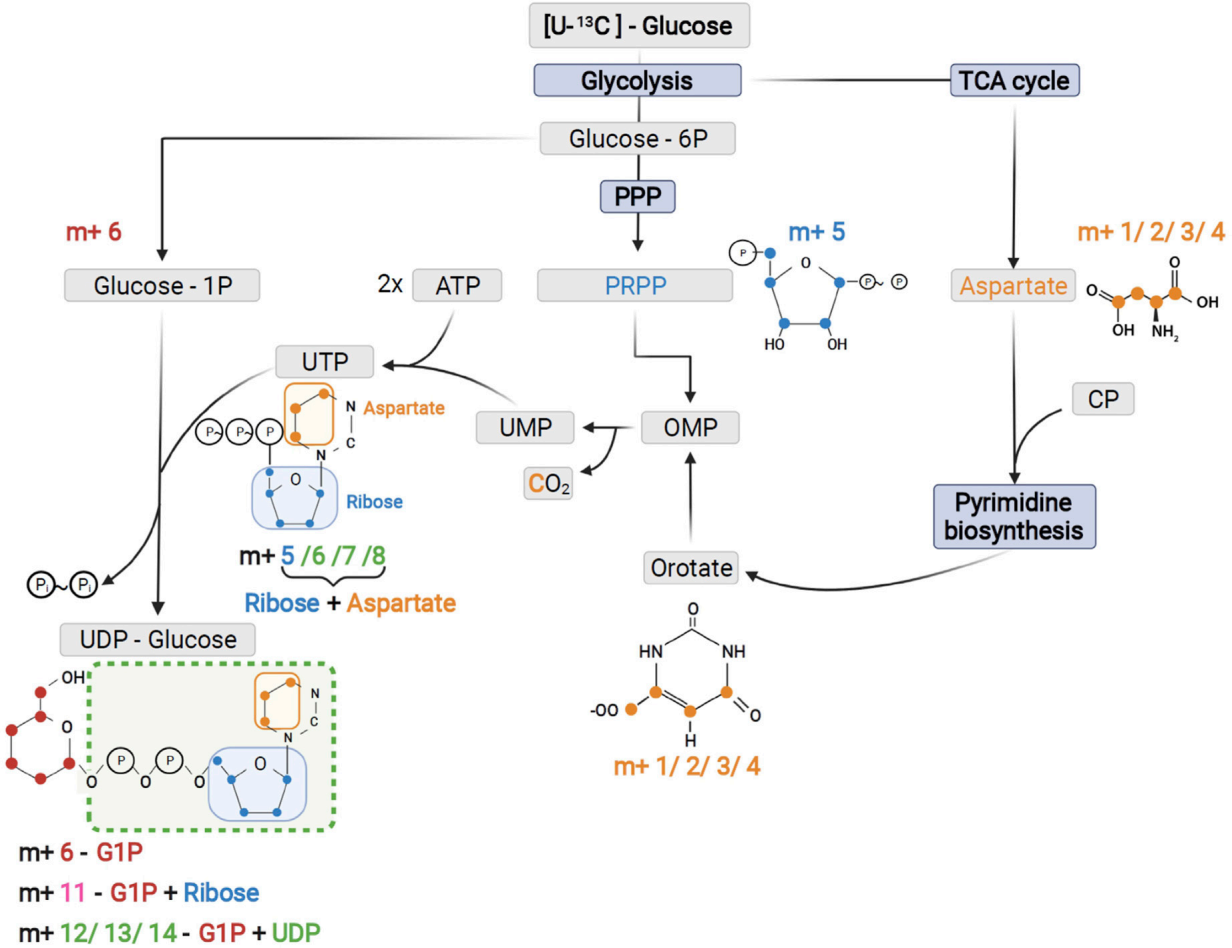
Schematic of UTP and UDP-Glucose biosynthesis with contribution of the different precursors to tracer incorporation from ^13^C_6_-glucose.

One of the most prominent effects of AZD8186 treatment was reduced tracer incorporation into adenylated purines (i.e., AMP, ADP and ATP) and uracil nucleotides (i.e., UDP and UTP) as demonstrated by fractional depletion of m+6/m+7 or m+7/m+8 isotopologues and a >2-fold higher presence of the m+0 isotopologues (Figure 4). For both metabolite classes this implies reduced incorporation of glucose carbon into nucleobases and ribose subunits. In accordance, tracer metabolism into m+3 and m+4 aspartate as essential building block of pyrimidines (Figure 5), was compromised (Figure 4). Likewise, at earlier time points, the most prominent fractional enrichment of the m+5 isotopologues, attributable to ^13^C_5_ ribose, was reduced significantly by treatment (Supporting PDF). This emphasizes the importance of the pentose phosphate pathway in MDA-MB-468 cells and suggests its inhibition as one potential mode of action of the PI3Kβ inhibitor.

**FIGURE 6:**
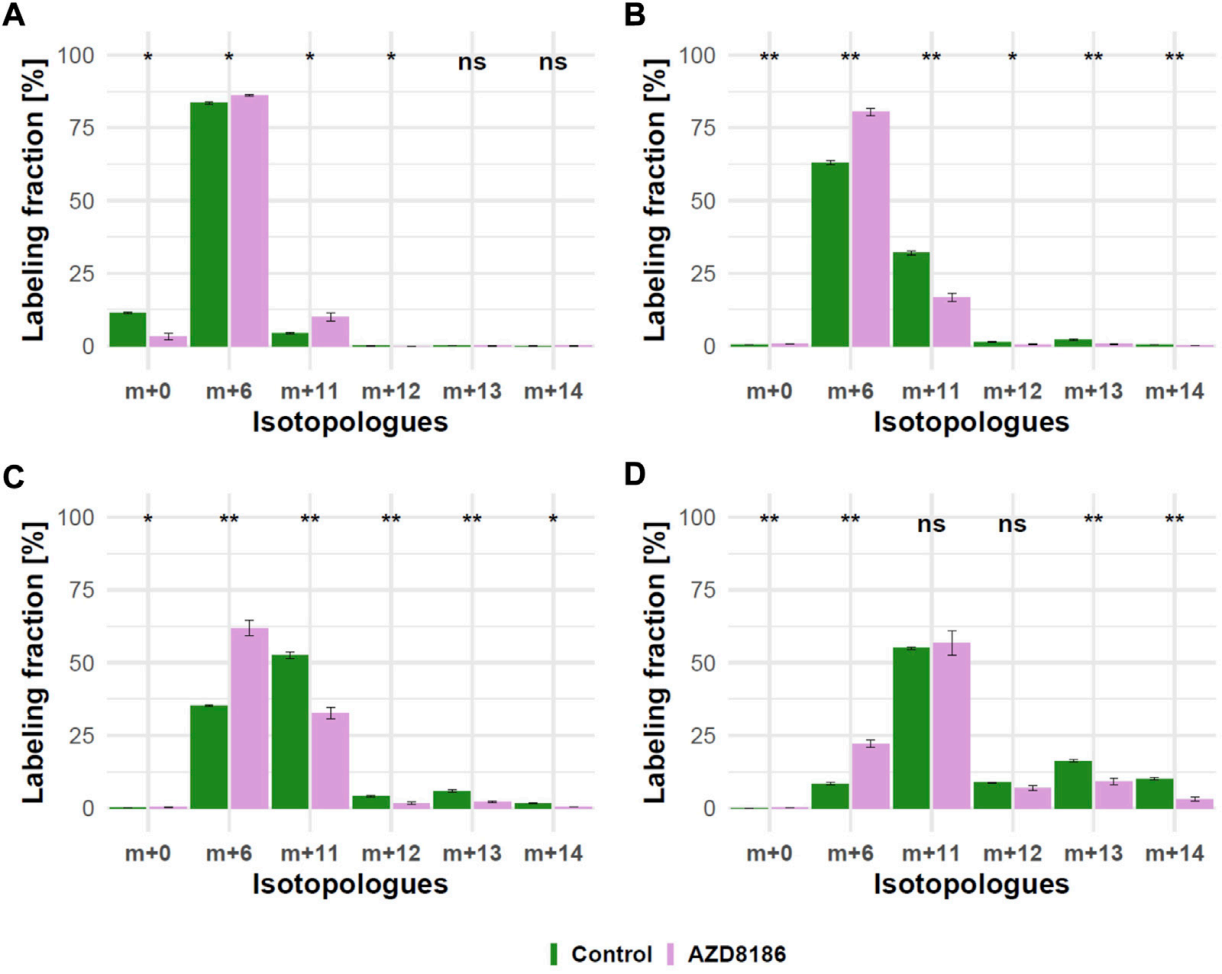
Time course of ^13^C tracer incorporation in UDP-Glucose. MDA-MB-468 cells were cultivated in RPMI with [U-^13^C] glucose and treated with 0.5 µM AZD8186 (purple) or vehicle (green). Bar charts ±standard deviation (n = 3 technical replicates) showing the fractional enrichment of ^13^C into UDP-Glucose at 1 h **(A)**, 3 h **(B)**, 6 h **(C)** and 24 h **(D)** sampling times. *p < 0.05; **p < 0.01 (adjusted *p*-value; Welch’s *t*-test).

As expected from metabolic alterations observed within glycolysis and nucleotide biosynthesis, downstream metabolites including the nucleotide sugars UDP-Glucose and UDP-GlcNAc showed corresponding changes. Both metabolites contain moieties from glycolytic intermediates (G1P in UDP-Glucose, G6P in UDP-GlcNAc) and pyrimidine nucleotides and carbon contribution from TCA cycle *via* aspartate. The biosynthesis of UTP and UDP-Glucose is schematically displayed in Figure 5 to demonstrate [^13^C_6_] glucose derived carbon incorporation into different molecular subunits. These comprise the m+6 isotopologue derived from G1P and the m+11 isotopologue reflecting G1P + Ribose (derived from PPP). The latter combines with orotate originating from aspartate to produce UTP with isotopologues m+5, m+6, m+7 and m+8. Within the final product UDP-Glucose, the UTP-derived labels are reflected as the m+12, m+13 and m+14 isotopologues. Drug-induced alterations in these pathways were observable as elevated tracer levels in the isotopologues m+5, 6, 8 and 11 for UDP-GlcNAc (Figure 4) and m+6 for UDP-Glucose (Figures 4, 6). Despite an increased label incorporation into these isotopologues, UDP-GlcNAc and UDP-Glucose both displayed diminished labeling in isotopologues generated by multiple pathway contributions (>m+11) indicating the reduced glycolytic ^13^C-flux in the presence of PI3Kβ inhibition (Figures 4, 6).

Intermediates from the mevalonate, glutathione and amino acid metabolism were also identified using the untargeted approach. In those cases, reduced relative fraction of higher isotopologues is caused by diminished ^13^C-flux into the TCA cycle with subsequent decreased labeling of the respective precursors for these metabolites, namely acetyl-CoA, glutamate or aspartate (Figure 4). The sialic acids CMP-NANA and NANA are linked to the UDP-GlcNAc metabolism and reduced tracer incorporation in higher isotopologues largely followed the patterns of the UDP-hexoses (Figure 4).

## 4 Discussion

Untargeted metabolomic profiling has become a valuable tool for the identification of potential metabolic biomarkers in cancer (Struck-Lewicka et al., 2020; Kowalczyk et al., 2021) or the assessment of the response to anti-tumor therapy (Han et al., 2021; Ruan et al., 2022). In order to investigate dynamic pathway alterations in metabolic networks, untargeted stable isotope resolved metabolomics (SIRM) can be applied as a complementary tool (Huang et al., 2014) to evaluate the cellular fate of precursor metabolites and thereby enabling deeper insight into dysregulation of cancer metabolism and individual drug response phenotypes.

Here, we present the establishment, validation and pharmacological application of a high-resolution LC/MS based workflow for the analysis of isotopic tracing experiments in cell culture using untargeted discovery of labeled metabolites followed by targeted extraction of isotopologues. In our proof of concept application, we investigated the metabolic consequences of PI3Kβ inhibition with the specific inhibitor AZD8186 in the PTEN-deficient human breast cancer line MDA-MB-468. PTEN deficiency leads to constitutive activation of the PI3K pathway, and inhibition of PI3Kβ in particular can increase tumor cell toxicity in certain mouse models. We have chosen [U-^13^C] glucose as a tracer due to its ability of label incorporation into many pathways along central carbon metabolism including glycolysis, TCA cycle and related branching pathways (e.g., *de novo* serine biosynthesis or acetyl CoA sources). We provide results on the following aspects: 1) advantage of untargeted data analysis to enhance pathway coverage (Huang et al., 2014; Llufrio et al., 2019), 2) demonstration of the complementary detection of isotope-enriched features relevant to drug response in comparison to conventional label-free untargeted metabolomics, 3) method validation with respect to inter- and inter-day repeatability, and 4) monitoring pathway activity changes upon PI3Kβ inhibition.

Using the X^13^CMS extraction routine as discovery and filtering tool for non-targeted identification of isotope-enriched metabolites produced candidate features that were assessed for structural assignment. Currently, besides 19 metabolites that could be attributed to pre-defined pathways, 11 additional compounds were identified to the highest Metabolomics Standard Initiative (MSI) level 1 (Salek et al., 2013) and integrated into the targeted feature extraction workflow for manual curation of the data thus increasing the accuracy of isotopologue quantitation. Most important, the knowledge of sum formulas of the newly identified metabolites enables the correction for natural isotopic abundance (Heinrich et al., 2018; Nilsson, 2020) thus overcoming a current limitation of the X^13^CMS workflow (Llufrio et al., 2019). Albeit beyond the scope of our study, annotation of additional features can be achieved by retrospective examination of high-resolution QTOF-MS data for a continuous integration of novel identified metabolites into the targeted data analysis workflow. Likewise, the available LC/MS data can be used for the extraction of isotopic data by other tools than X^13^CMS in order to expand the list of potential markers of drug response that, after their identification, can be implemented in the targeted data analysis routine. These tools may comprise geoRge (Capellades et al., 2016) or novel approaches that perform parameter optimization throughout the data processing workflow (Butin et al., 2022), thereby maximizing the yield of valuable isotopic data.

In our case, integration of the 11 newly identified metabolites into the existing targeted workflow resulted in an expansion of pathway coverage. Notably, all these metabolites have been associated with cancer metabolism and therapy, e.g., pyrimidine metabolism (Wang et al., 2021), hexosamine pathway (Ferrer et al., 2016; Lam et al., 2021), glutathione (Kennedy et al., 2020), sialic acids (Pearce and Läubli, 2016) and UDP-Glucose (Wang et al., 2019). More specifically, they all belong to key metabolic processes (i.e., glucose metabolism, biosynthesis of macromolecules, and maintenance of redox balance) that are activated downstream of the PI3K-AKT signaling network (Hoxhaj and Manning, 2020). Hence, our findings imply that specific inhibition of PI3Kβ has metabolic consequences similar to changes that occur within canonical PI3K signaling. Therefore, considering these additional pathways represents an important add-on and may guide biomarker and combination strategies for this class of agents in the future.

The complementary nature of significantly changed features found by the X^13^CMS routine compared to statistically altered features revealed upon data pre-processing in unlabeled samples with XCMS is in accordance with the literature (Huang et al., 2014). This complementarity was further confirmed by results obtained with vendor-specific profinder pre-processing whose outcome reflected the vast majority (19 out of 20) of significantly altered features retrieved by XCMS. As previously shown (Huang et al., 2014) this comparison demonstrates that different biological information can be obtained from the datasets. While the labeling approach provides results as a function of alterations in tracer incorporation, untargeted analysis in unlabeled samples discovers metabolites whose levels significantly change upon drug treatment. We would like to emphasize that comparison between labeled and unlabeled untargeted metabolomics as done here and previously by (Huang et al., 2014) allows the detection of significantly altered features obtained after data preprocessing with different workflows in labeled and unlabeled replicate samples derived from the very same cell culture experiment (i.e. similar to a “paired” experiment). Nevertheless, as for the X^13^CMS routine quality control (QC) samples are not designated, QC assurance criteria as commonly applied in label-free untargeted metabolomics experiments (i.e., CV filtering or signal stability assessment based on pooled QC samples) could not be applied. This is a limitation of our study, which would have necessitated an independent cell culture experiment tailored to typical QC assurance procedures of a conventional non-targeted metabolomics experiment. This however, was not the main scope of our work that aimed at the establishment of a workflow for untargeted SIRM.

Quality control and method validation is still a challenge in conventional LC-MS non-targeted metabolomics (Begou et al., 2018). In this regard, making use of tracer technology can be beneficial as we have demonstrated by high repeatability for technical replicates (intra-day) and between independent experiments (inter-day). While on the one hand, careful visual inspection and curation of peak area integration during targeted data pre-processing is one factor that contributes to high precision within batches, internal normalization of mass isotopomer distribution vectors has a beneficial effect as well. Thus, we demonstrated robust and repeatable sample preparation and analysis between different batches (inter-day) which may minimize the need for inter-batch corrections in large-scale studies. Altogether, to the best of our knowledge we have demonstrated here for the first time in cell culture that targeted SIRM provides a highly reproducible readout that will be a valuable contribution to already established validation schemes for tracer experiments (Heuillet et al., 2018; Schwaiger-Haber et al., 2019).

The proof of concept pharmacological treatment with AZD8186 correctly identified reduced carbon flux through glycolysis and *de novo* purine nucleotide synthesis in line with previous results. A decreased glycolytic rate in HCC70 and LNCAP cells upon PI3Kβ inhibition has been demonstrated by Seahorse flux analysis (Lynch et al., 2017). With semiquantitative metabolomic profiling the authors also revealed markedly depleted levels in the deoxyribonucleotide triphosphate (dNTP) pool in both cell lines. Our SIRM experiments in addition uncovered that AZD8186 lowers carbon incorporation into purine and pyrimidine ribonucleotides showing that the dNTP pool depletion results from reduced biosynthesis. Another notable observation was a significantly reduced incorporation of glucose carbon into intracellular lactate which to the best of our knowledge has not been demonstrated before for this isoform-specific inhibitor. Altogether, these metabolic changes suggest that PI3Kβ inhibition has a beneficial action in that it lowers the anaerobic conversion from glucose to lactate. Given that Lynch *et al.* observed a decrease in the extracellular acidification in the presence of the same concentration (0.5 µM) of AZD8186, it is tempting to speculate that reduced intracellular lactate production is a significant contributor to de-acidification of the extracellular space (e.g. possibly by reduced lactate secretion). Further, while Lynch *et al.* deduced a potential reduced carbon flux into TCA cycle from bioenergetic measurement (i.e. *via* reduced oxygen consumption) and PDHK4 expression without analyzing related metabolites, our results show that indeed carbon flow into these metabolites is compromised. This in turn supports a potential impact of the inhibitor on mitochondrial metabolism. Here, novel protocols for determining compartment-specific metabolic fluxes (Nonnenmacher et al., 2019) either alone or complemented by data on mitochondrial oxygen consumption (Jones et al., 2021) will be a powerful combination to broaden the understanding of how mitochondrial metabolism is affected upon PI3Kβ inhibition. Our observations of a reduced glycolytic rate confirms that PI3K/Akt signaling is a major regulator of important cellular processes including glucose homeostasis (Schultze et al., 2012). Therefore, targeting PI3K/Akt pathways may have consequences on whole body energy metabolism including treatment of complex metabolic disease. For example, altering PI3K/Akt signaling in pancreatic β-cells is considered as emerging therapeutic strategy for type 1 diabetes (Camaya et al., 2022). Experiments using transgenic murine models have provided evidence that PI3K/Akt signaling is a critical determinant of β-cell mass and function whose impairment is supposed to be an early feature of pathogenesis in type 1 and type 2 diabetes (Chen et al., 2017). With respect to PI3Kβ a potential mechanism of the regulation of insulin secretion has been described as kinase inhibition affects the differentiation of human stem cells into beta cells, thereby suggesting a role for PI3Kβ in embryonic development of the pancreas (Mao et al., 2017). Isoform-specific targeting is therefore an attractive strategy in the clinic to develop inhibitors with optimal therapeutic index to control for altered blood glucose homeostasis, which can be a potential side effect due to the complex involvement of PI3K isoforms in insulin action (Molinaro et al., 2019).

Stable isotope tracing is a powerful method for interrogating metabolic enzyme activities. We have demonstrated exemplarily that the route of pyruvate entry into mitochondrial metabolism can be deduced from the m+2 *vs.* m+3 isotopologue profiles of citrate and other TCA cycle intermediates (Buescher et al., 2015; Ma et al., 2019). Irrespective of AZD8186 exposure, MDA-MB-468 cells showed a high PDH activity signature as indicated by the more pronounced basal m+2 isotopologues compared to m+3. The reduced incorporation into m+2 citrate upon treatment is in line with previously reported inhibition of PDH activity as evidenced by increased PDH phosphorylation (Lynch et al., 2017).

We must emphasize that interpretation of TCA cycle isotopologue profiles was carried out in the dynamic labeling phase which limits interpretation to the assessment of relative flux differences in metabolic pathways. The quantitative contribution of ^13^C glucose to TCA intermediates pool sizes would either require enrichment to isotopic steady state (Buescher et al., 2015; Lorkiewicz et al., 2019) or analysis by non-stationary metabolic flux analysis (Jazmin and Young, 2013). This is an often overlooked aspect and should be carefully considered if information of metabolite pool size may be inferred from dynamic labeling data. Non-steady state labeling was also observed in larger composite molecules like UDP GlcNAc and UDP-Glucose where the reduced carbon flux through glycolysis and TCA cycle was reflected in structural subunits (i.e., ribose, aspartate) that represent contributions of these pathways (Moseley et al., 2011; Nakajima et al., 2013). As an example, we have demonstrated different pathway contributions in the presence of AZD8186 for UDP-Glucose (Figures 5, 6). Here reduced incorporation into higher isotopologues (≥m+11) mirrors the reduced carbon incorporation into metabolites of glycolysis, TCA cycle and pyrimidine biosynthesis. Altogether, these observations provide a first hypothesis for a more refined investigation of these branching pathways and their potential contribution to the mode of AZD8186 action.

An important aspect of our newly established workflow is its general applicability. Albeit it was set up in 2D cell culture (Figure 1), it can be easily adapted to monitor the metabolic fate of isotopic tracers in other *in vitro* systems, including patient-derived 3D models (Fan et al., 2018) or to probe tumor (Faubert et al., 2021) or cell metabolism (Fernández-García et al., 2020) *in vivo*. Notably, a recent comparison of metabolic flux analysis demonstrated that glucose metabolism in 3D spheroids differs significantly from 2D cultures (Tidwell et al., 2022), hence emphasizing the need to make use of 3D models for investigating cancer metabolism and drug response in cell culture. In this regard, *in vivo* profiling of tumor metabolic routes within the appropriate tissue environment will be key for interrogating drug action in human cancers (Lane et al., 2016) and to identify relevant alterations that contribute to cancer onset and progression (Grima-Reyes et al., 2021). While the adaption capability to different biological systems is one aspect of our workflow, usage of different tracers can also be readily implemented to gain complementary information (e.g. on pyrimidine biosynthesis with [U-^15^N] glutamine (Jang et al., 2018)) or to consider other pathways (e.g. pentose phosphate pathway with [1,2–^13^C]glucose) that are relevant for cancer metabolic reprogramming (Wang et al., 2016; Cossu et al., 2020). Lastly, implementation of novel isotopic tracing metabolomics technologies that are capable to increase coverage of isotopic label detection (Wang et al., 2022), enable metabolism assessment in cell subpopulations (Roci et al., 2016) or large-scale profiling via “deep labeling” (Grankvist et al., 2020) can further support discovery of yet unknown markers of cancer and drug response.

## 5 Conclusion

In this work, the successful establishment of a targeted and non-targeted SIRM workflow for the assessment of metabolic pathway alterations is presented. With respect to assay performance, the entire workflow was validated and demonstrated high reproducibility for technical replicates and between experiments. In a proof of concept application, specific inhibition of the PI3Kβ isoform in the PTEN-deficient MDA-MB-468 breast cancer cell line confirmed significant alterations in central metabolism while additional changes in pyrimidine biosynthesis and hexosamine pathways were identified. Discovery of additional metabolites, that were found changed as a function of drug response, were in turn revealed by applying the X^13^CMS routine for global tracking of isotopic labels in untargeted metabolomics. This kind of unbiased data analysis strategy demonstrates how specific changes in drug-induced pathway activities can be uncovered without preliminary assumptions. Thereby new hypothesis about potentially targetable pathways may be revealed which in turn paves the way for novel treatment concepts in cancer precision medicine.

## Data Availability

The original contributions presented in the study are included in the article/Supplementary Material; further inquiries can be directed to the corresponding author.

## Glossary

µM: micromolar
2-PG: 2-phosphoglycerate
3H3MG: 3-hydroxy-3-methylglutarate
3-PG: 3-phosphoglycerate
ACN: acetonitrile
ADP: adenosine diphosphate
AmAc: ammonium acetate
AMP: adenosine monophosphate
ATP: adenosine triphosphate
ATCC: American Type Culture Collection
CMP-NANA: cytidine monophosphate N-acetylneuraminate
CV: coefficient of variation
DMSO: dimethyl sulfoxide
EIC: extracted ion chromatogram
ESI: electrospray ionization
F16BP: fructose 1,6-bisphosphate
F6P: fructose 6-phosphate
FBS: fetal bovine serum
FC: fold change
G1P: glucose 1-phosphate
G6P: glucose 6-phosphate
GSH: glutathione
GSSG: oxidized glutathione
h: hour
HILIC: hydrophilic interaction liquid chromatography
HPLC: high performance liquid chromatography
i.d.: inner diameter
LC-QTOF-MS: liquid chromatography quadrupole time-of-flight mass spectrometry
m/z: mass over charge
mDa: millidalton
mM: millimolar
MS: mass spectrometry
MSI: Metabolomics Standard Initiative
NAA: N-acetylaspartate
NANA: N-acetylneuraminate
PBS: phosphate buffered saline
PDHK: pyruvate dehydrogenase kinase
PEP: phosphoenolpyruvate
ppm: parts per million
PTEN: phosphatase and tension homolog
RSD: relative standard deviation
RT: retention time
SIRM: stable isotope-resolved metabolomics
TCA cycle: tricarboxylic acid cycle
TNBC: triple negative breast cancer
UDP: uridine diphosphate
UDP-GlcNAc: uridine diphosphate N-acetylglucosamine
UMP: uridine monophosphate
UTP: uridine triphosphate
