# Supplementary material for: Untargeted stable isotope-resolved metabolomics to assess the effect of PI3Kβ inhibition on metabolic pathway activities in a PTEN null breast cancer cell line
Source: Front Mol Biosci. 2022 Oct 14;9:1004602. doi: 10.3389/fmolb.2022.1004602 (PMC9614656; doi:10.3389/fmolb.2022.1004602)
Supplement: Supplementary file 1 [file DataSheet1.zip › Supplementary Figures.pdf]

Supplementary Figures

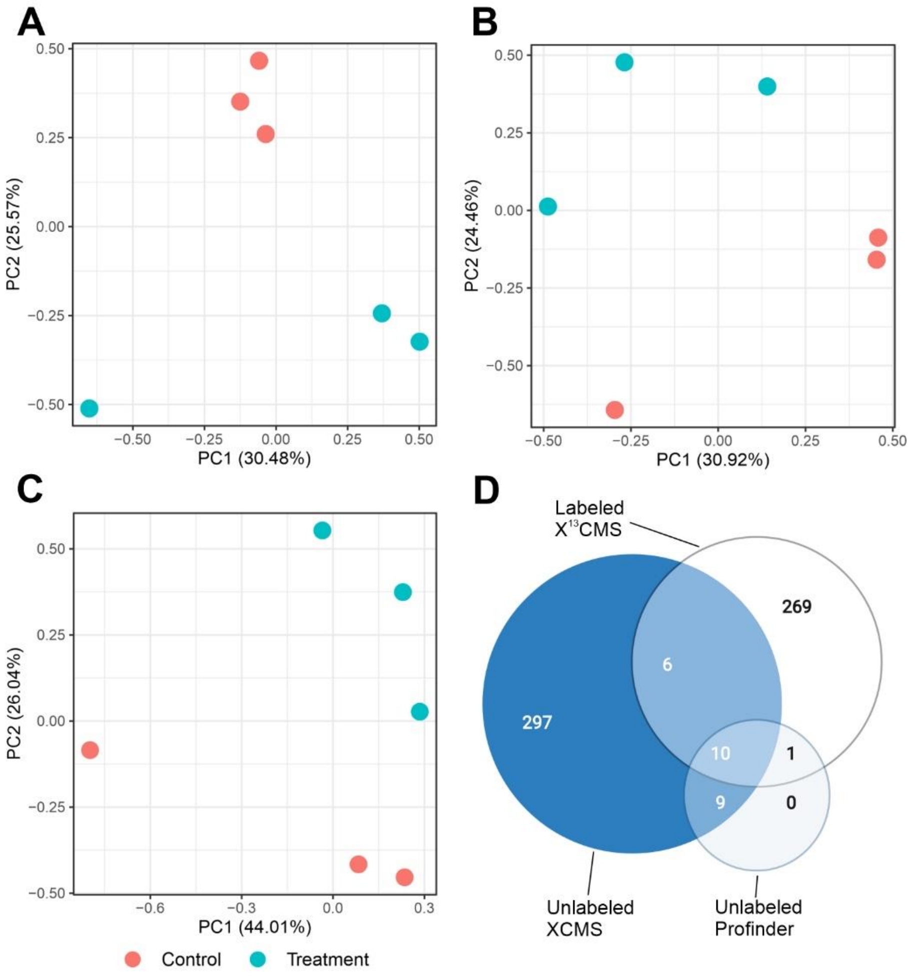

Supplementary Figure 1. Comparison of group discrimination between control (orange) and AZD8186 treated (cyan) samples based on metabolic features identified by untargeted SIRM with X^{13}CMS (**A**, 1.555 features) and those obtained in unlabeled samples upon pre-processing with XCMS (**B**, 12.391 features) and vendor-dependent Profinder (**C**, 490 features). Principal component analysis (PCA) was done with n = 3 technical replicates in unlabeled and labeled samples after 24h cultivation with 0.5 µM AZD8186. PCA from X^{13}CMS data was performed based on unique isotopologues present in both conditions (i.e. control and treatment groups). **D**, Comparison of significantly altered features related to AZD8186 treatment identified by untargeted SIRM ("Labeled X^{13}CMS") compared to results obtained upon data pre-processing with vendor-dependent Profinder and XCMS (i.e. miniDiffReport export) in unlabeled samples followed by statistical testing. (n = 3 technical replicates, unlabeled and labeled samples after 24h cultivation with 0.5 µM AZD8186. FC < 0.8, or FC > 1.2, Welch´s test, adjusted $p < 0.05$).

Supplementary Figures

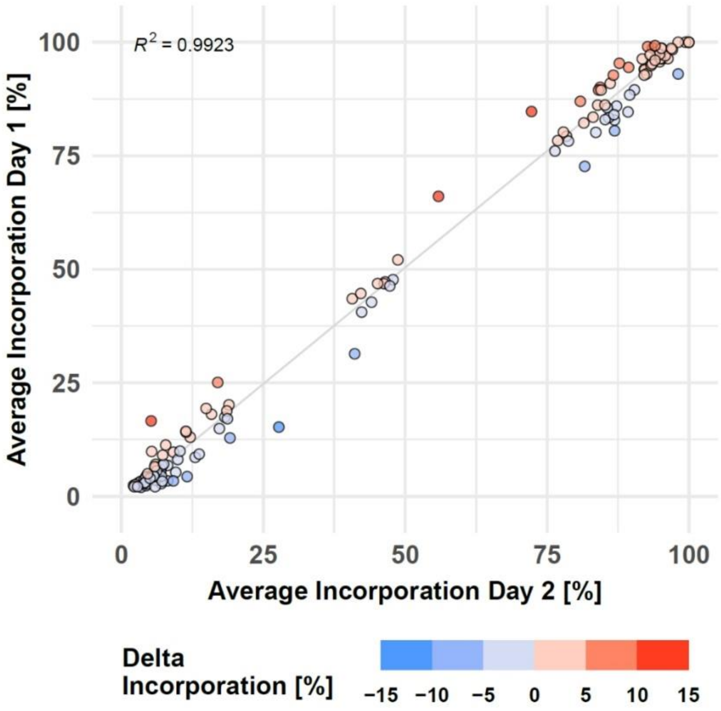

Supplementary Figure 2. SIRM robustness evaluation after data preprocessing with targeted library (Table S1). Inter-day reproducibility of SIRM experiments indicated as average tracer incorporation into 135 isotopologues on day 1 vs. day 2 (see Table S9, tracer incorporation >2% and <100%) at 1h sampling time point.
