# Supplementary material for: Untargeted stable isotope-resolved metabolomics to assess the effect of PI3Kβ inhibition on metabolic pathway activities in a PTEN null breast cancer cell line
Source: Front Mol Biosci. 2022 Oct 14;9:1004602. doi: 10.3389/fmolb.2022.1004602 (PMC9614656; doi:10.3389/fmolb.2022.1004602)
Supplement: Supplementary file 1 [file DataSheet1.zip › Supporting PDF.PDF]

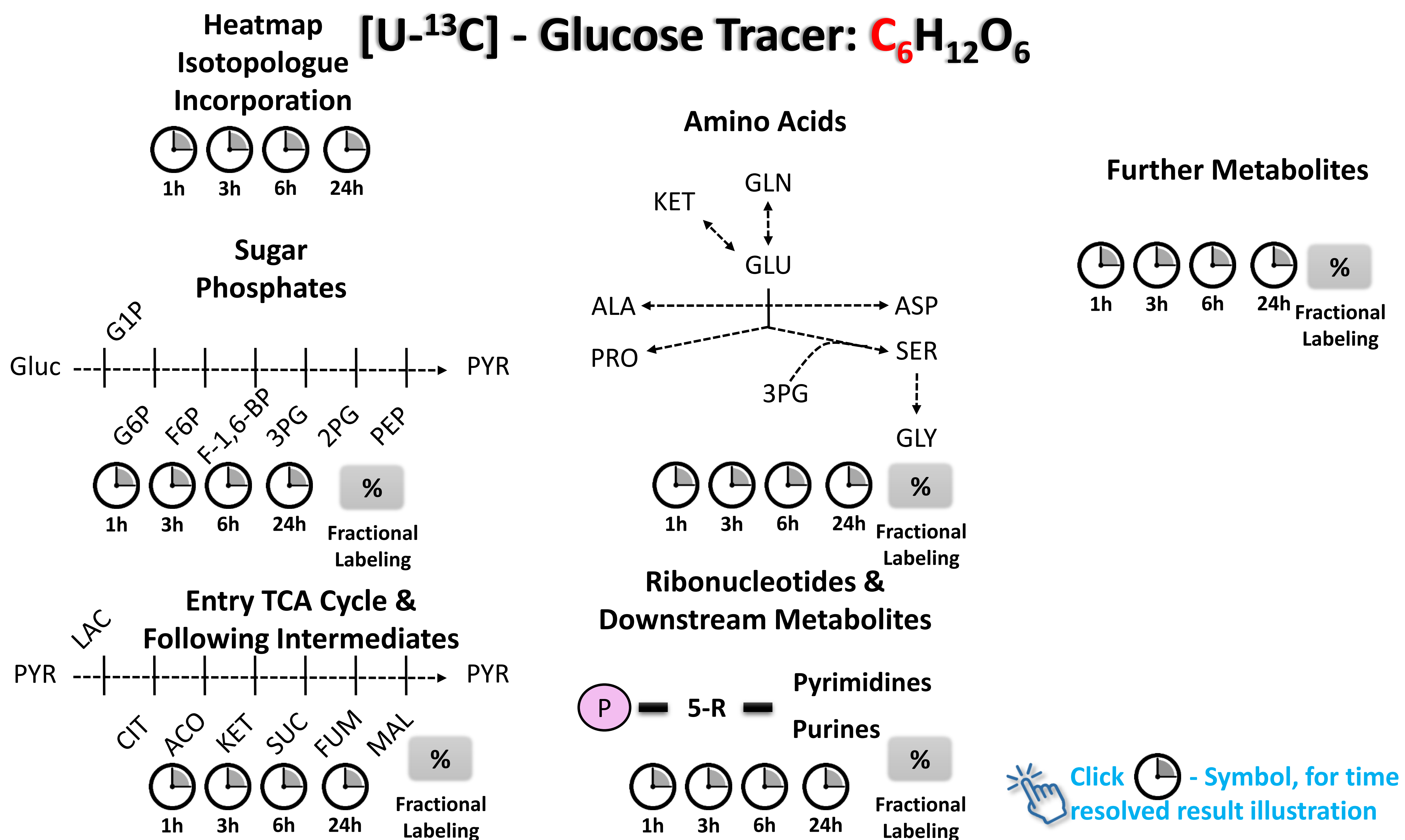
Heatmap
Isotopologue
Incorporation
1h 3h 6h 24h
[U-13C] - Glucose Tracer: C6H12O6
Sugar
Phosphates
Gluc
PYR
G1P
G6P
F6P
F-1,6-BP
3PG
2PG
PEP
1h 3h 6h 24h
%
Fractional
Labeling
Entry TCA Cycle &
Following Intermediates
PYR
PYR
LAC
CIT
ACO
KET
SUC
FUM
MAL
1h 3h 6h 24h
%
Fractional
Labeling
Amino Acids
GLN
KET
GLU
ALA
ASP
PRO
SER
3PG
GLY
1h 3h 6h 24h
%
Fractional
Labeling
Ribonucleotides &
Downstream Metabolites
P
5-R
Pyrimidines
Purines
1h 3h 6h 24h
%
Fractional
Labeling
Further Metabolites
1h 3h 6h 24h
%
Fractional
Labeling
Click - Symbol, for time
resolved result illustration

Fold Change Treatment vs. Control - Labeling Fraction Isotopologues

Sugar Phosphates

Entry TCA, Lactate Fermentation

TCA

Amino Acids

Ribonucleotides & Others

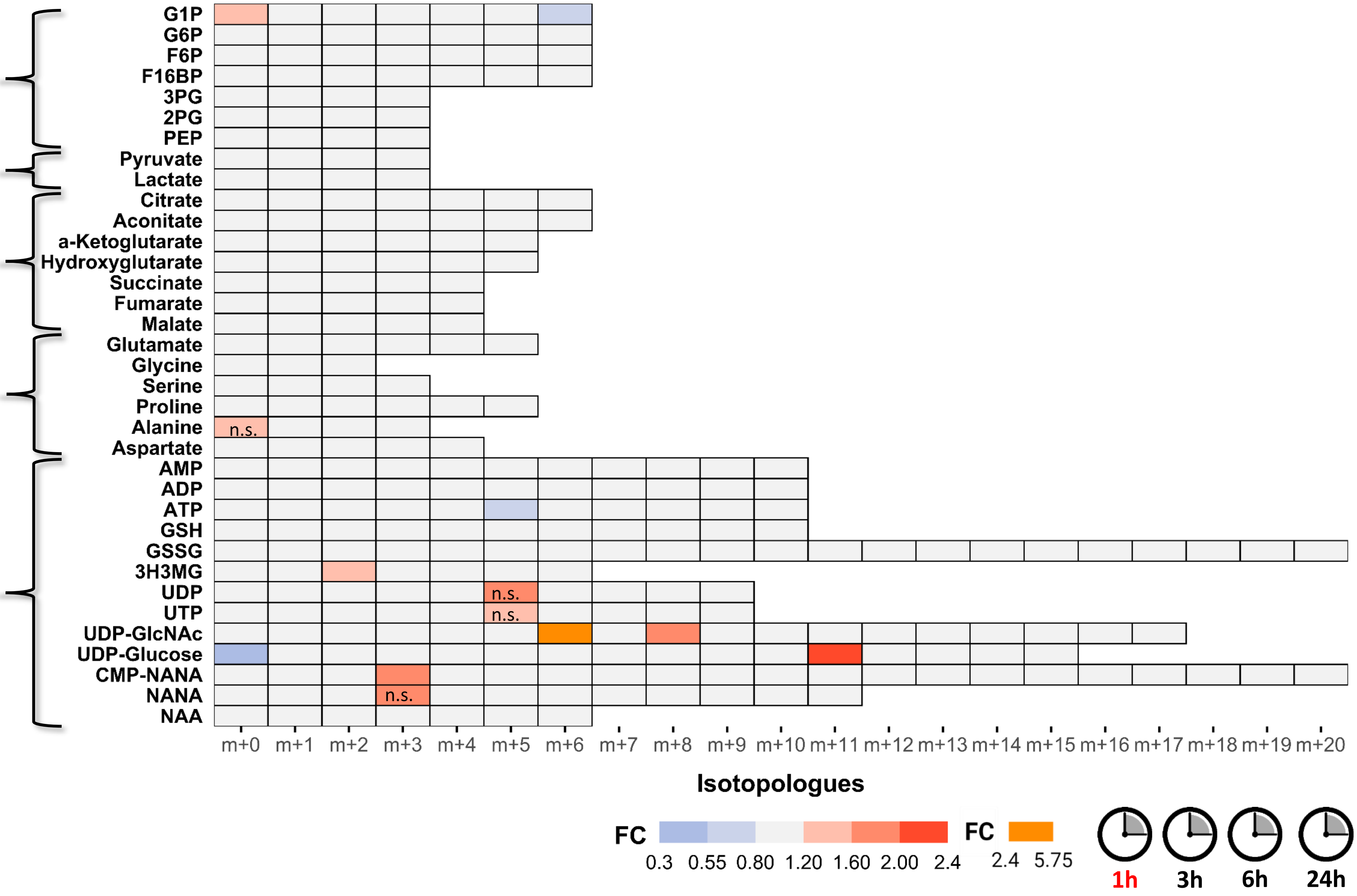

Fold Change Treatment vs. Control - Labeling Fraction Isotopologues

Sugar Phosphates

Entry TCA, Lactate Fermentation

TCA

Amino Acids

Ribonucleotides & Others

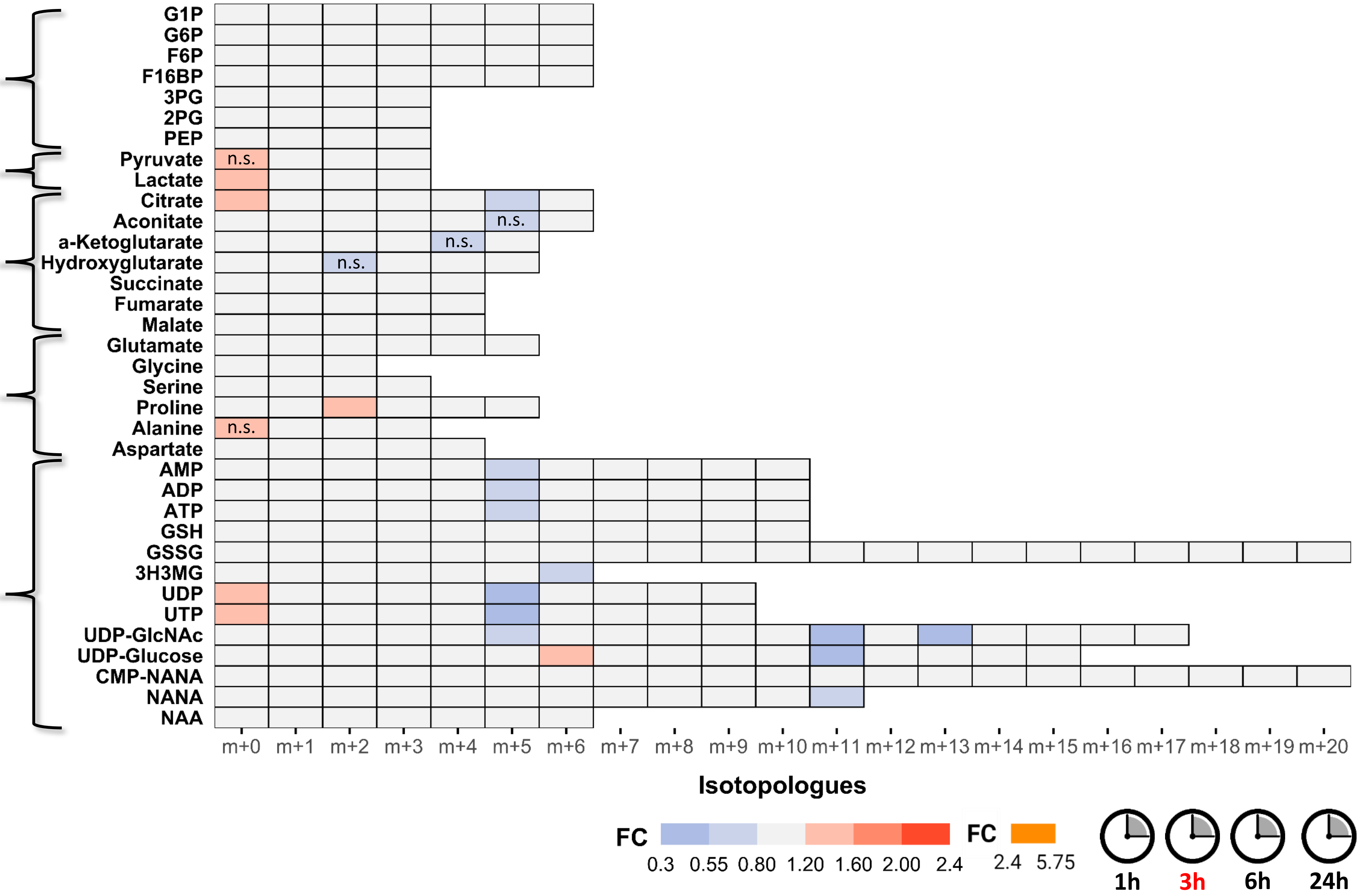

Fold Change Treatment vs. Control - Labeling Fraction Isotopologues

Sugar Phosphates

Entry TCA, Lactate Fermentation

TCA

Amino Acids

Ribonucleotides & Others

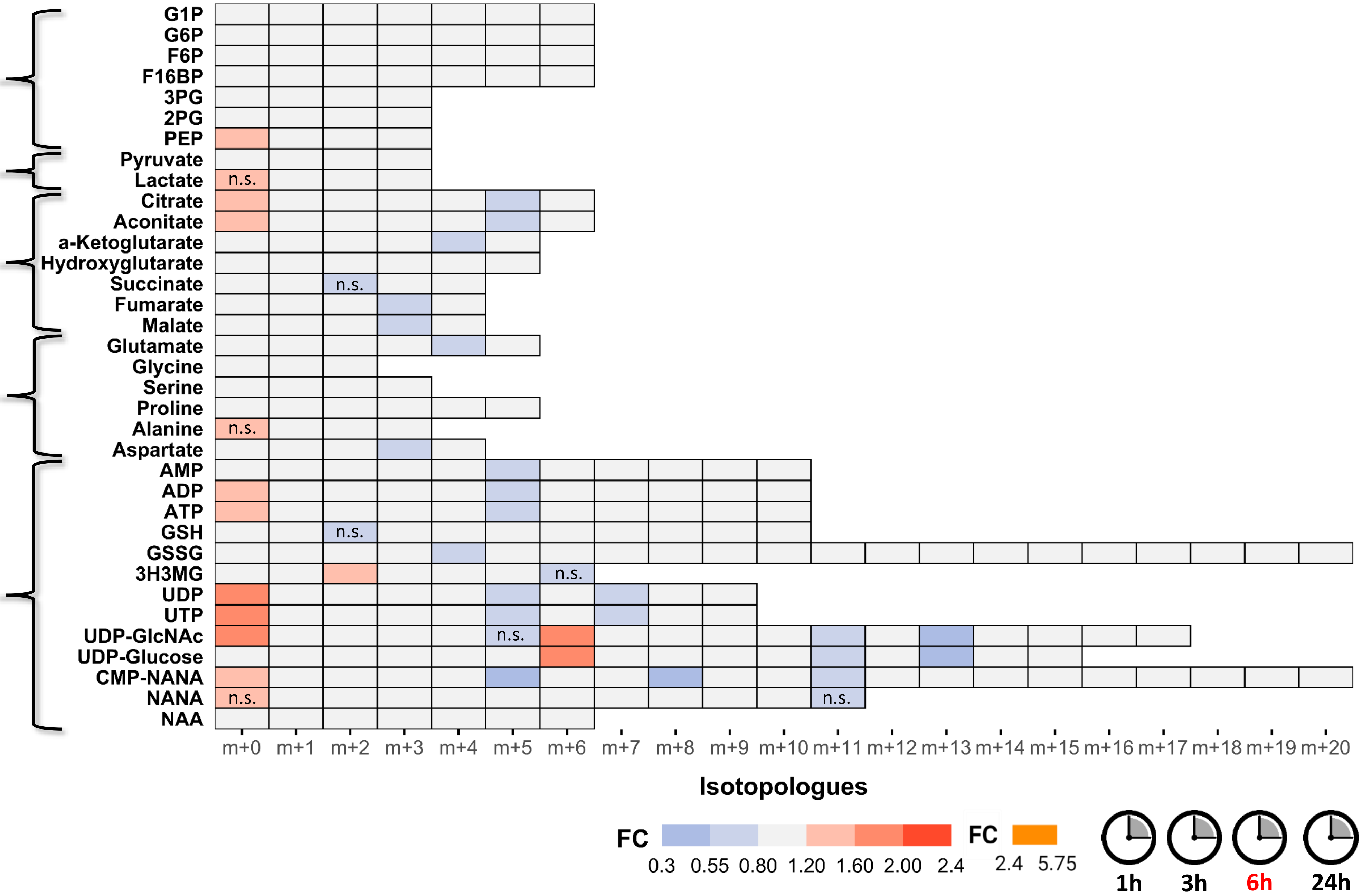

Fold Change Treatment vs. Control - Labeling Fraction Isotopologues

Sugar Phosphates

Entry TCA, Lactate Fermentation

TCA

Amino Acids

Ribonucleotides & Others

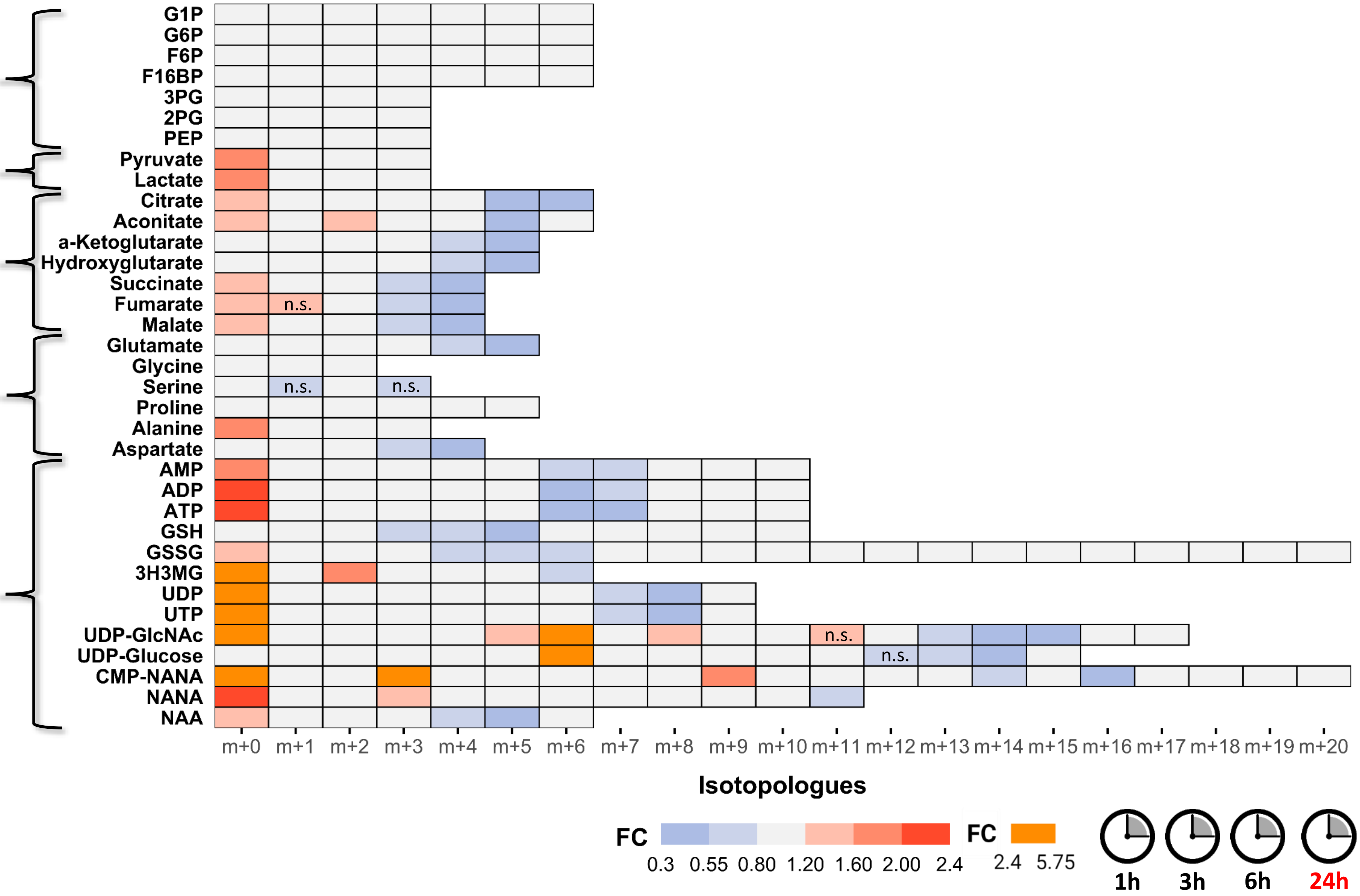

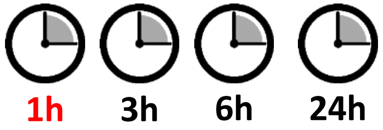

Sugar Phosphates

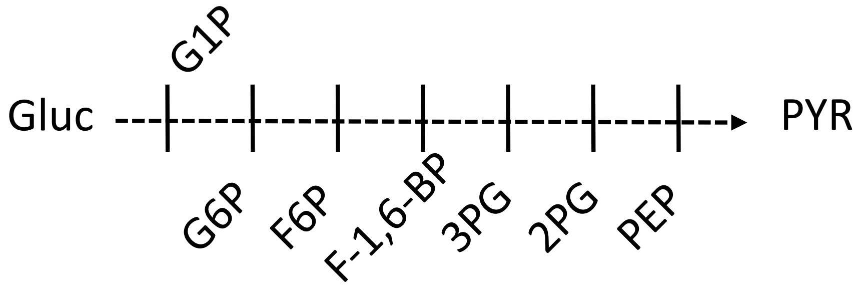

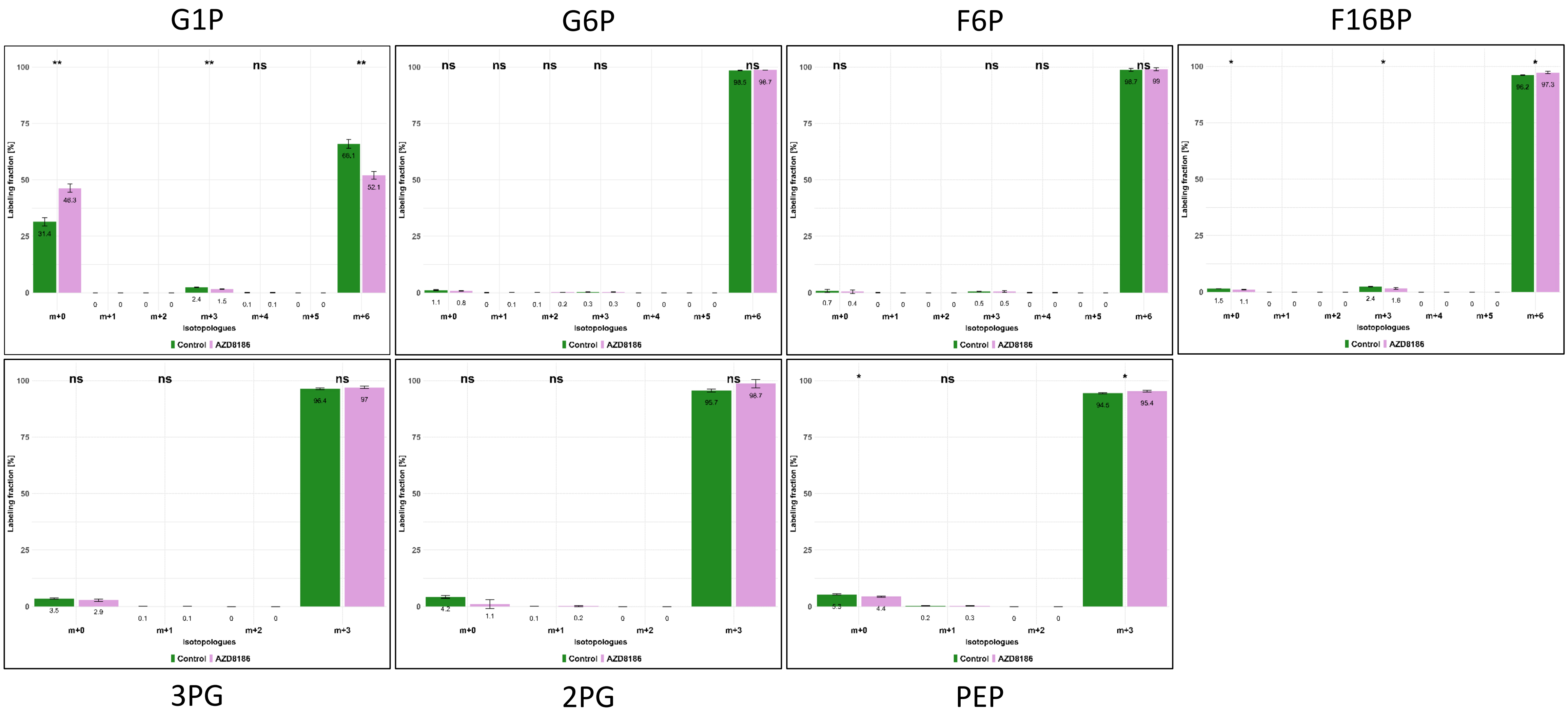

Bar charts ± standard deviation (n=3 technical replicates); * p< 0.05; ** p< 0.01; *** p<0.001 (adjusted p-value; Welch's t-test)

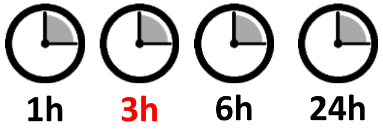

Sugar Phosphates

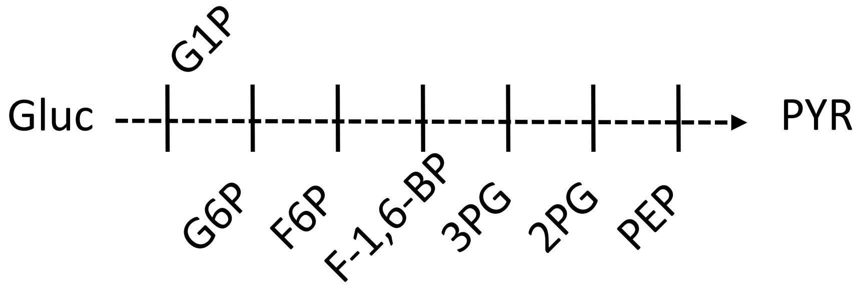

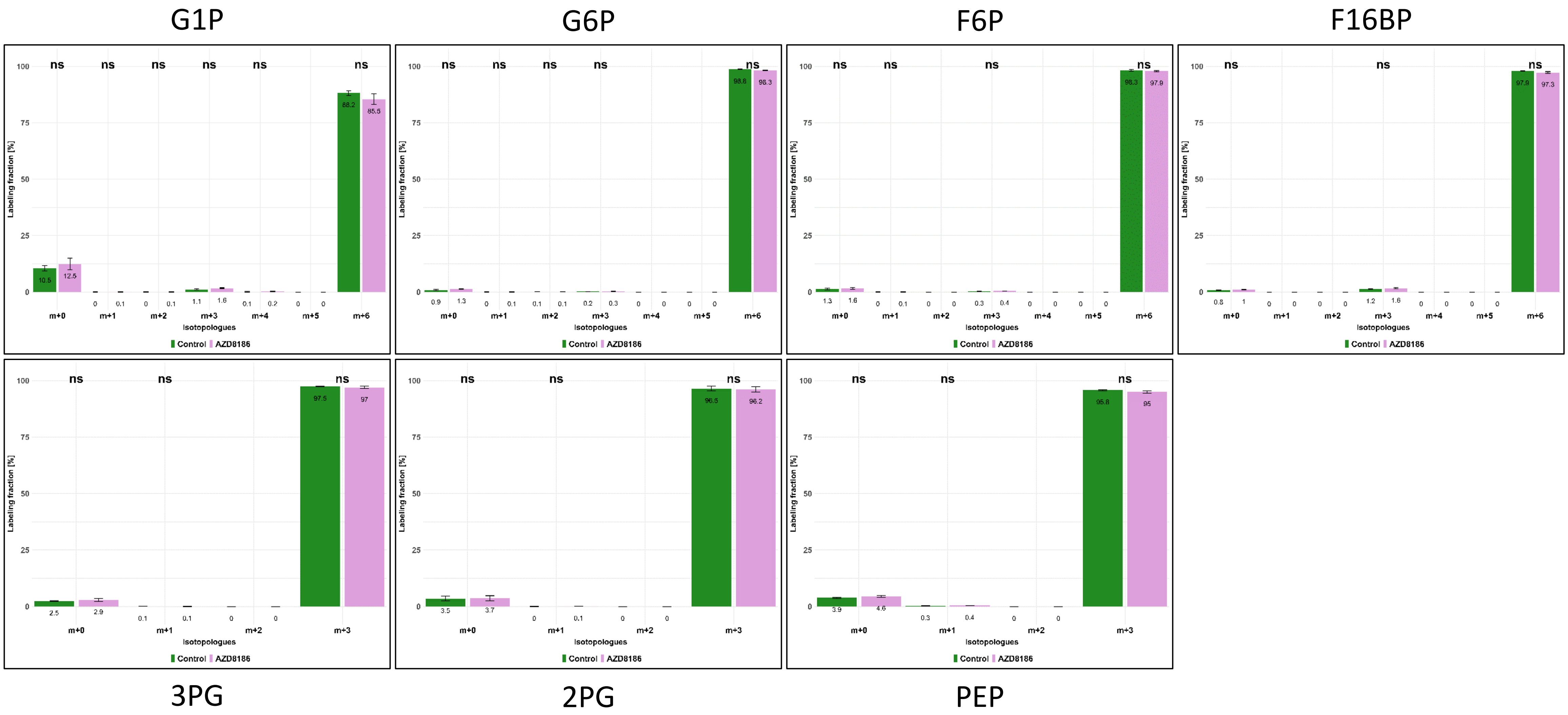

Bar charts ± standard deviation (n=3 technical replicates); * p< 0.05; ** p< 0.01; *** p<0.001 (adjusted p-value; Welch's t-test)

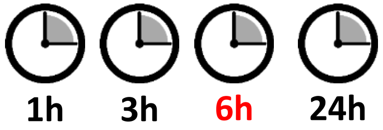

Sugar Phosphates

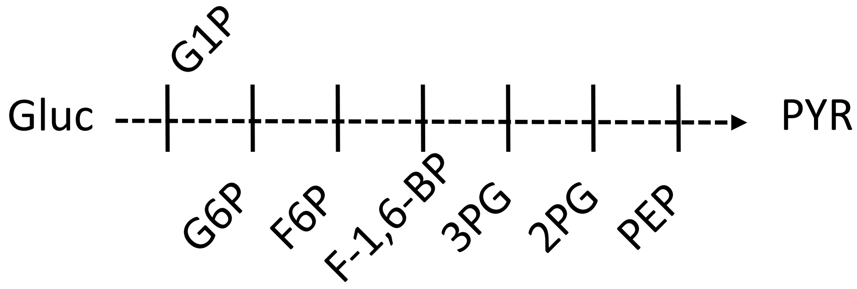

G1P

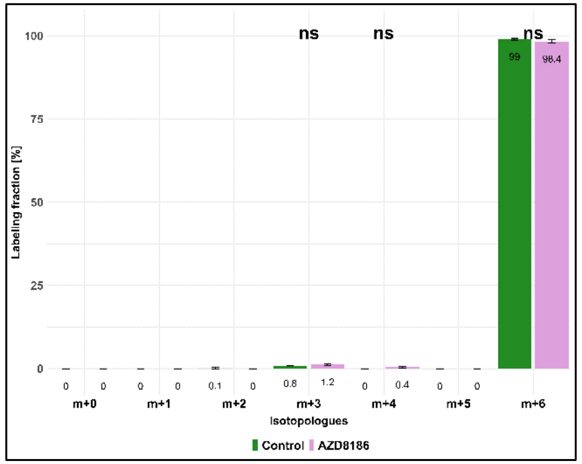

G6P

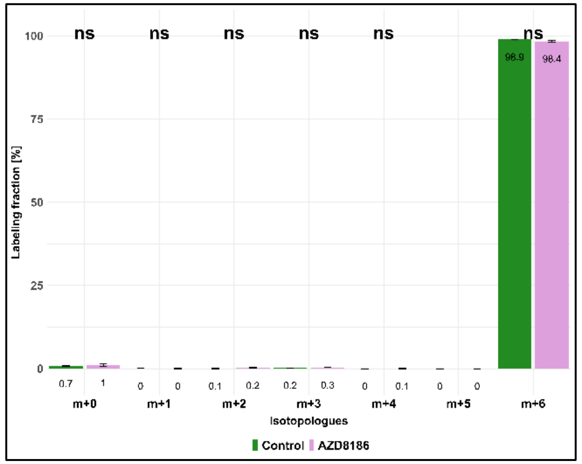

F6P

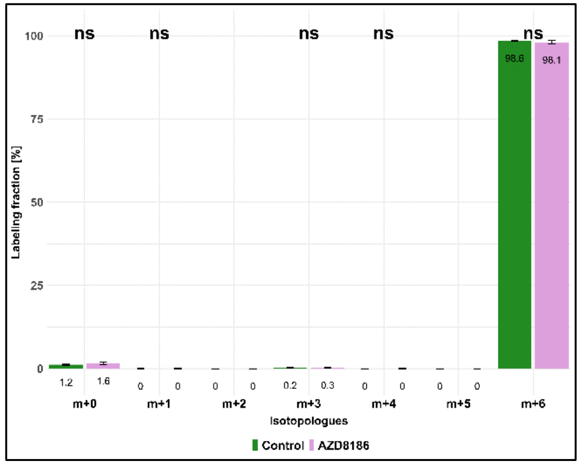

F16BP

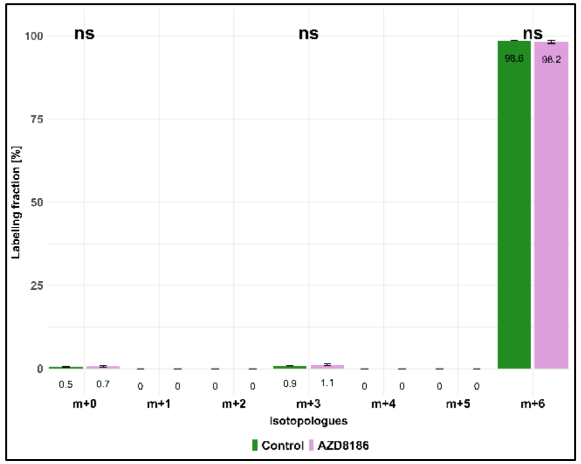

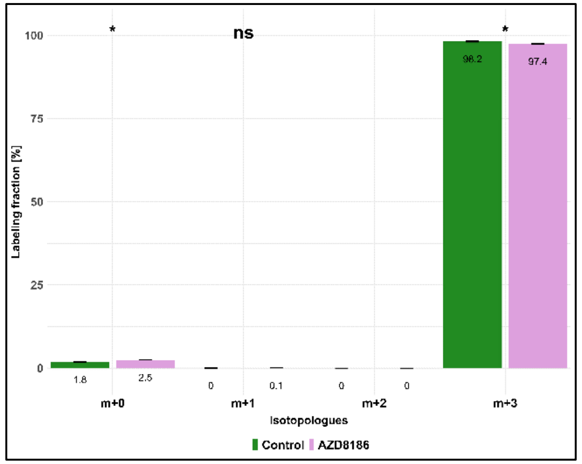

3PG

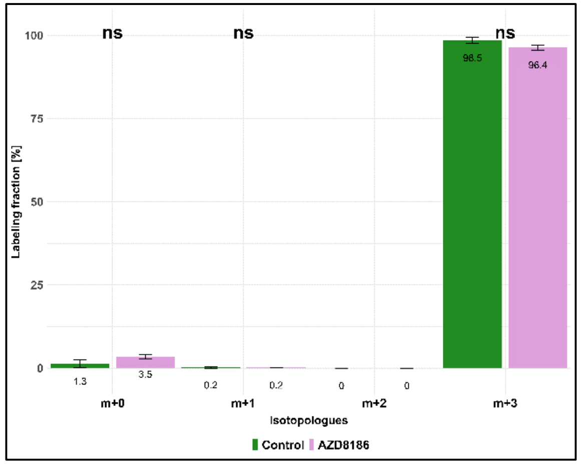

2PG

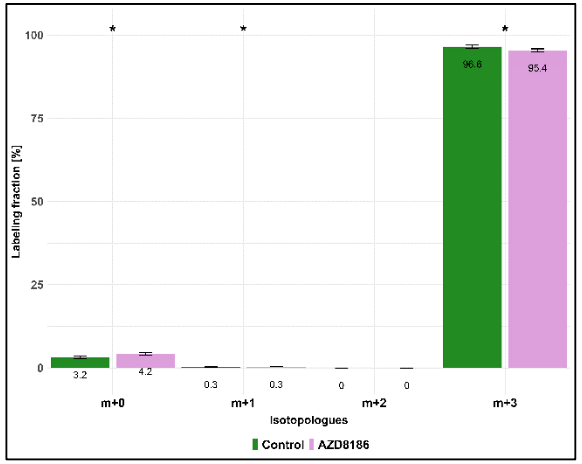

PEP

Bar charts ± standard deviation (n=3 technical replicates); * $p < 0.05$; ** $p < 0.01$; *** $p < 0.001$ (adjusted p-value; Welch's t-test)

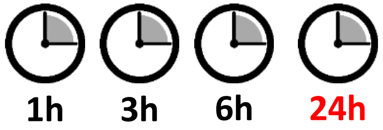

Sugar Phosphates

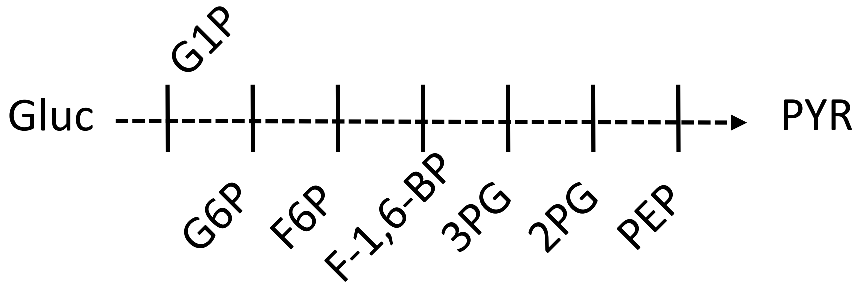

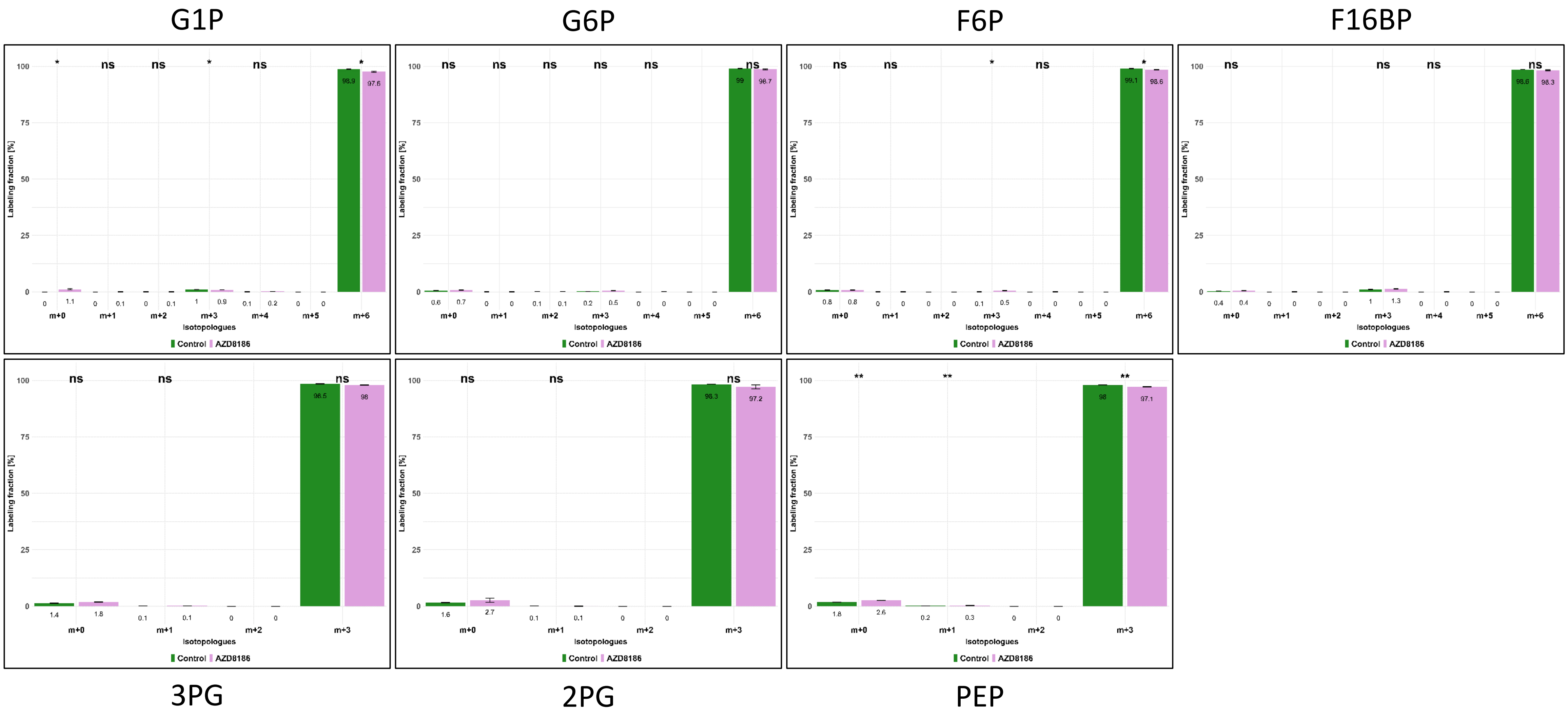

Bar charts ± standard deviation (n=3 technical replicates); * p< 0.05; ** p< 0.01; *** p<0.001 (adjusted p-value; Welch's t-test)

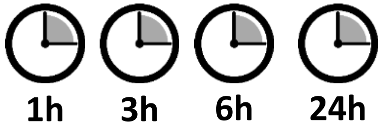

Sugar Phosphates

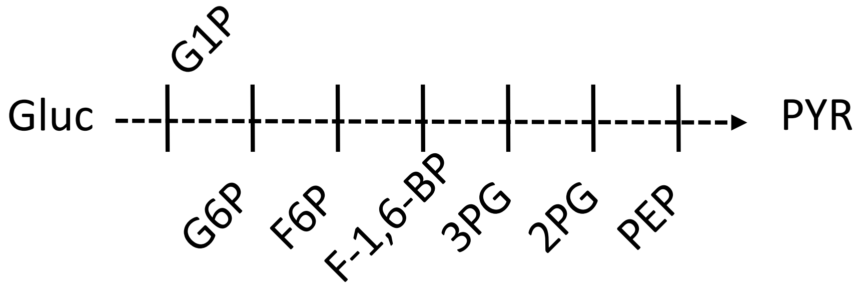

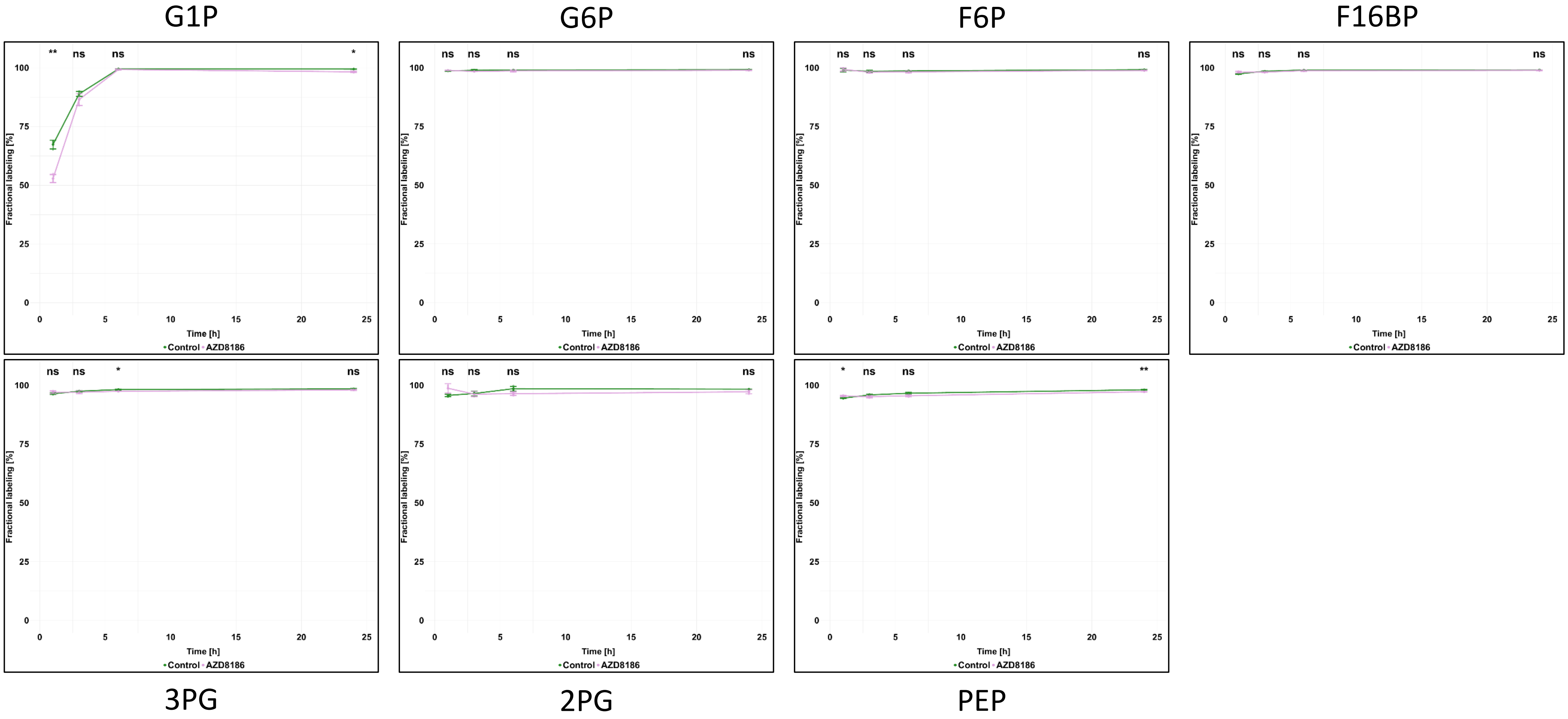

Line graphs ± standard deviation (n=3 technical replicates); * p< 0.05; ** p< 0.01; *** p<0.001 (adjusted p-value; Welch's t-test)

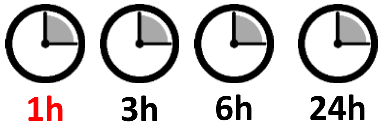

Transition Into TCA Cycle & Following Intermediates

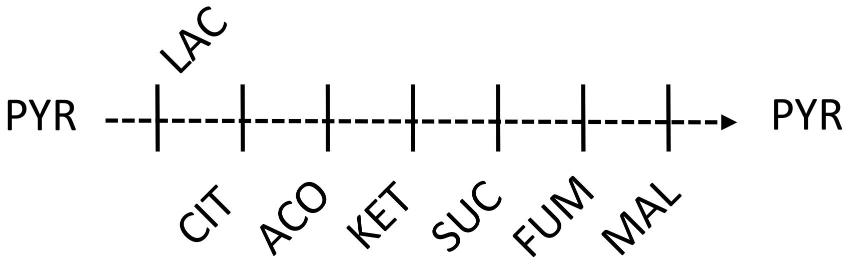

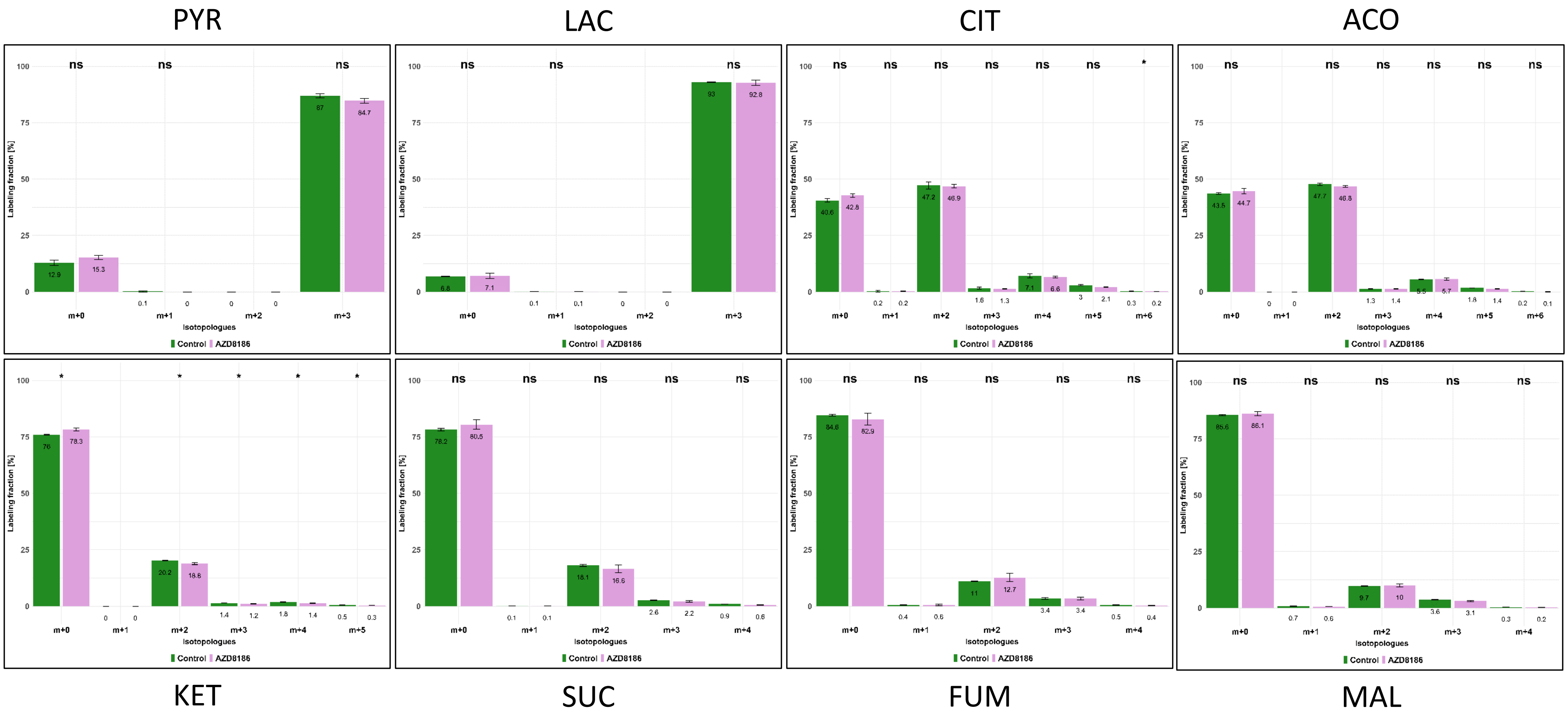

Bar charts ± standard deviation (n=3 technical replicates); * p< 0.05; ** p< 0.01; *** p<0.001 (adjusted p-value; Welch's t-test)

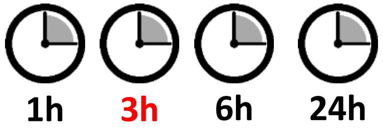

Transition Into TCA Cycle & Following Intermediates

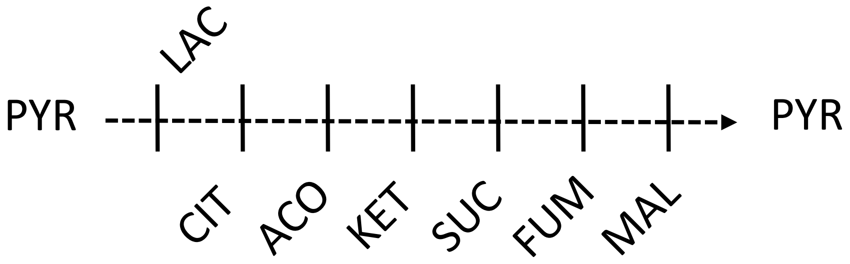

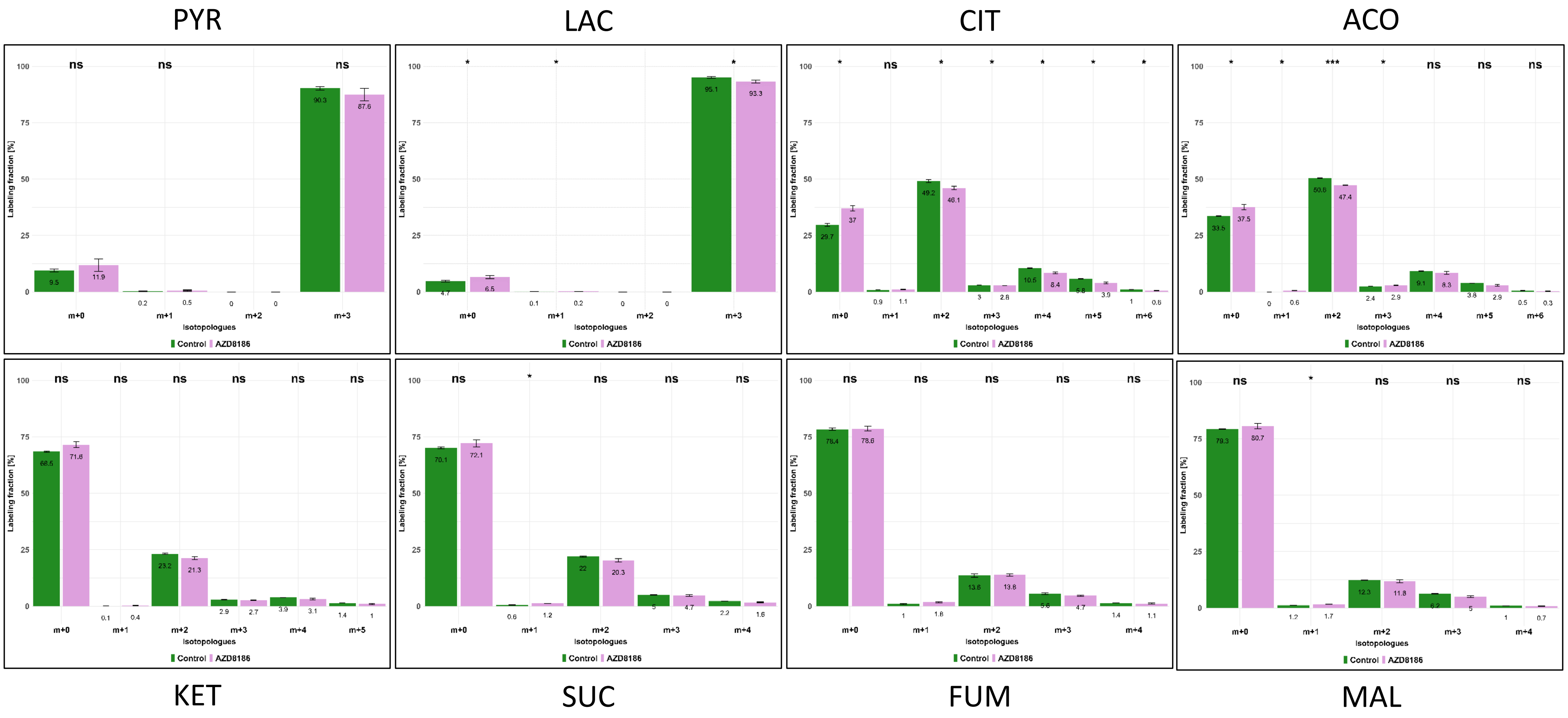

Bar charts ± standard deviation (n=3 technical replicates); * p< 0.05; ** p< 0.01; *** p<0.001 (adjusted p-value; Welch's t-test)

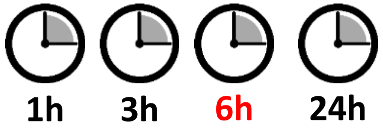

Transition Into TCA Cycle & Following Intermediates

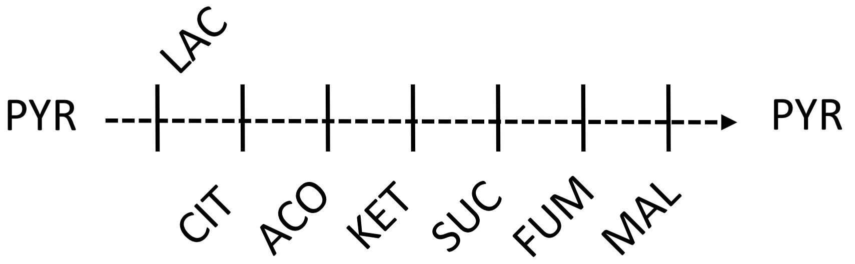

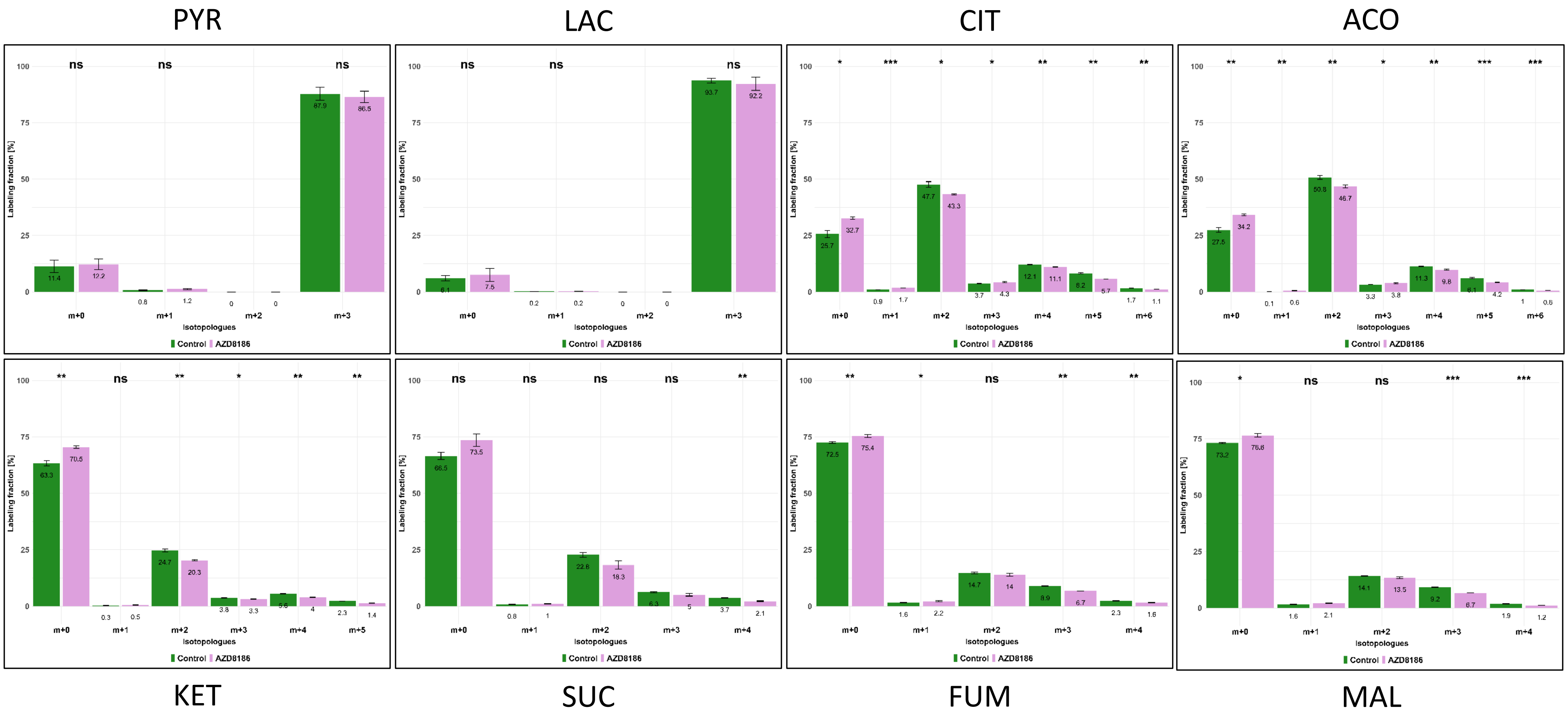

Bar charts ± standard deviation (n=3 technical replicates); * p< 0.05; ** p< 0.01; *** p<0.001 (adjusted p-value; Welch's t-test)

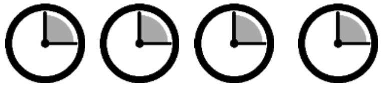

1h 3h 6h **24h**

Transition Into TCA Cycle & Following Intermediates

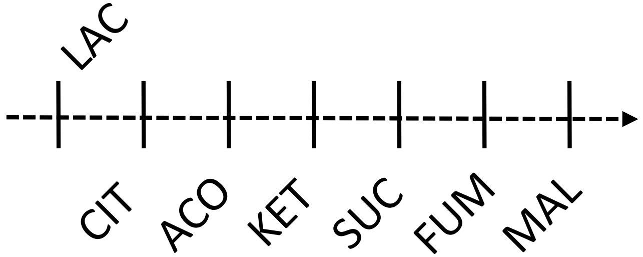

PYR LAC CIT ACO

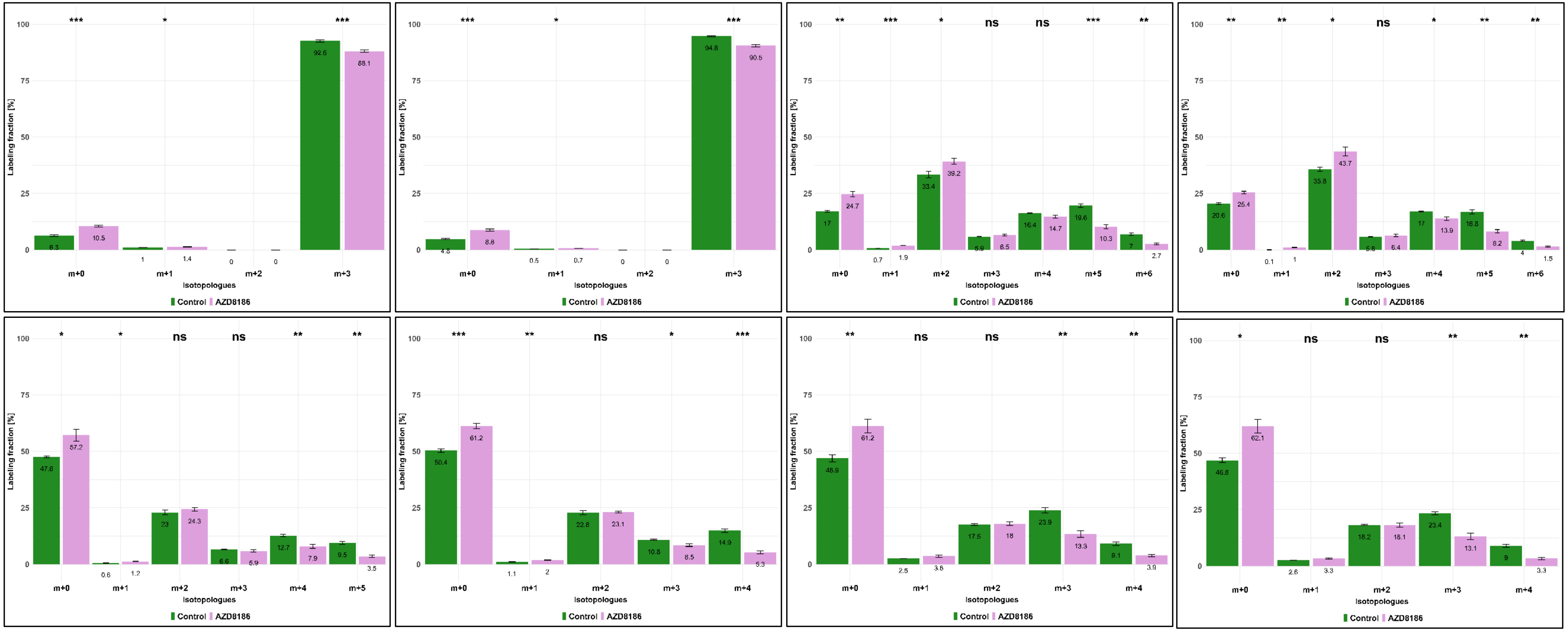

KET SUC FUM MAL

Bar charts ± standard deviation (n=3 technical replicates); * p< 0.05; ** p< 0.01; *** p<0.001 (adjusted p-value; Welch's t-test)

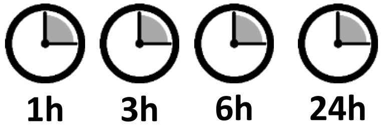

Transition Into TCA Cycle & Following Intermediates

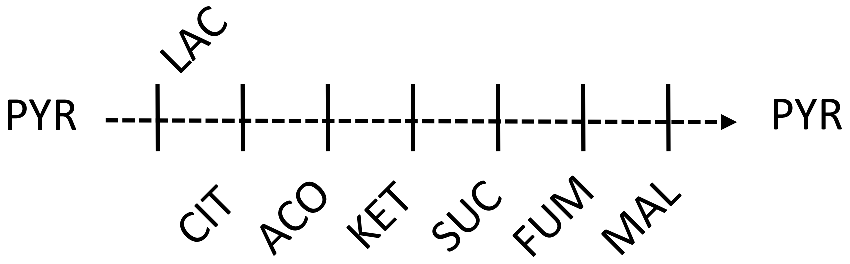

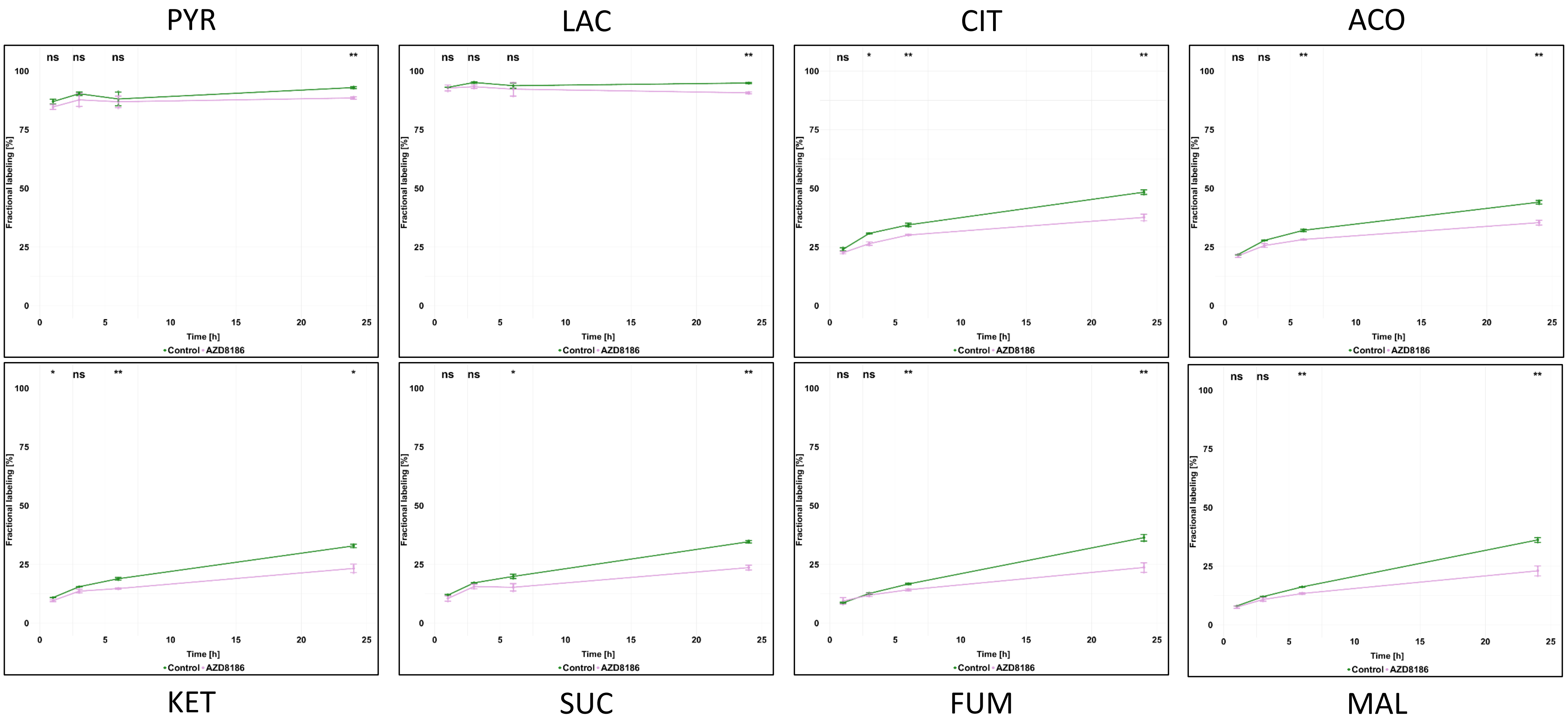

Line graphs ± standard deviation (n=3 technical replicates); * p< 0.05; ** p< 0.01; *** p<0.001 (adjusted p-value; Welch's t-test)

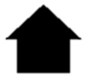

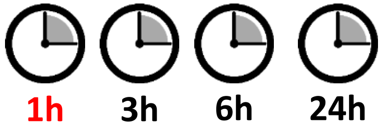

Amino Acids

Bar charts ± standard deviation (n=3 technical replicates); * p< 0.05; ** p< 0.01; *** p<0.001 (adjusted p-value; Welch's t-test)

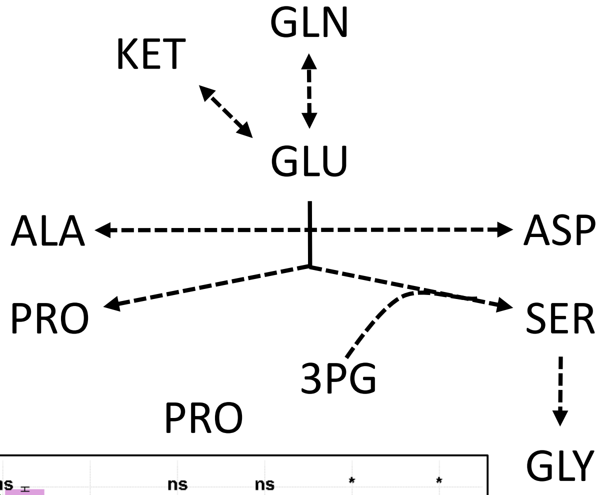

GLN GLU ALA PRO

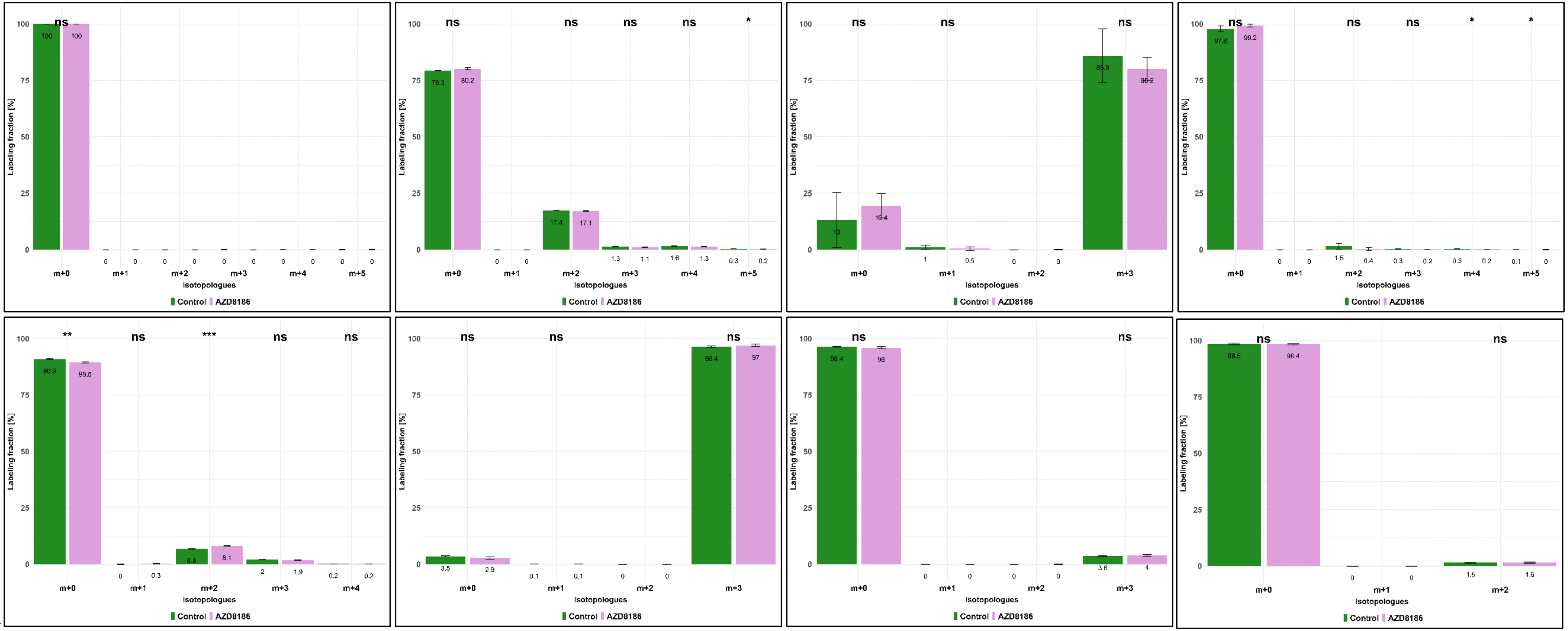

ASP 3PG SER GLY

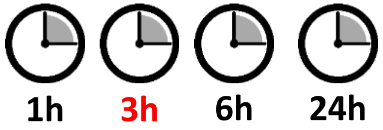

Amino Acids

Bar charts ± standard deviation (n=3 technical replicates); * p< 0.05; ** p< 0.01; *** p<0.001 (adjusted p-value; Welch's t-test)

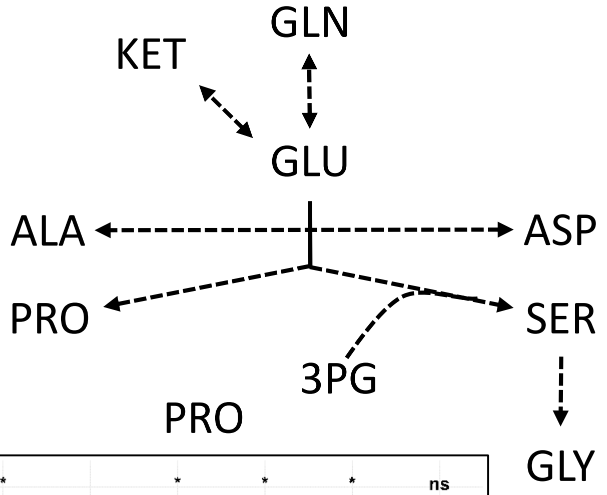

GLN GLU ALA PRO

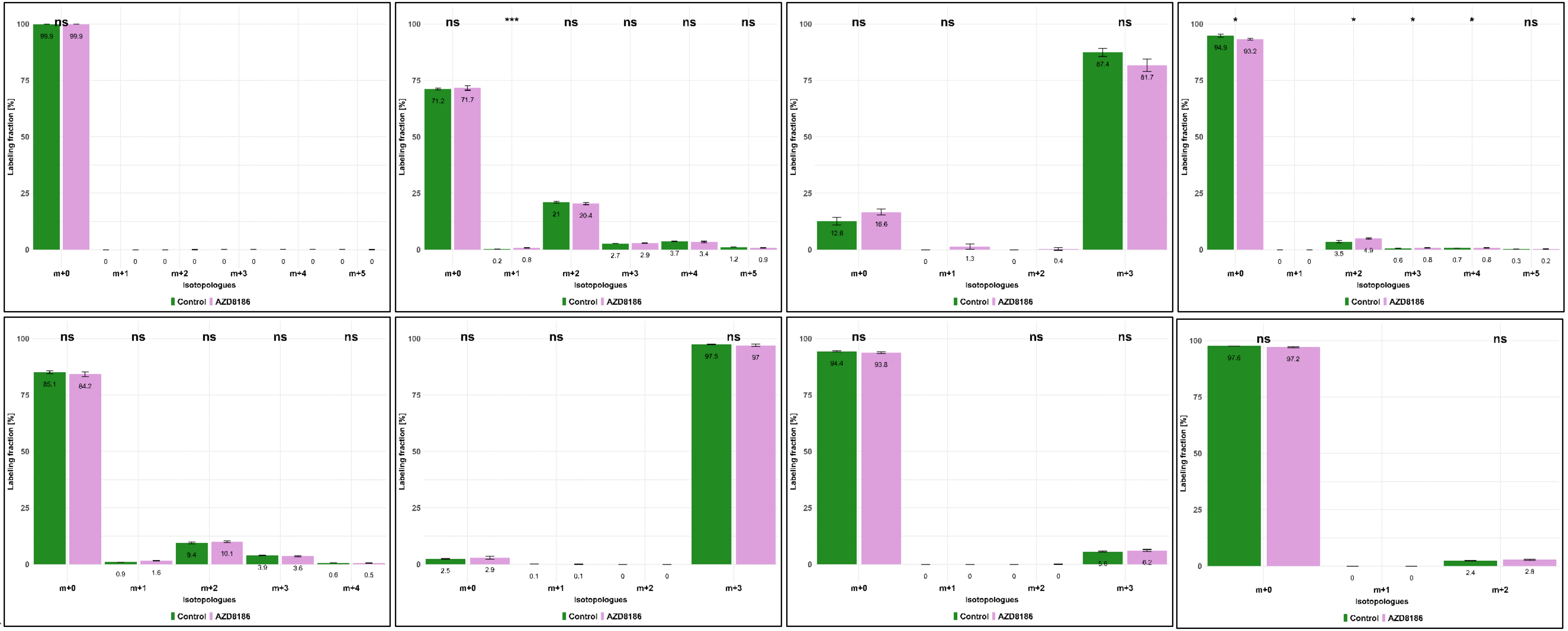

ASP 3PG SER GLY

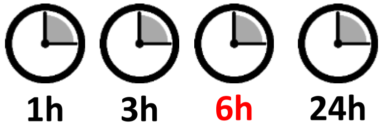

Amino Acids

Bar charts ± standard deviation (n=3 technical replicates); * p< 0.05; ** p< 0.01; *** p<0.001 (adjusted p-value; Welch's t-test)

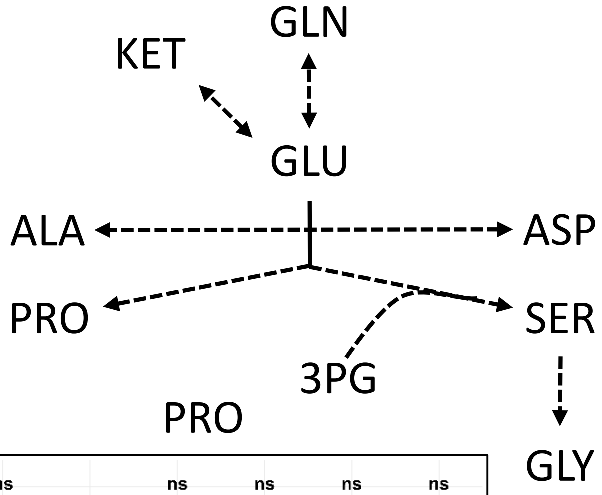

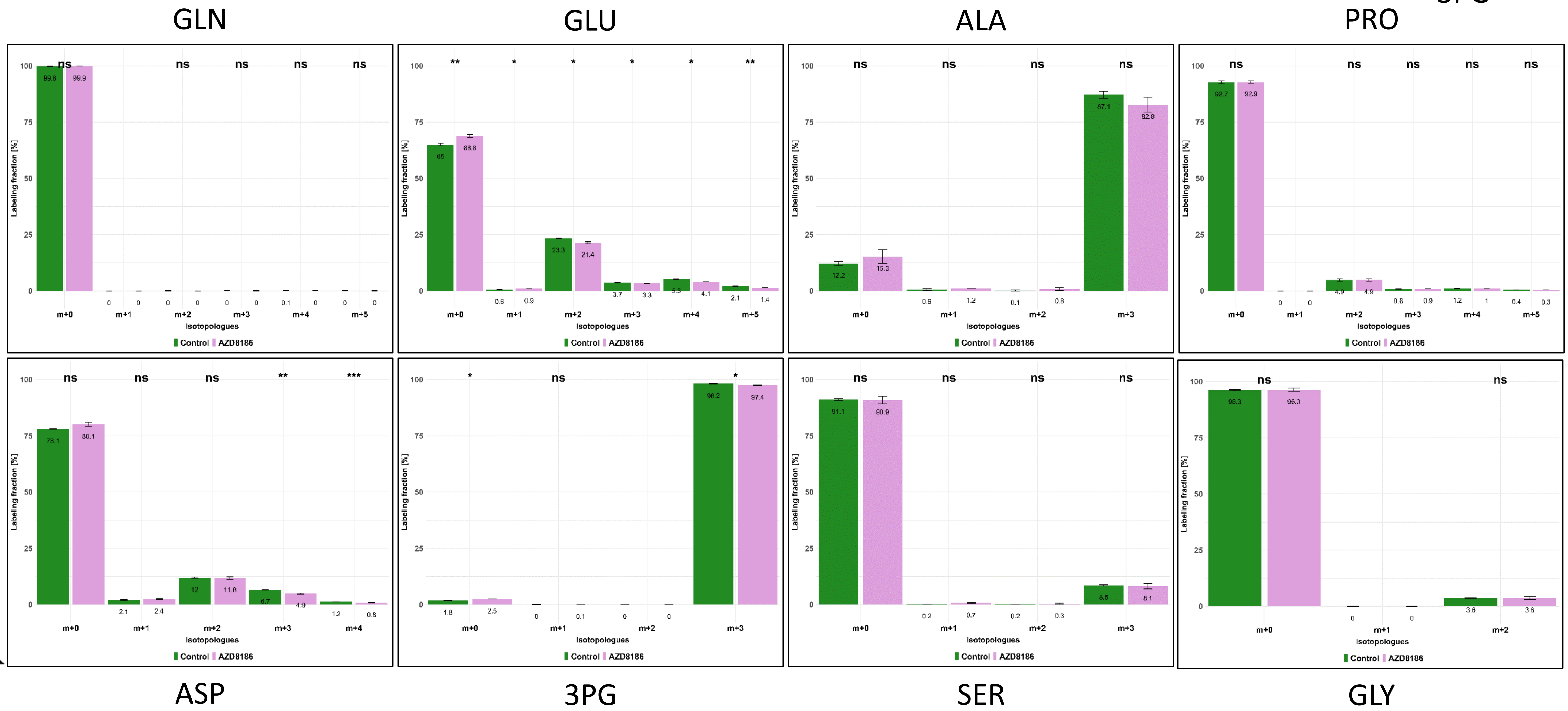

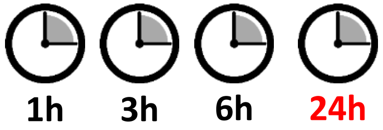

Amino Acids

Bar charts ± standard deviation (n=3 technical replicates); * p< 0.05; ** p< 0.01; *** p<0.001 (adjusted p-value; Welch's t-test)

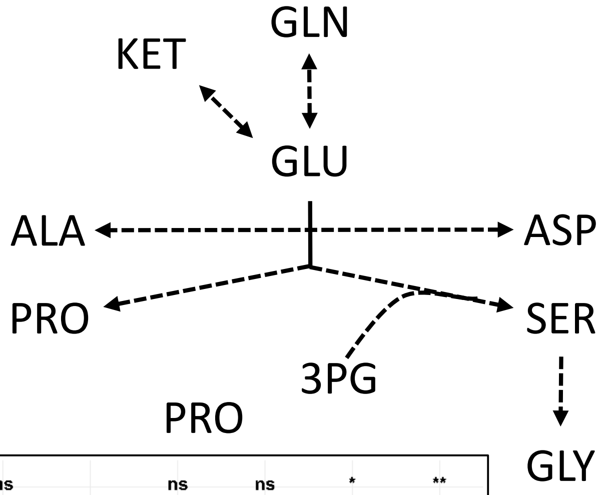

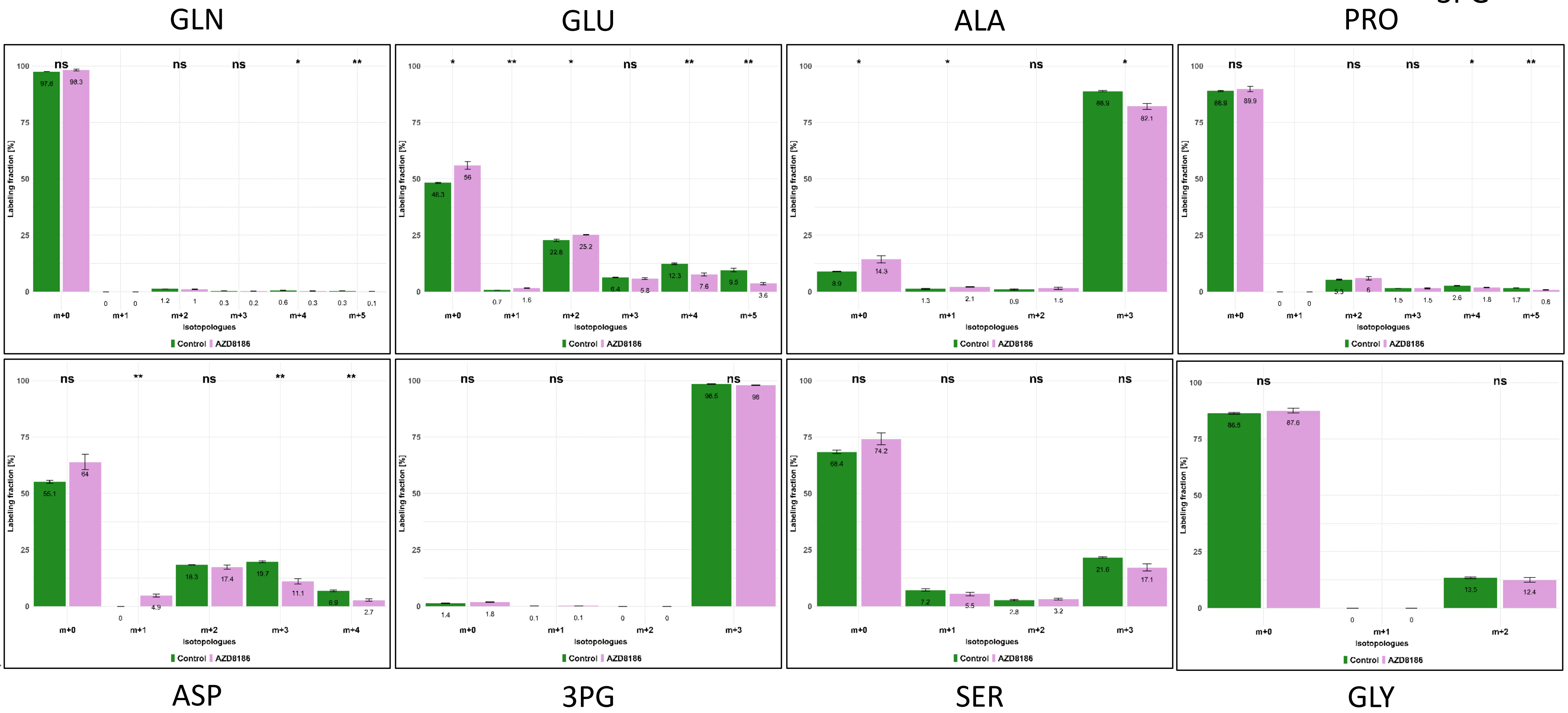

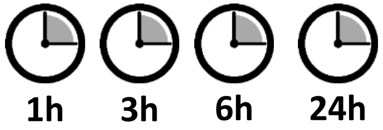

Amino Acids

Line graphs ± standard deviation (n=3 technical replicates); * $p< 0.05$; ** $p< 0.01$; *** $p<0.001$ (adjusted p-value; Welch's t-test)

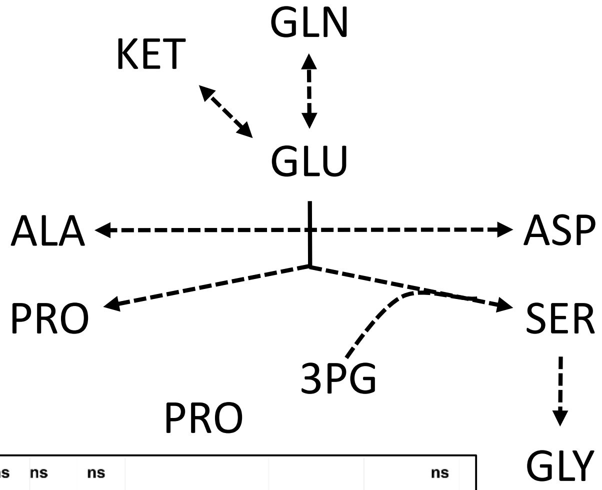

GLN

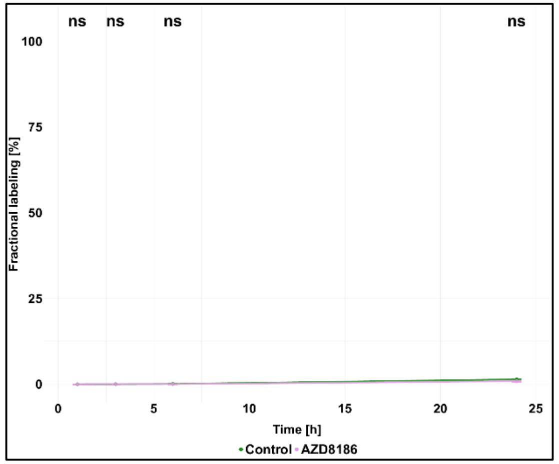

GLU

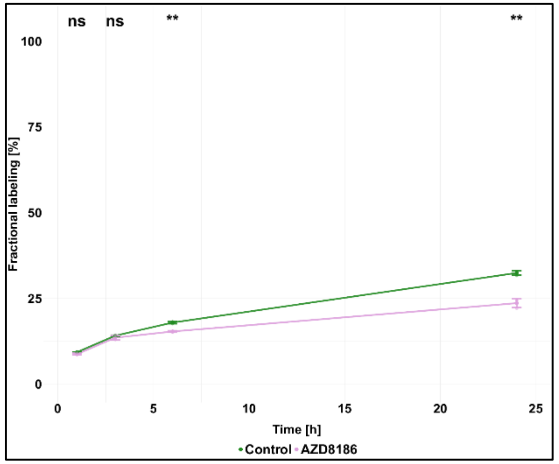

ALA

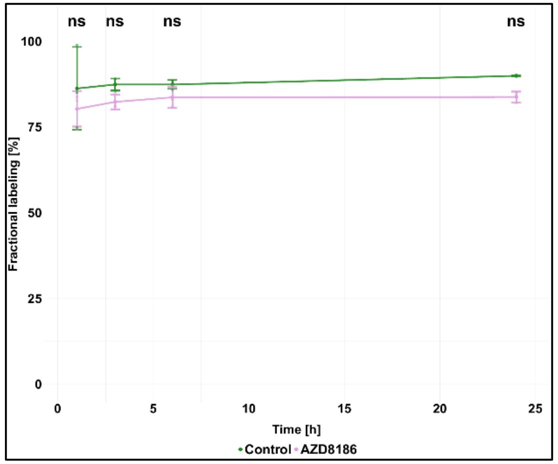

PRO

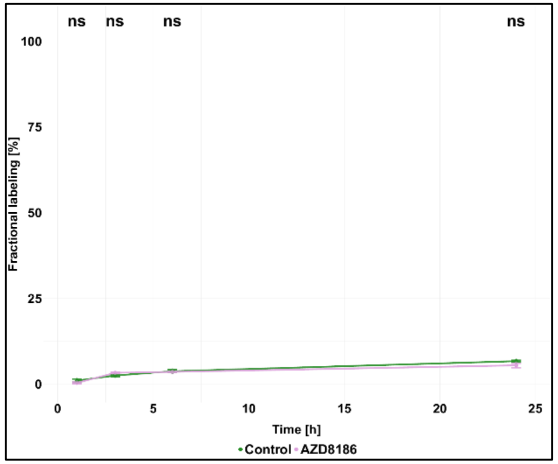

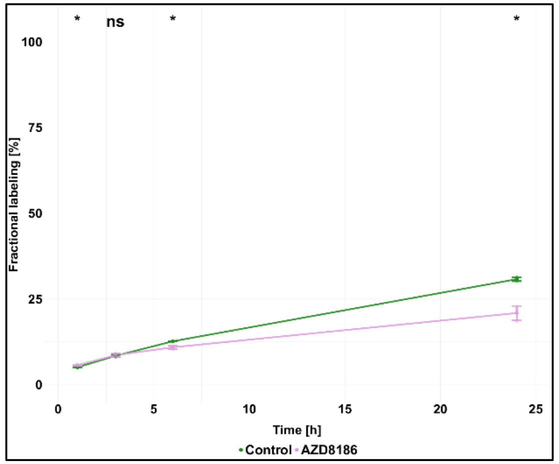

ASP

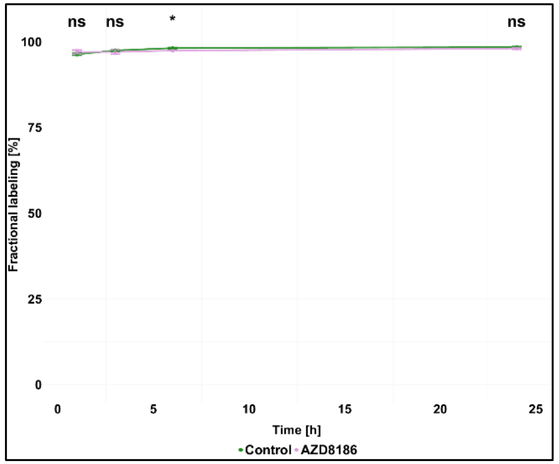

3PG

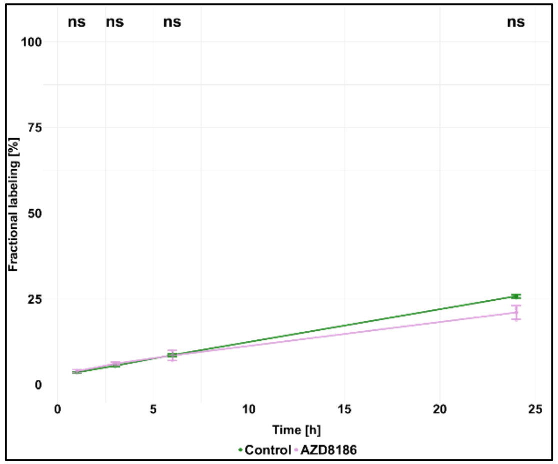

SER

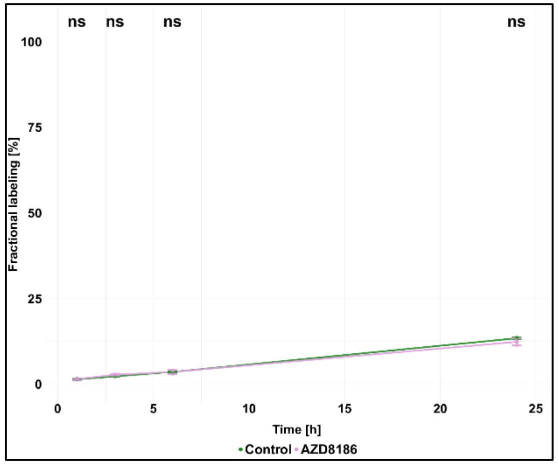

GLY

1h 3h 6h 24h

Ribonucleotides & Downstream Metabolites

AMP

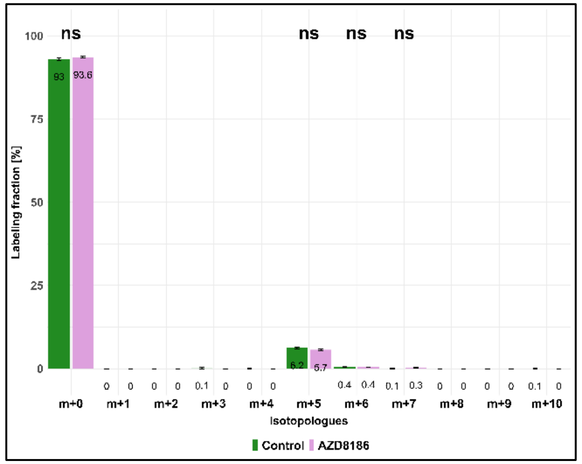

ADP

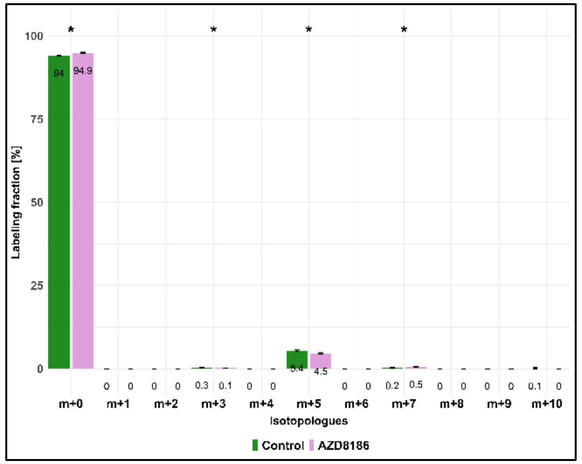

ATP

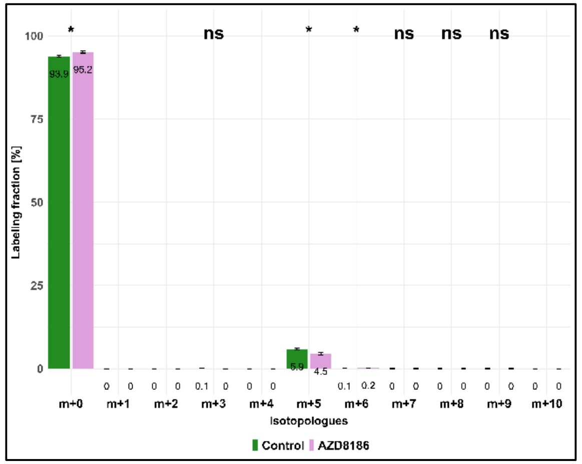

UDP

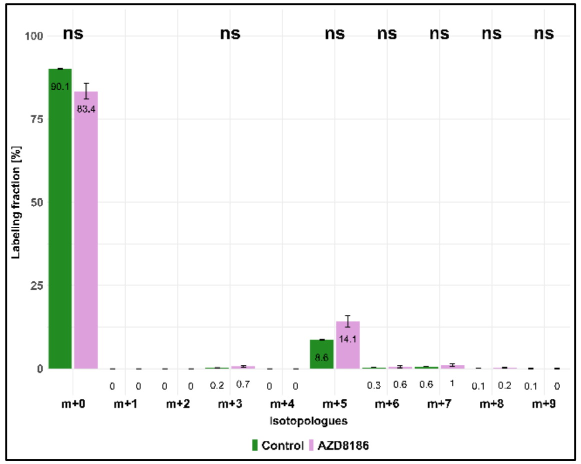

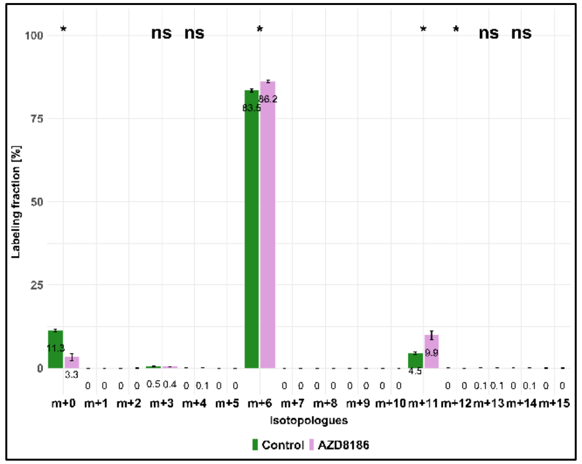

UDP-GLUC

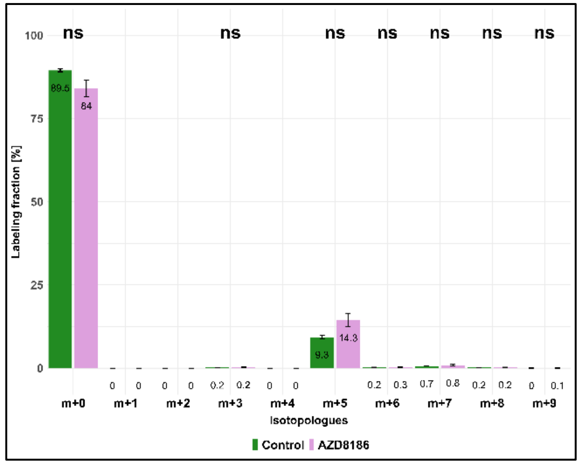

UTP

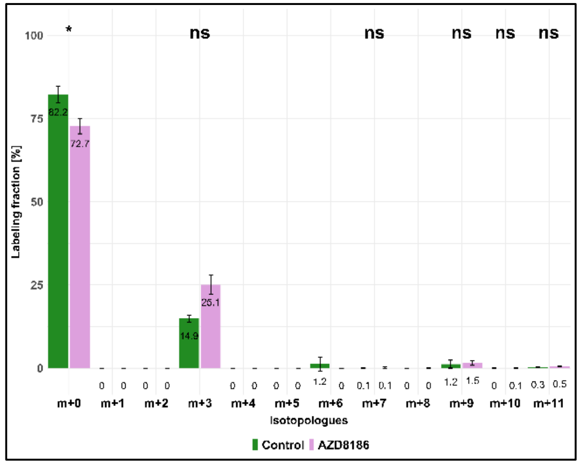

NANA

Bar charts ± standard deviation (n=3 technical replicates); * p< 0.05; ** p< 0.01; *** p<0.001 (adjusted p-value; Welch's t-test)

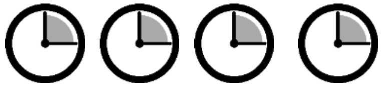

Ribonucleotides & Downstream Metabolites

AMP

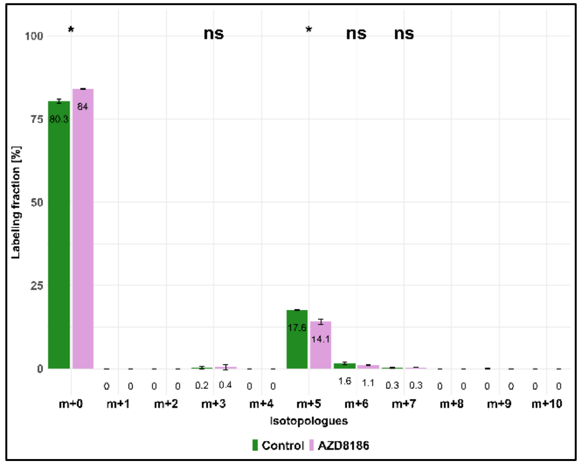

ADP

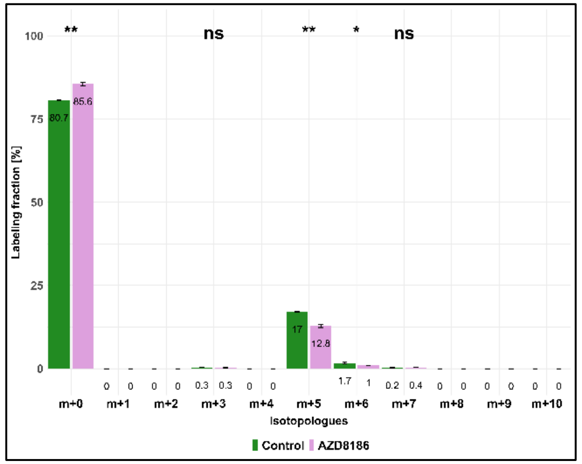

ATP

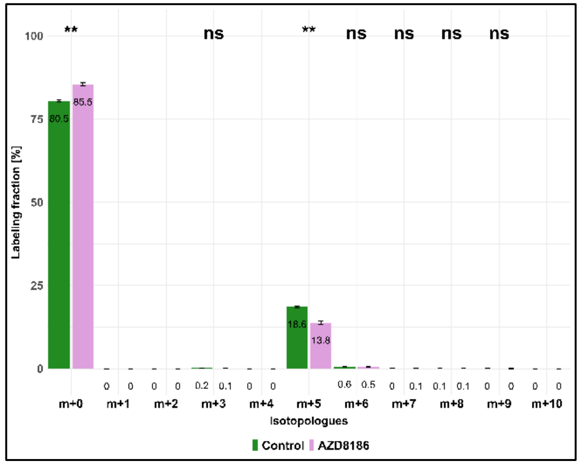

UDP

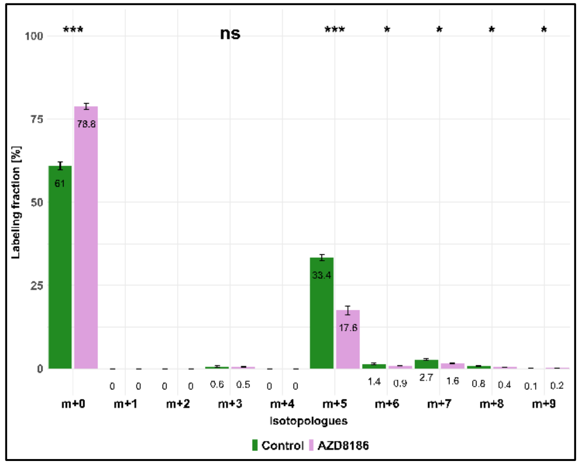

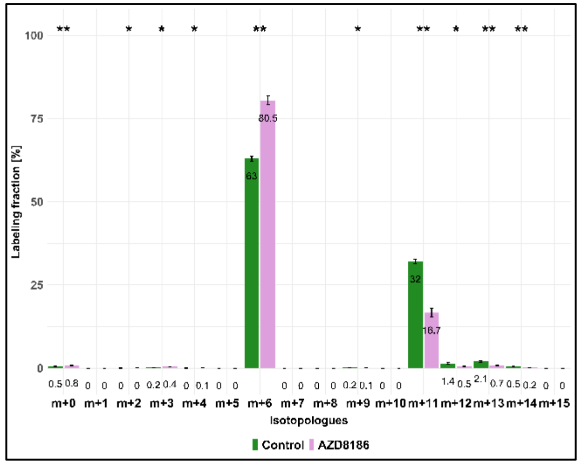

UDP-GLUC

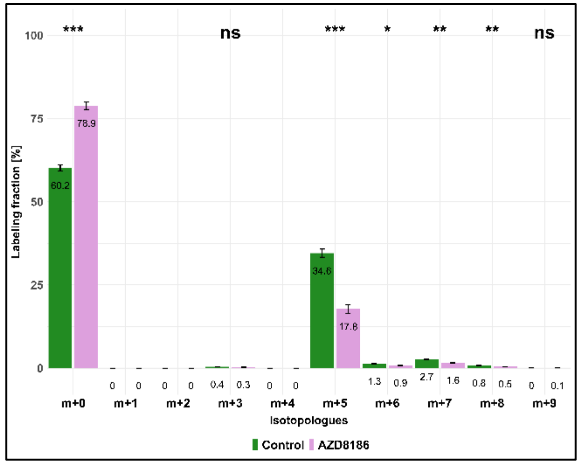

UTP

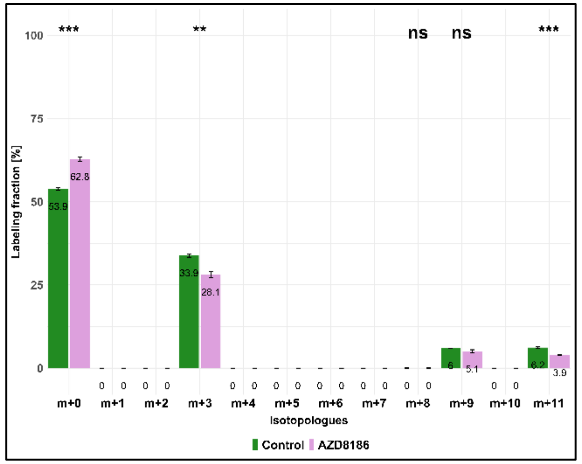

NANA

Bar charts ± standard deviation (n=3 technical replicates); * p< 0.05; ** p< 0.01; *** p<0.001 (adjusted p-value; Welch's t-test)

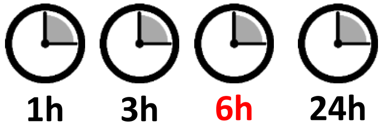

Ribonucleotides & Downstream Metabolites

AMP

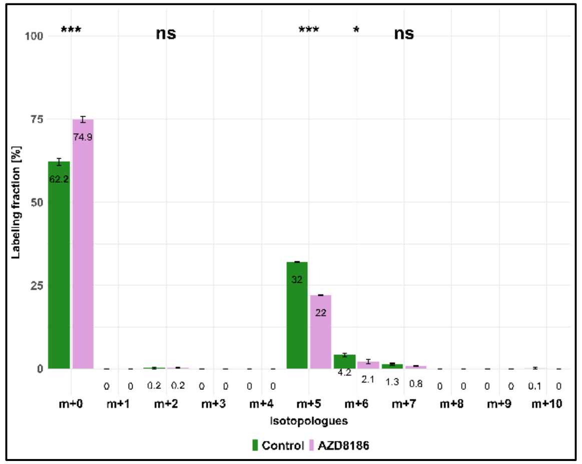

ADP

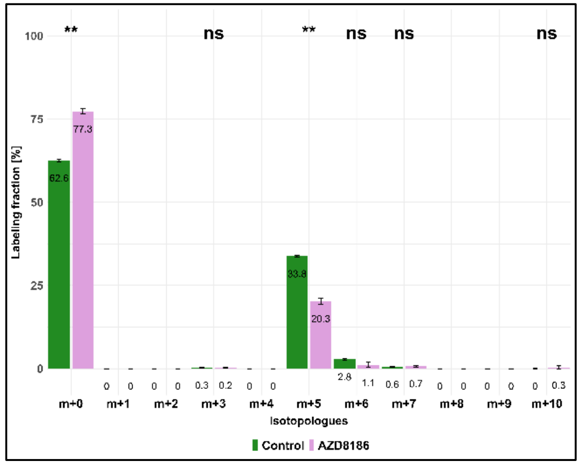

ATP

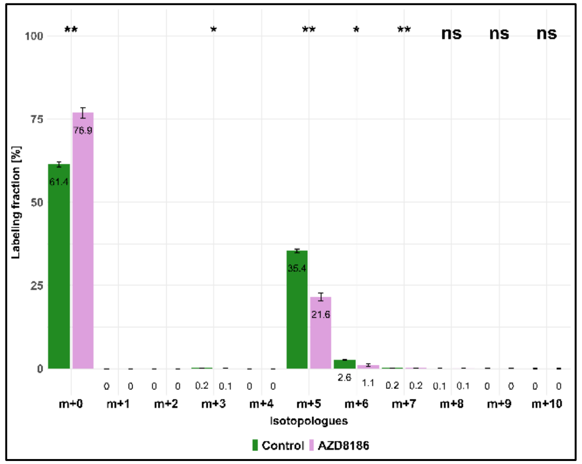

UDP

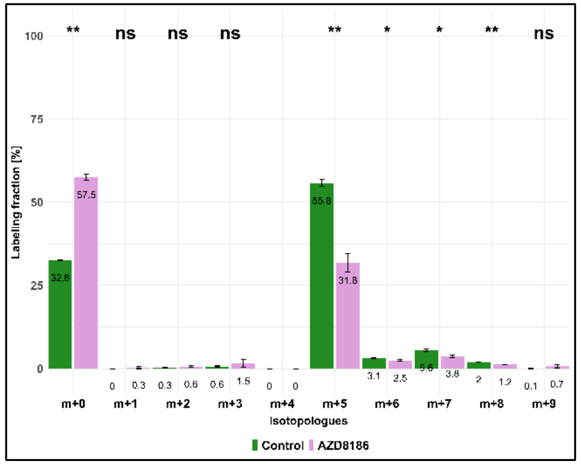

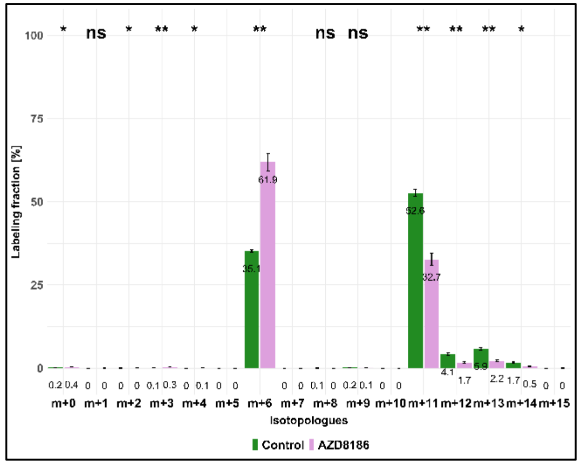

UDP-GLUC

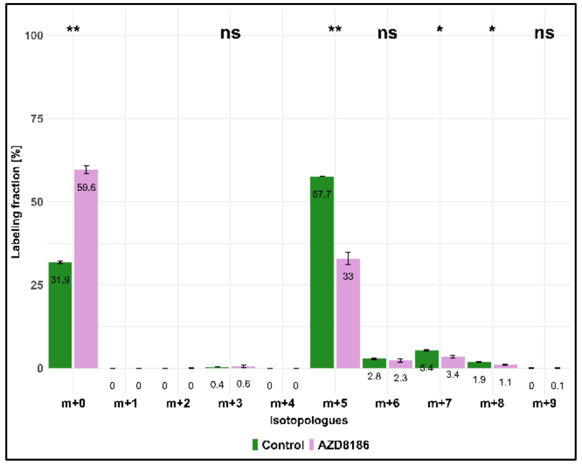

UTP

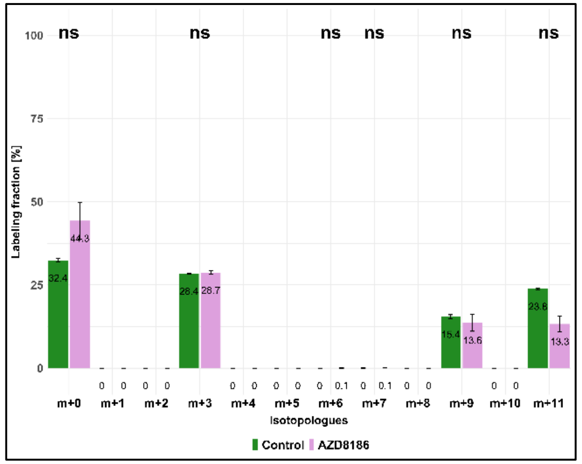

NANA

Bar charts ± standard deviation (n=3 technical replicates); * p< 0.05; ** p< 0.01; *** p<0.001 (adjusted p-value; Welch's t-test)

1h 3h 6h 24h

Ribonucleotides & Downstream Metabolites

AMP ADP ATP UDP

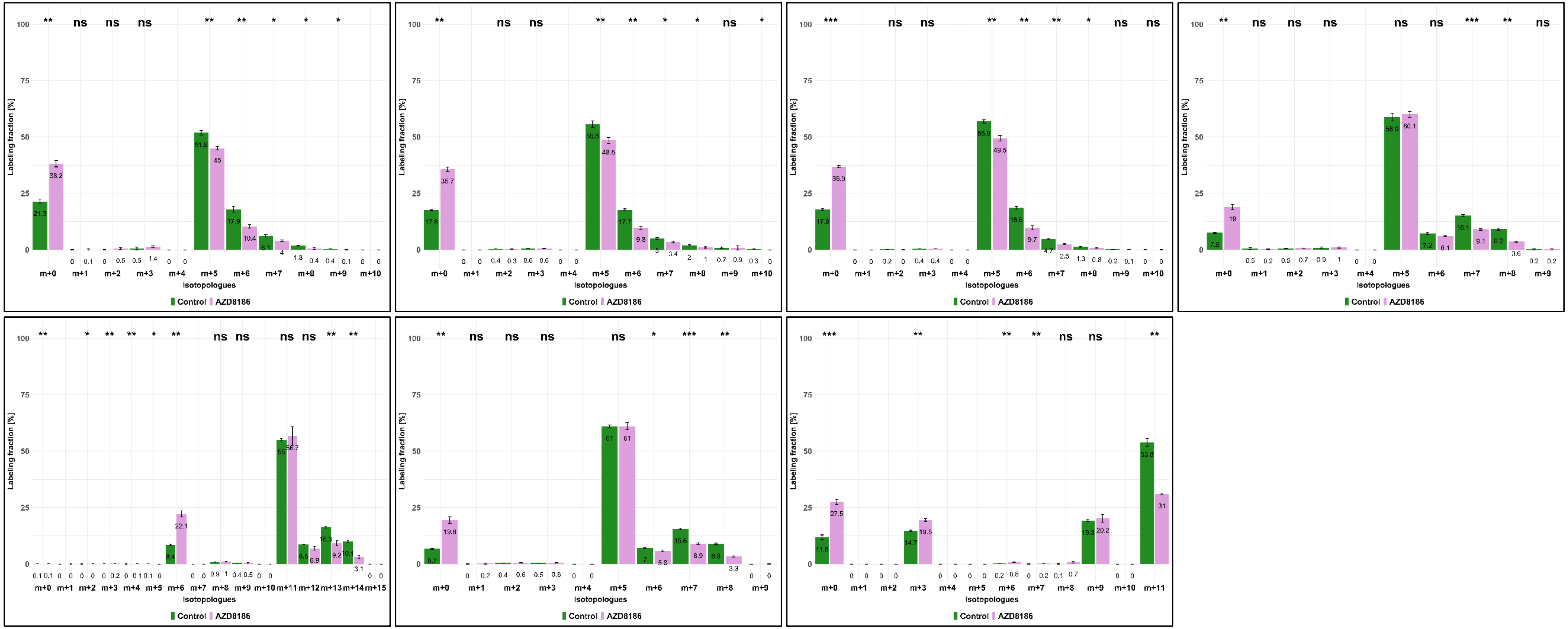

UDP-GLUC UTP NANA

Bar charts ± standard deviation (n=3 technical replicates); * p< 0.05; ** p< 0.01; *** p<0.001 (adjusted p-value; Welch's t-test)

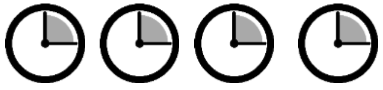

Ribonucleotides & Downstream Metabolites

AMP

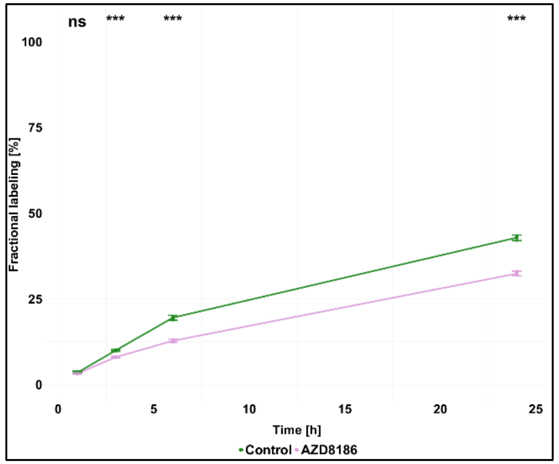

ADP

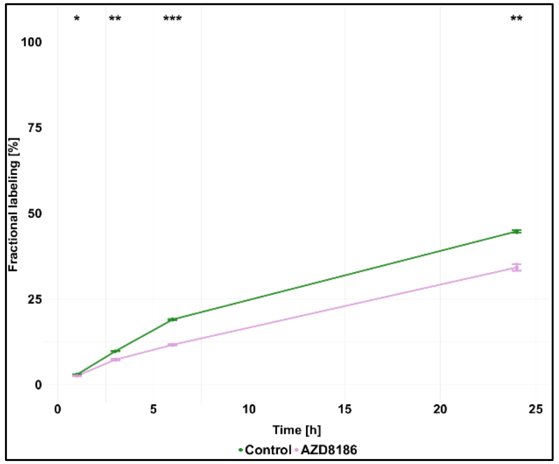

ATP

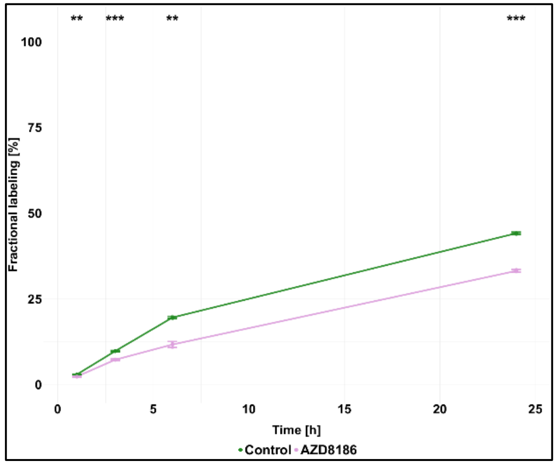

UDP

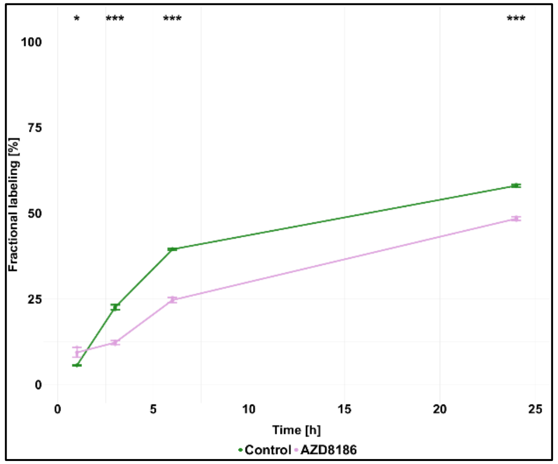

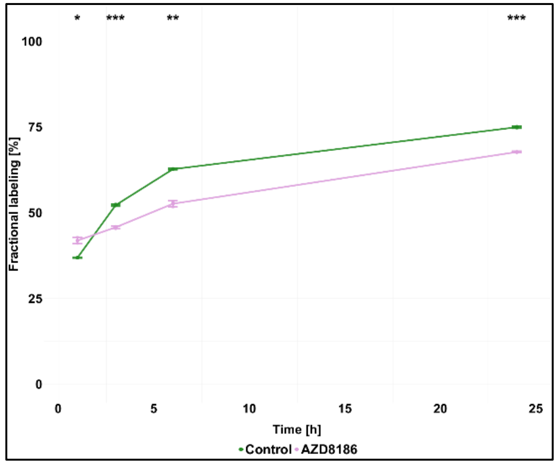

UDP-GLUC

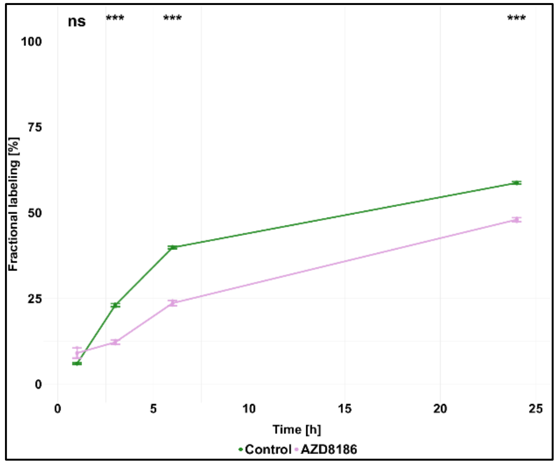

UTP

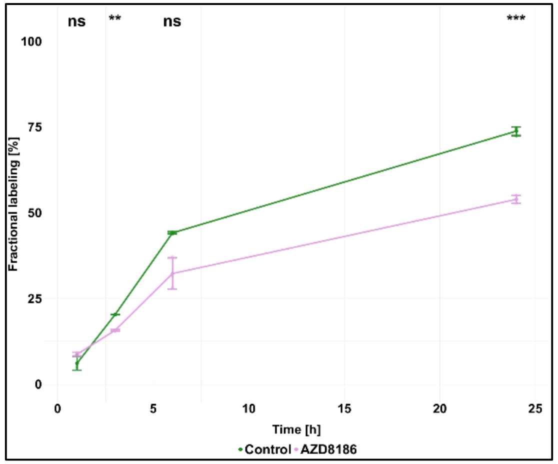

NANA

Line graphs ± standard deviation (n=3 technical replicates); * $p < 0.05$; ** $p < 0.01$; *** $p < 0.001$ (adjusted p-value; Welch's t-test)

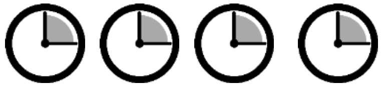

1h **3h** **6h** **24h**

Further Metabolites

3H3MG

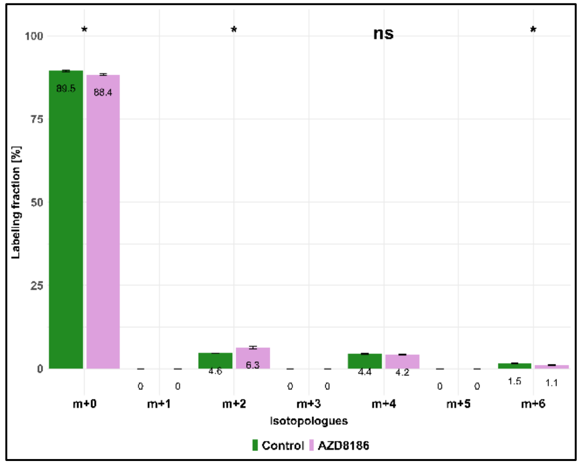

NAA

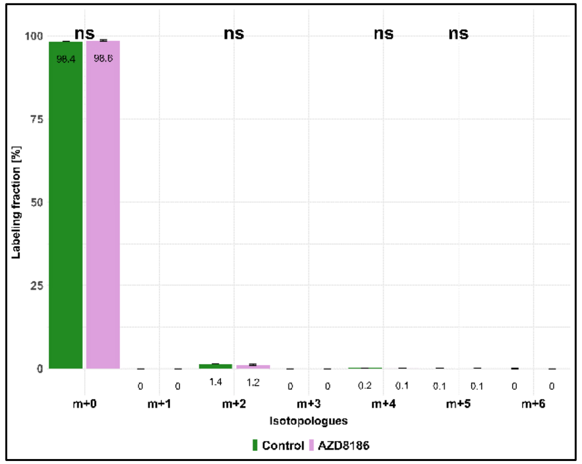

HGLU

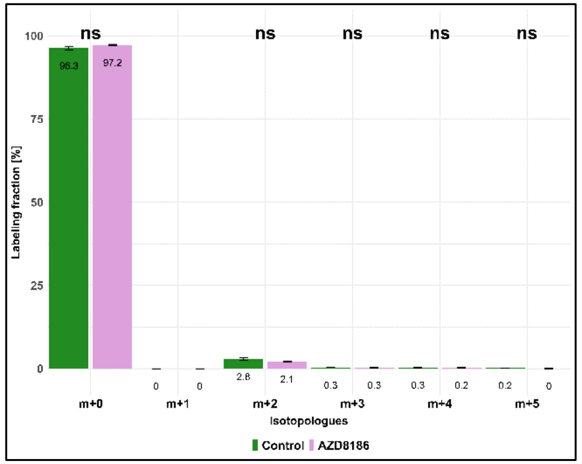

GSH

N.d.

GSSG

N.d.

UDP-GLCNAC

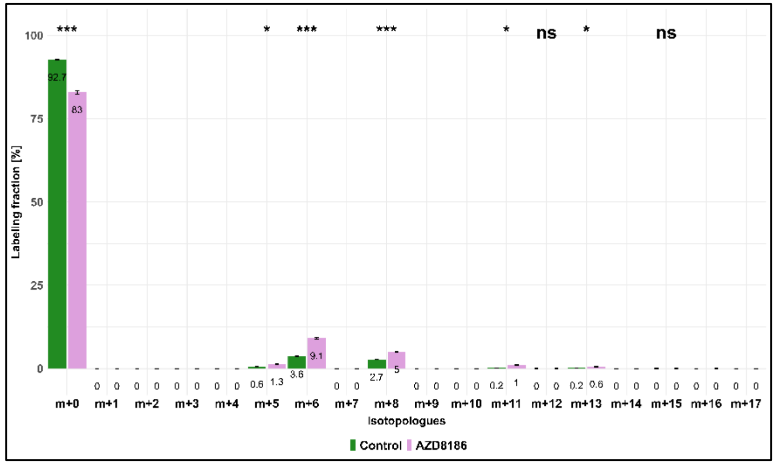

CMP-NANA

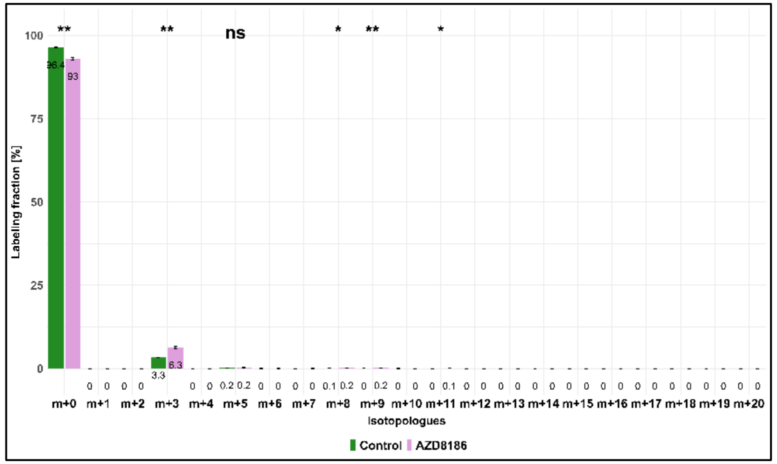

Bar charts ± standard deviation (n=3 technical replicates); * p< 0.05; ** p< 0.01; *** p<0.001 (adjusted p-value; Welch's t-test)

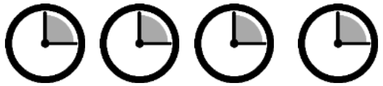

1h 3h 6h 24h

Further Metabolites

3H3MG

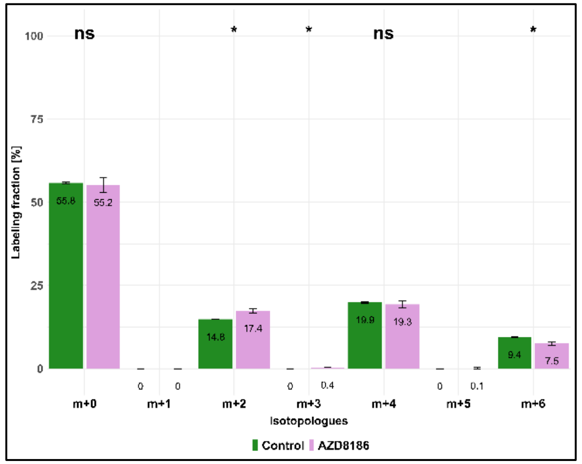

NAA

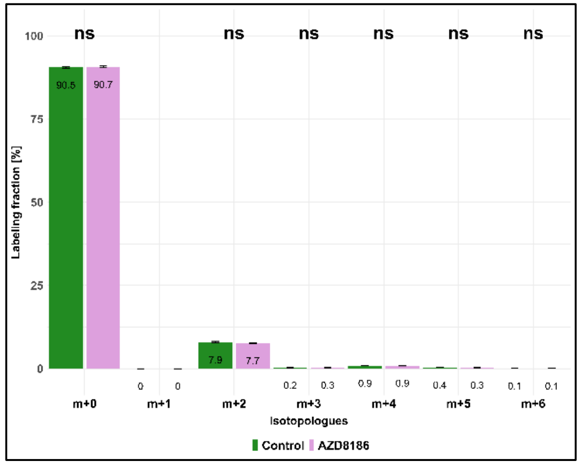

HGLU

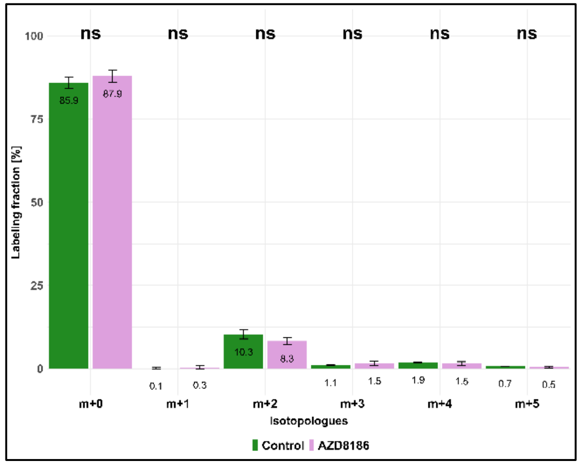

GSH

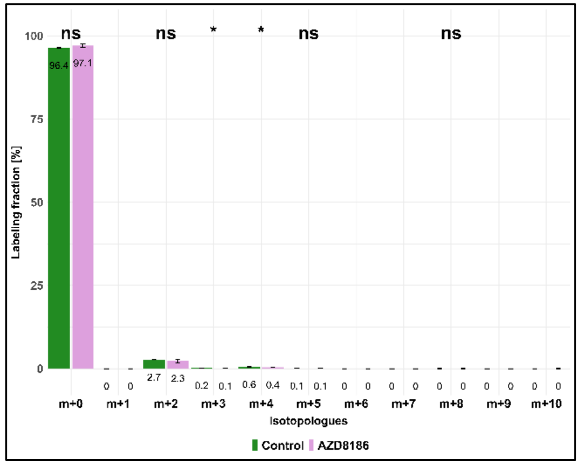

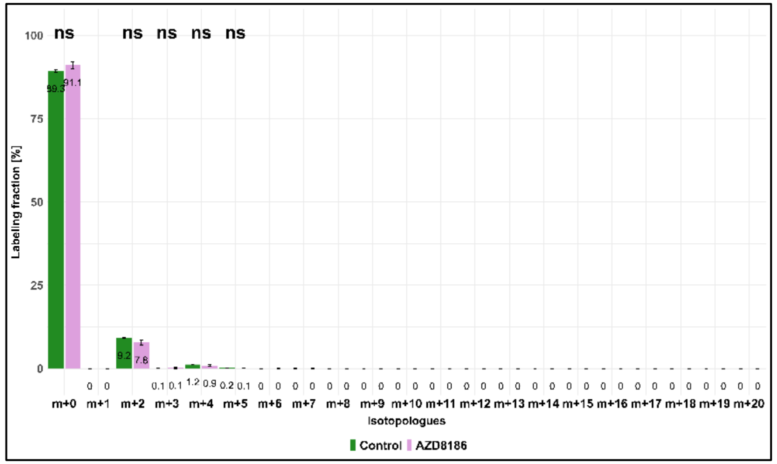

GSSG

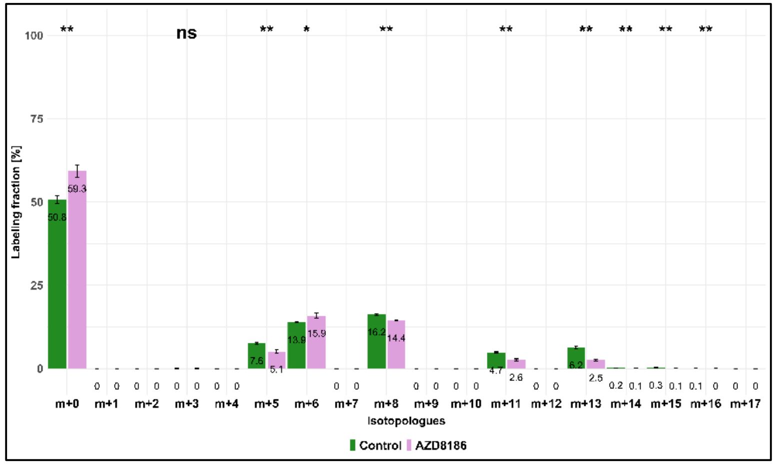

UDP-GLCNAC

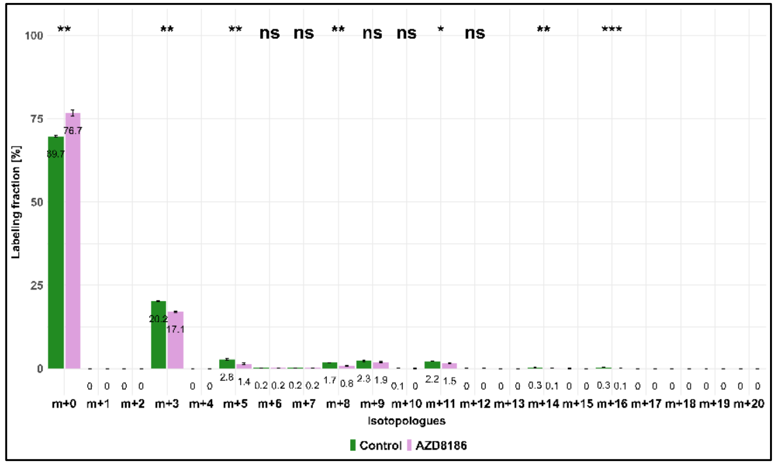

CMP-NANA

Bar charts ± standard deviation (n=3 technical replicates); * p< 0.05; ** p< 0.01; *** p<0.001 (adjusted p-value; Welch's t-test)

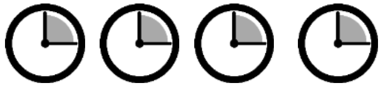

1h 3h 6h 24h

Further Metabolites

3H3MG

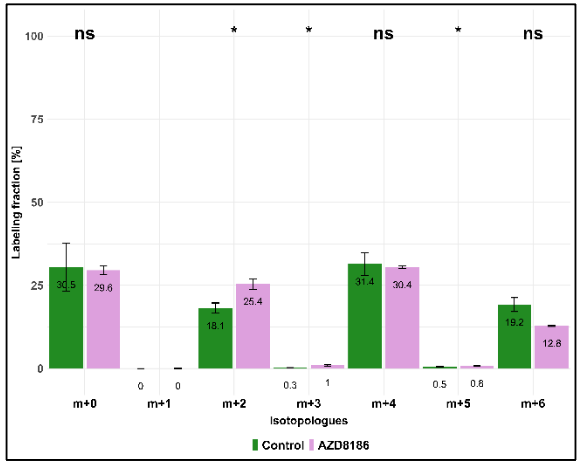

NAA

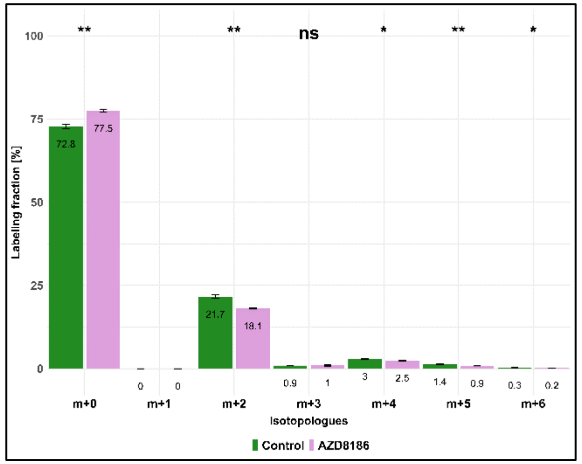

HGLU

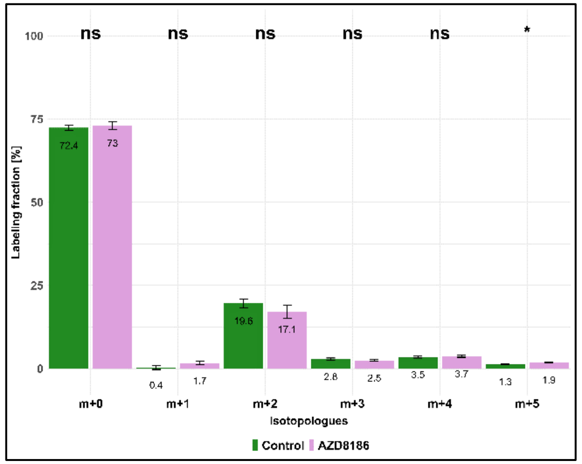

GSH

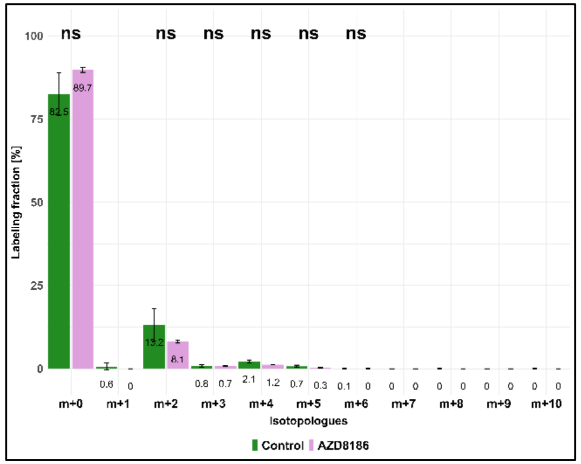

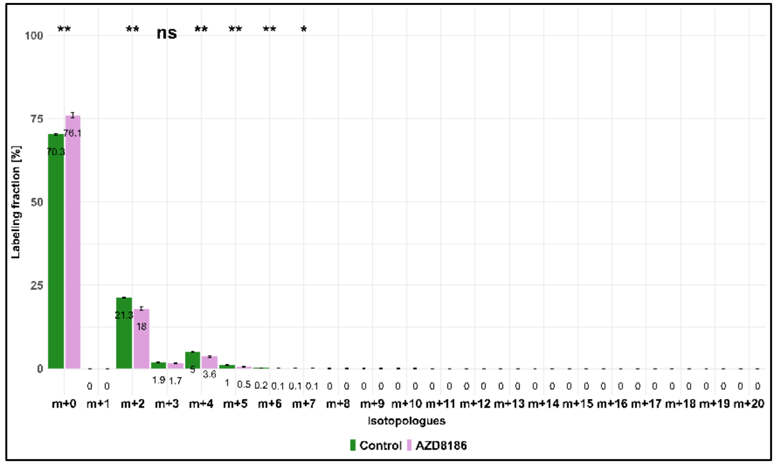

GSSG

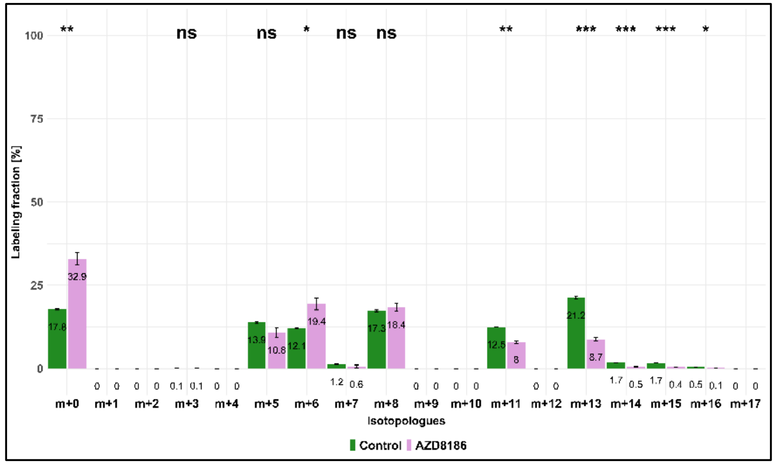

UDP-GLCNAC

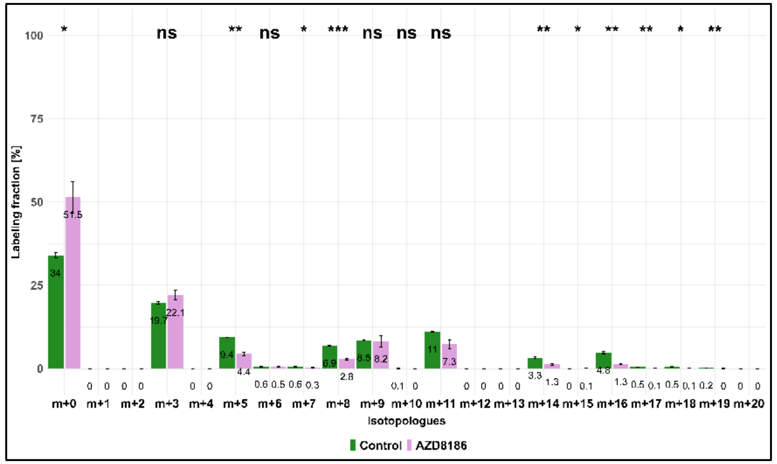

CMP-NANA

Bar charts ± standard deviation (n=3 technical replicates); * p< 0.05; ** p< 0.01; *** p<0.001 (adjusted p-value; Welch's t-test)

1h 3h 6h 24h

Further Metabolites

3H3MG

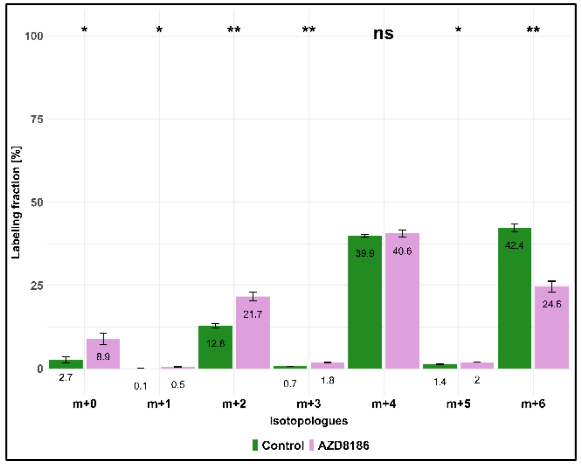

NAA

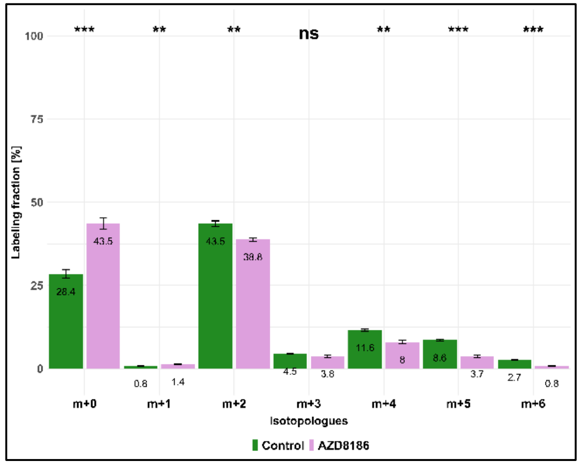

HGLU

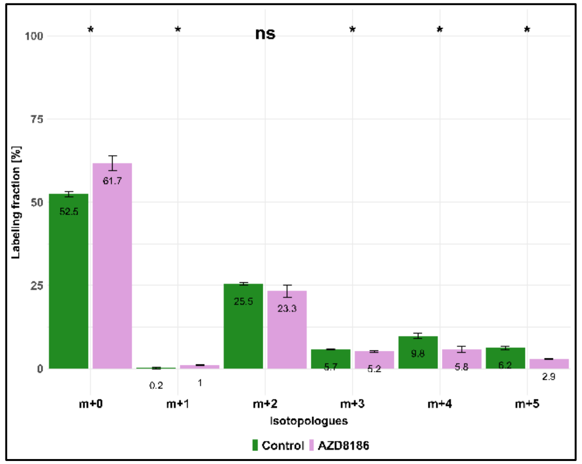

GSH

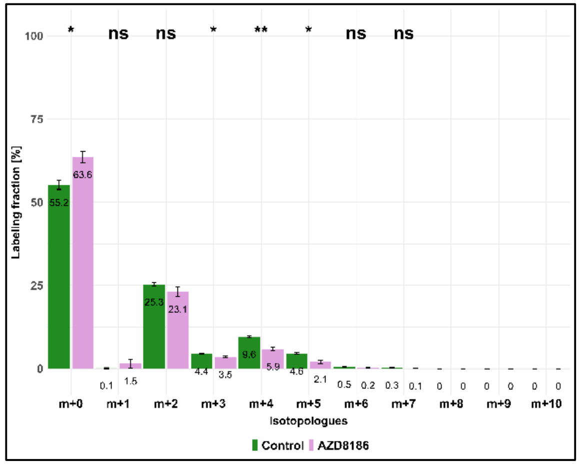

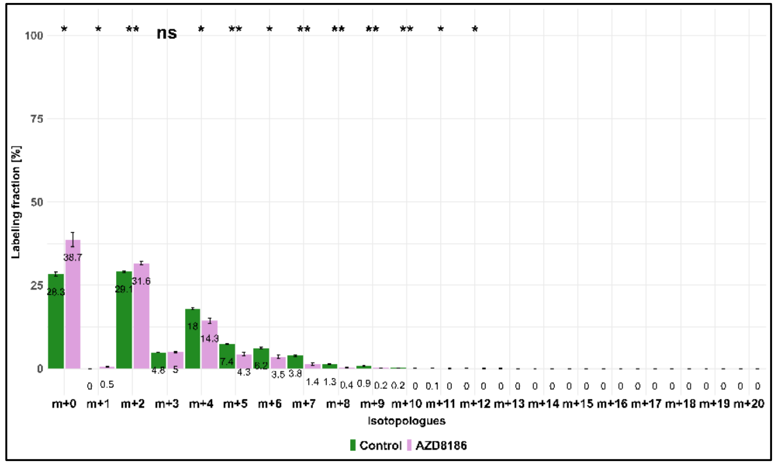

GSSG

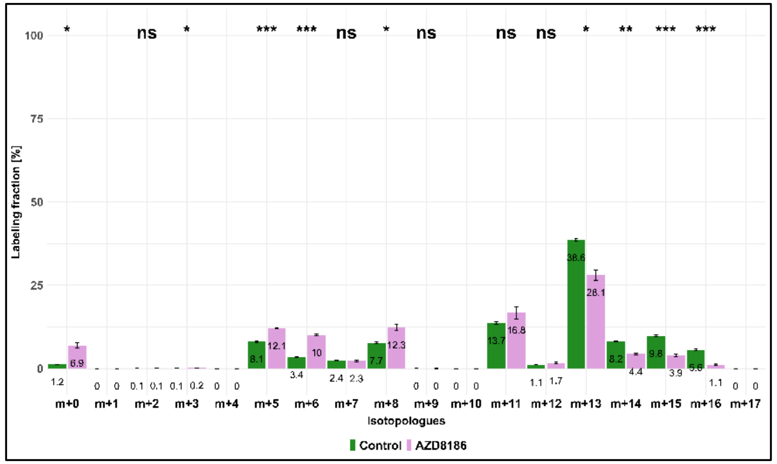

UDP-GLCNAC

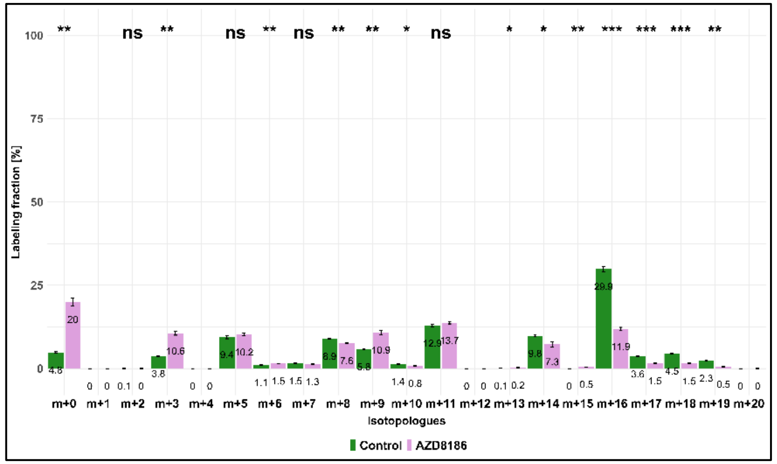

CMP-NANA

Bar charts ± standard deviation (n=3 technical replicates); * p< 0.05; ** p< 0.01; *** p<0.001 (adjusted p-value; Welch's t-test)

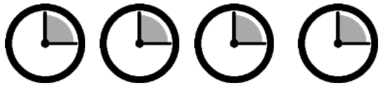

1h 3h 6h 24h

Further Metabolites

3H3MG

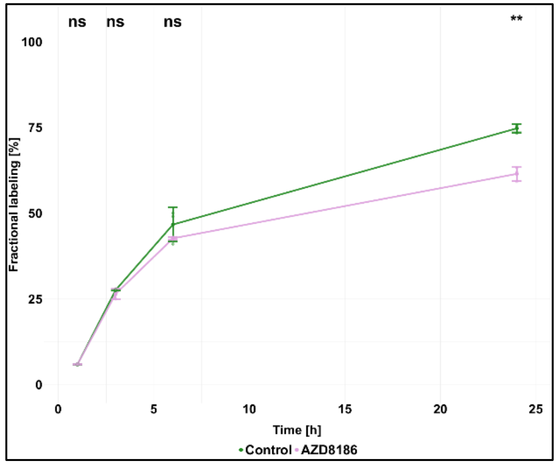

NAA

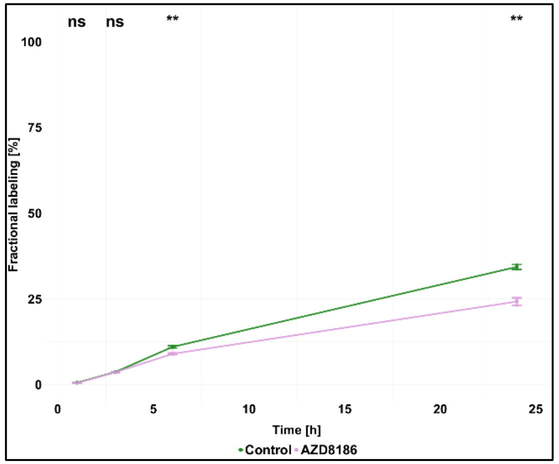

HGLU

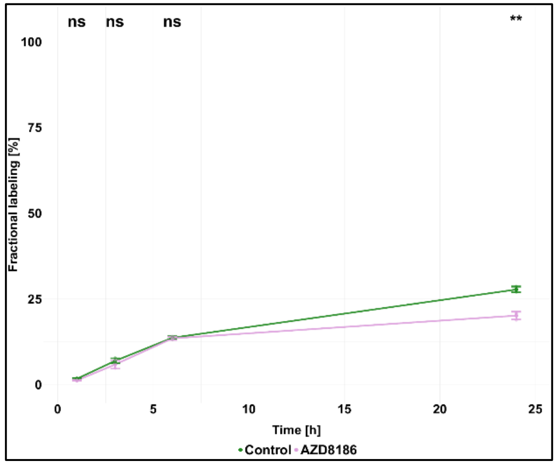

GSH

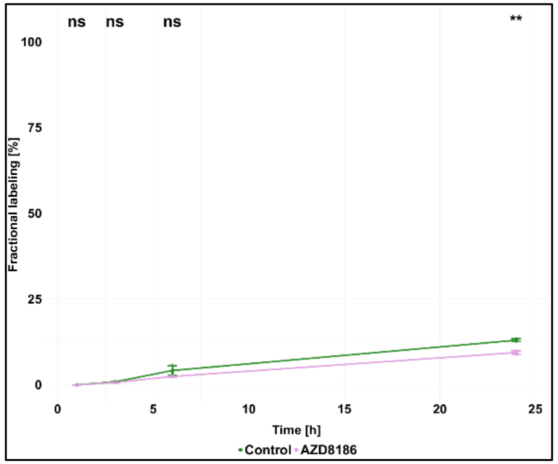

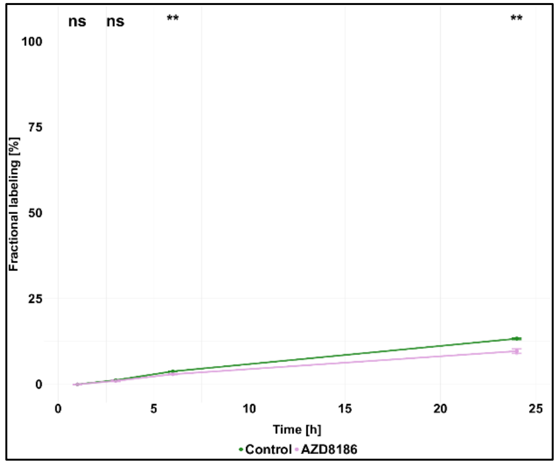

GSSG

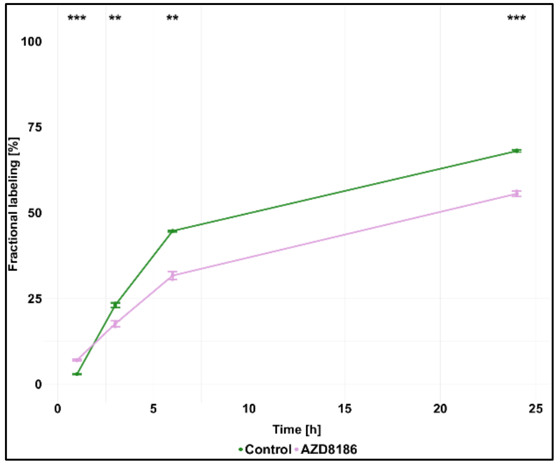

UDP-GLCNAC

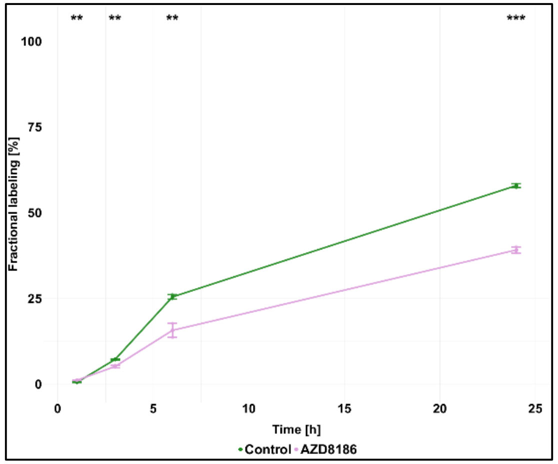

CMP-NANA

Line graphs ± standard deviation (n=3 technical replicates); * p< 0.05; ** p< 0.01; *** p<0.001 (adjusted p-value; Welch's t-test)
